# The Synthesis of Primary Amines through Reductive Amination Employing an Iron Catalyst

**DOI:** 10.1002/cssc.202000856

**Published:** 2020-05-26

**Authors:** Christoph Bäumler, Christof Bauer, Rhett Kempe

**Affiliations:** ^1^ Anorganische Chemie II - Katalysatordesign Universität Bayreuth 95440 Bayreuth Germany

**Keywords:** aldehydes, iron catalyst, ketones, primary amines, reductive amination

## Abstract

The reductive amination of ketones and aldehydes by ammonia is a highly attractive method for the synthesis of primary amines. The use of catalysts, especially reusable catalysts, based on earth‐abundant metals is similarly appealing. Here, the iron‐catalyzed synthesis of primary amines through reductive amination was realized. A broad scope and a very good tolerance of functional groups were observed. Ketones, including purely aliphatic ones, aryl–alkyl, dialkyl, and heterocyclic, as well as aldehydes could be converted smoothly into their corresponding primary amines. In addition, the amination of pharmaceuticals, bioactive compounds, and natural products was demonstrated. Many functional groups, such as hydroxy, methoxy, dioxol, sulfonyl, and boronate ester substituents, were tolerated. The catalyst is easy to handle, selective, and reusable and ammonia dissolved in water could be employed as the nitrogen source. The key is the use of a specific Fe complex for the catalyst synthesis and an N‐doped SiC material as catalyst support.

## Graphical abstract

**Iron constitution**: Impregnation of a specific Fe complex on an (N)SiC support followed by pyrolysis and reduction forms the active iron catalyst for reductive amination of carbonyl compounds. Ketones, including purely aliphatic ones, aryl–alkyl, dialkyl, and heterocyclic, as well as aldehydes can be converted smoothly into their corresponding primary amines.

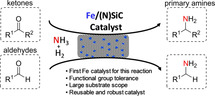

The reductive amination of ketones and aldehydes is the method of choice for the synthesis of alkyl amines from inexpensive and diversely available substrates.^1^ The synthesis of primary amines by employing ammonia as the nucleophile is especially attractive and challenging (Scheme 1, top).^1^ Amines are a very important class of chemical compounds and key functional groups in many bulk and fine chemicals,^2^ drugs,^3^ and materials.^4^ The use of catalysts based on earth‐abundant metals in reactions classically mediated by noble metals is also attractive and challenging. Significant progress has been made in the field of homogeneous earth‐abundant 3d metal catalysis in recent years.^5^, ^6^, ^7^ The use of reusable nanostructured earth‐abundant 3d metal catalysts for a broad applicability in complex organic syntheses is highly desirable and has been disclosed significantly less.^8^, ^9^ Beller and co‐workers recently introduced a reusable cobalt catalyst for the general synthesis of primary amines from aldehydes or ketones and ammonia (Scheme 1),^10^ and we discovered that a nickel catalyst supported by γ‐Al_2_O_3_ and synthesized from a specific nickel complex can be used under very mild conditions by employing ammonia dissolved in water.^9^ Aqueous ammonia is an attractive and easy‐to‐handle source of ammonia and has been used for the synthesis of primary amines in other catalytic reactions, such as hydroaminomethylation,^11^ telomerization of ammonia and butadiene,^12^ allylic substitutions,^13^, ^14^ cross couplings,^15^ benzene oxyamination,^16^ and amine alkylation.^17^ Murugesan et al. recently disclosed a different Ni catalyst for the general synthesis of primary amines through reductive amination.^18^ In addition, other reusable Co catalysts were described.^19^ However, a reusable iron catalyst for the synthesis of primary amines through reductive amination of ketones and aldehydes has not yet been disclosed, although a reusable iron‐based catalyst system for the synthesis of secondary aryl–alkyl amines^20^ and homogeneous iron catalysts for reductive amination are known.^21^

**Scheme 1:**
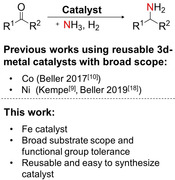
Reusable 3d metal catalyst developments with a broad scope in reductive amination of aldehydes and ketones to primary amines employing ammonia and hydrogen (R^1^ and R^2^ are aryl, alkyl substituents and, in the case of aldehydes, one of them represents a hydrogen atom).

We have recently introduced a variety of homogeneous earth‐abundant metal catalysts^22^, ^23^ and reusable nanostructured catalysts for energy storage^24^ and novel organic reactions,^25^ as well as earth‐abundant metal catalysts with a broad applicability in organic synthesis.^9^, ^26^ Herein, we report on a reusable and nanostructured iron catalyst for the general synthesis of primary amines from ketones or aldehydes through reductive amination. We employ easy‐to‐handle ammonia dissolved in water, observe a broad scope, and find that many functional groups can be tolerated. Ketones, including purely aliphatic ones, aryl–alkyl, dialkyl, and heterocyclic, as well as aldehydes can be converted smoothly into their corresponding primary amines. In addition, the amination of pharmaceuticals, bioactive compounds, and natural products is demonstrated. Many functional groups, such as hydroxy, methoxy, dioxol, sulfonyl, and boronate ester substituents, are tolerated. Our catalyst is easy to handle, selective, and reusable. The key is the use of a specific Fe complex and an N‐doped SiC material as the catalyst support. N‐doping leads to a stronger metal–support interaction during catalyst synthesis and better performance compared with non‐doped materials. The stronger metal–support interaction could enhance long‐time stability and reusability of the catalyst.^27^

Our novel iron catalyst was synthesized in a two‐step procedure (impregnation and pyrolysis) with an (N)SiC support (Figure 1 A), which was prepared by modifying a known literature procedure.^28^ For the (N)SiC support synthesis, the commercially available polycarbosilane SMP 10 and acrylonitrile were dissolved in dimethylformamide, crosslinked with azobisisobutyronitrile (AIBN) as a radical initiator for crosslinking, and pyrolyzed at 1000 °C under a nitrogen atmosphere (see the Supporting Information for more details). For the catalyst synthesis, the resulting (N)SiC support was impregnated with the Fe complex I (Figure 1 A) in acetonitrile. After removal of the solvent, the sample was pyrolyzed under a nitrogen atmosphere at 750 °C followed by a reduction step (N_2_/H_2_ 90:10) at 550 °C (see the Supporting Information). Atom absorption spectroscopy (AAS) revealed an iron content of 4.0 wt % in the Fe/(N)SiC catalyst. Nitrogen physisorption measurements (Figure S3 in the Supporting Information) of the (N)SiC support material and the catalyst showed a moderate decrease in the specific surface area [Brunauer–Emmett–Teller (BET) method] from 485 to 415 m^2^ g^−1^. The calculated pore‐size distributions were almost identical and indicated the presence of mainly micropores (92 %). The existence of metallic iron nanoparticles (cubic iron metal phase) was confirmed by powder (P)XRD (Figure S9 in the Supporting Information). TEM showed a homogeneous distribution of the iron nanoparticles over the (N)SiC support with an average particle size of 9 nm (Figure 1 B, D). The lattice spacing of an iron nanoparticle was investigated by high‐resolution (HR)TEM analysis to additionally verify the presence of metallic iron (Figure S10 in the Supporting Information). The averaged lattice plane distance of the nanoparticle was determined to be 0.2022 nm, which is in good agreement with the literature values. Additionally, the Fe/(N)SiC catalyst was investigated by X‐ray photoelectron spectroscopy (XPS), indicating the presence of metallic and oxidic iron species on the nanoparticle surface (Figure 1 C). Further investigations were performed by SEM combined with energy‐dispersive X‐ray (EDX) element maps and revealed that the iron nanoparticles were homogeneously distributed over the entire support material (Figure S4 in the Supporting Information).

**Figure 1:**
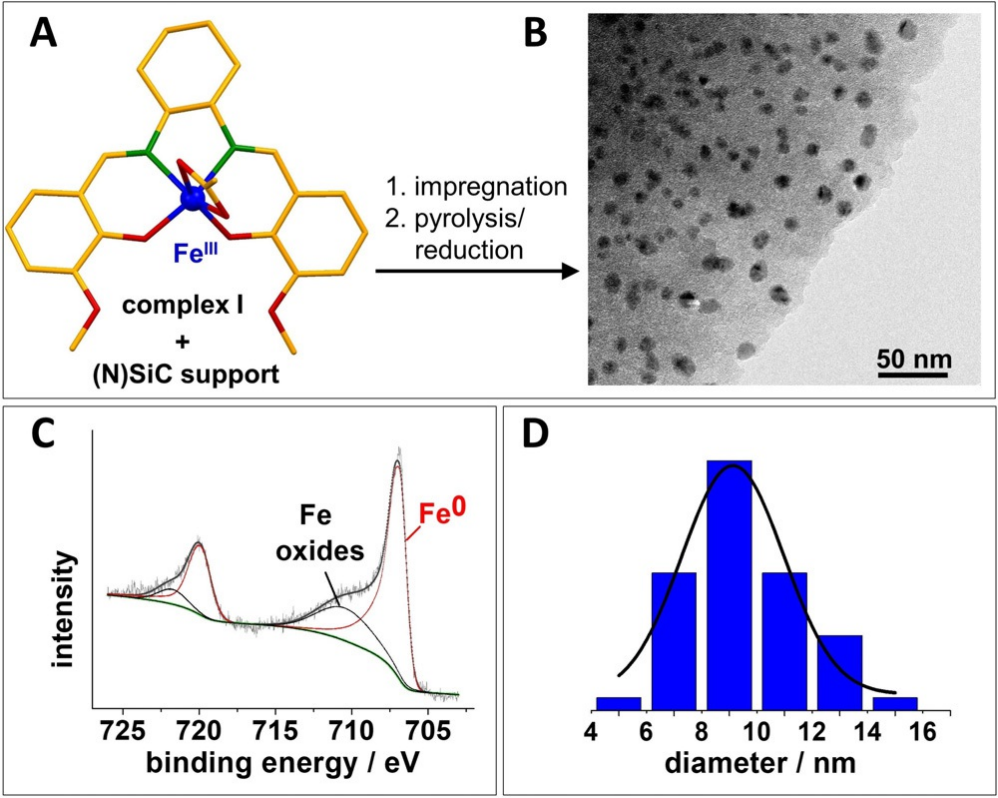
Synthesis and characterization of the Fe catalyst: A) Synthesis of the iron catalyst by wet impregnation of complex I (molecular structure determined by X‐ray single crystal structure analysis, color code: green=N, red=O, orange=C) on the (N)SiC support, followed by pyrolysis and hydrogen treatment (reduction). B) TEM analysis suggested the presence of homogeneously distributed Fe nanoparticles. C) XPS analysis confirmed the presence of metallic iron and iron oxide at the surface of the nanoparticles. D) Size distribution analysis revealed an average iron nanoparticle size of 9 nm.

The synthesis of 1‐phenylethanamine from acetophenone and aqueous ammonia was chosen as the benchmark reaction for optimizing the reaction conditions. The solvent screening showed a higher yield with increasing polarity (Table S2 in the Supporting Information) and, consequently, water was chosen as the best solvent. Next, the amount of ammonia was screened. It was found that the quantity of ammonia strongly influences the yield by reducing self‐coupling reactions of the carbonyl compound (Tables S3 and S4 in the Supporting Information). In summary, the optimized conditions for the Fe/(N)SiC catalyst are 3.5 mL of 25 % aqueous ammonia, 6.5 MPa H_2_, and 140 °C (Table S5 in the Supporting Information). Various commercially available supports and iron sources were tested in the amination of acetophenone to ensure that our combination of support material and metal complex is the most effective catalyst system in the reductive amination of carbonyl compounds to primary amines (Table 1). Surprisingly, catalysts based on support materials that were recently applied successfully for nickel catalysts (Al_2_O_3_, SiO_2_) showed no activity. Catalysts based on activated carbon, CeO_2_, and pyrolyzed polyacrylonitrile (PAN) showed low activity, whereas TiO_2_ was not suitable at all for this reaction. The complexes in Figure 2 (see the Supporting Information for more details) were tested as metal sources for the Fe/(N)SiC synthesis and also showed good yields, up to 68 % in the case of complex IV. Therefore, complex I generated the most active catalyst system with a yield of 72 % (Table 1, entries 3, 5–9). A significant decrease of the yield was observed compared with complex I by replacing the metal source with the commercially available iron complex Fe(acac)_3_ [acacH=(*Z*)‐4‐hydroxypent‐3‐en‐2‐one] or a common salt Fe^III^ nitrate [Fe(NO_3_)_3_⋅9 H_2_O]. TEM investigations of all catalysts based on (N)SiC in combination with the different complexes and of complex I in combination with the different supports (see the Supporting Information) revealed that the combination of complex I and (N)SiC gave rise to a rather well‐defined nanostructured catalyst. All other catalysts were significantly less well defined.

**Table 1:** Catalyst screening for the reductive amination of acetophenone.^[a]^

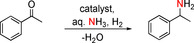				
Entry	Metal source	Support material	Pyrolysis temperature [°C]	Yield [%]
1^[b]^	complex I	(N)SiC	750	99
2	complex I	(N)SiC	650	54
3	complex I	(N)SiC	750	72
4	complex I	(N)SiC	850	51
5	complex II	(N)SiC	750	65
6	complex III	(N)SiC	750	47
7	complex IV	(N)SiC	750	68
8	complex V	(N)SiC	750	63
9	complex VI	(N)SiC	750	61
10	complex I	activated carbon	750	37
11	complex I	SiO_2_	750	0
12	complex I	TiO_2_	750	0
13	complex I	CeO_2_	750	25
14	complex I	Al_2_O_3_	750	0
15	complex I	pyrolyzed PAN	750	36
16^[c]^	–	(N)SiC	1000	0
17	Fe(acacH)_3_	(N)SiC	750	42
18	Fe(NO)_3_	(N)SiC	750	44

[a] Reaction conditions: Fe (8.6 mol %, 60 mg supported Fe catalyst with 4.0 wt % Fe loading, 0.043 mmol Fe, 2.4 mg Fe), acetophenone (0.5 mmol), 140 °C, 20 h, 6.5 MPa H_2_, aq. NH_3_‐25 % (3.5 mL). Yields were determined by GC with *n*‐dodecane as an internal standard. [b] 10 mol % Fe [70 mg Fe/(N)SiC with 4.0 wt % Fe loading, 0.05 mmol Fe, 2.8 mg Fe]. [c] 60 mg (N)SiC; acacH=(*Z*)‐4‐hydroxypent‐3‐en‐2‐one.

**Figure 2:**
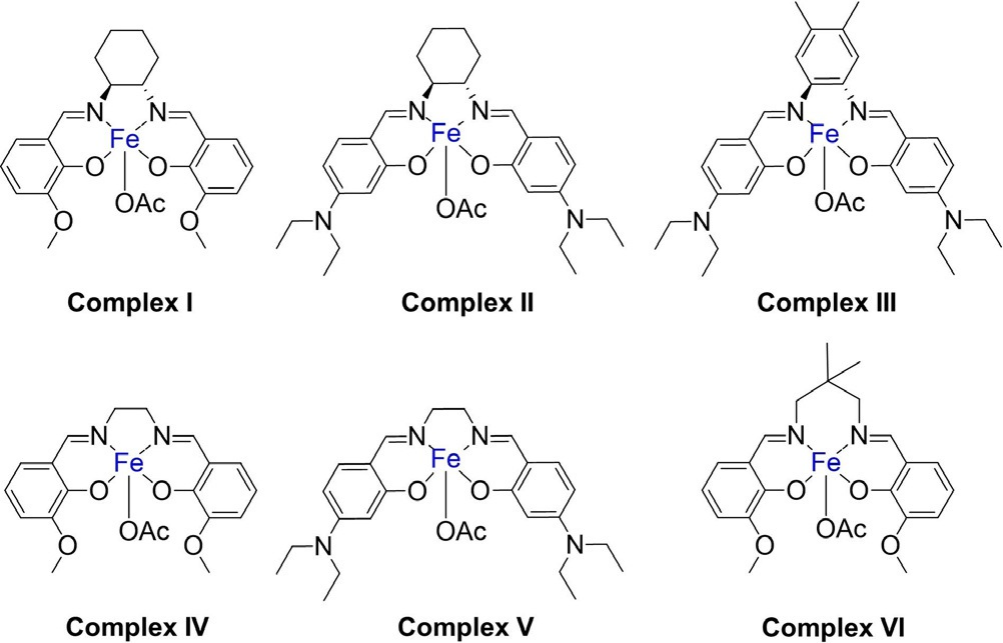
Different synthesized iron complexes used as metal source for the catalyst screening; OAc=acetate.

We were next interested in the broad applicability of our Fe‐based catalyst system in the reductive amination of carbonyl compounds to primary amines. All yields of primary amines are isolated yields for the corresponding hydrochloride salts. Aryl–alkyl ketones with various substituents were aminated in good‐to‐excellent yields. Ketones with electron‐donating substituents (e.g., methoxy, methyl) on the aromatic ring could be smoothly converted in isolated yields up to 96 % (Table 2, entries 2–4, 8, 11, 14).

**Table 2:** Reductive amination of ketones to primary amines.^[a]^

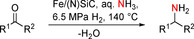			
Entry	Product		Yield^[b]^ [%]
1	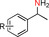	R=H	99
2		R=4‐CH_3_	96
3		R=3‐CH_3_	90
4		R=2‐CH_3_	82
5		R=4‐Cl	86
6		R=4‐Br	81
7		R=4‐F	83
8		R=4‐OCH_3_	87
9		R=4‐CF_3_	79
10		R=4‐SO_2_CH_3_	81
11		R=3,4‐di‐CH_3_	83
12	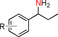	R=H	93
13		R=4‐CH_3_	91
14		R=4‐OCH_3_	82
15		R=4‐Cl	89
16	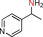		75^[c]^
17	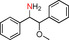		86^[c]^
18	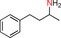		87
19	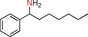		85
20	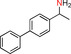		79^[c]^
21	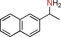		91

[a] Reaction conditions: Fe [10 mol %, 70 mg Fe/(N)SiC with 4.0 wt % Fe loading, 0.05 mmol Fe, 2.8 mg Fe], ketone (0.5 mmol), 140 °C, 20 h, 6.5 MPa H_2_, aq. NH_3_‐25 % (3.5 mL). [b] Isolated yields of the corresponding hydrochloride salts. [c] 150 °C.

The position of the substituent played a decisive role. *ortho*‐Substituted ketones were more difficult to aminate to the corresponding primary amines than the *meta*‐ and *para*‐substituted ones, most likely owing to the increasing steric hindrance (Table 2, entries 2–4). Electron‐withdrawing substituents, such as halogens and sulfonyl groups (Table 2, entry 10), were well tolerated. Therefore, different substituted chloro‐, fluoro‐, CF_3_‐, and bromo‐ketones were converted into their corresponding amines in good yields with only traces of dehalogenation (Table 2, entries 5–7, 9, 15). The reductive amination of a heteroaromatic ketone containing nitrogen was accomplished with a good yield of 75 % (Table 2, entry 16). Additionally, sterically demanding aryl–alkyl ketones, such as 2‐methoxy‐1,2‐diphenylethanamine, could be isolated in very good yield (Table 2, entry 17). Moreover, the necessity of the α‐carbon (C_α_) position of the aromatic ring next to the carbonyl group was investigated. The amination of 4‐phenylbutan‐2‐one showed that ketones with a phenyl ring at the γ‐carbon position could be transformed with an impressive isolated yield of 87 % (Table 2, entry 18). Aryl–alkyl ketones with a long alkyl chain could be converted with our synthesis protocol (Table 2, entry 19, C_*n*=7_), albeit with a slightly lower yield of 85 versus 93 % (Table 2, entry 12, C_*n*=3_). The amination of a naphthalene‐based ketone proceeded smoothly, whereas the biphenyl‐substituted ketone required higher temperatures for attractive conversion (Table 2, entries 20 and 21).

The transformation of benzylic aldehydes was performed under milder conditions; thus, the self‐coupling reactions were reduced but not completely suppressed. Therefore, yields were slightly lower than for ketones. Halogenated aldehydes, including bromides, chlorides, and fluorides, were converted with good yields >75 % (Table 3, entries 23–25). The transformation in the presence of an electron‐donating methyl substituent was also accomplished with good yield (Table 3, entry 26). Synthetically useful functionalities, such as 4,4,5,5‐tetramethyl‐1,3,2‐dioxaborolanyl or dioxol, could be tolerated, albeit under milder reaction conditions and with higher catalyst loadings (Table 3, entries 27 and 28).

**Table 3:** Reductive amination of aldehydes to primary amines.^[a]^

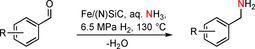			
Entry	Product		Yield^[b]^ [%]
22	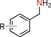	R=H	89
23		R=4‐Cl	79
24		R=4‐Br	75
25		R=4‐F	77
26		R=4‐CH_3_	81
27	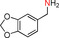		71^[c]^
28	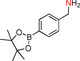		73^[c]^

[a] Reaction conditions: Fe [10 mol %, 70 mg Fe/(N)SiC with 4.0 wt % Fe loading, 0.05 mmol Fe, 2.8 mg Fe], aldehyde (0.5 mmol), 130 °C, 20 h, 6.5 MPa H_2_, aq. NH_3_‐25 % (3.5 mL). [b] Isolated yields of the corresponding hydrochloride salts. [c] 12 mol % Fe, 120 °C.

In addition, we tested purely aliphatic ketones in the reductive amination. Both aliphatic (Figure 3, entries 29–31) and cycloalkyl ketones (Figure 3, entries 32–34) of various length and ring sizes could be converted to the corresponding primary amines in yields up to 89 %. Finally, we were interested in introducing the −NH_2_ moiety in pharmaceuticals, biologically active, highly functionalized, and structurally complex molecules. The selective conversion of nabumetone, estrone, and stanolone to primary amines was achieved with yields over 91 % (Figure 3, entries 35–37). The C=C functionality of pregnenolone was tolerated, and the corresponding amine was isolated in 67 % yield (Figure 3, entry 38). Finally, the synthesis of central nervous system (CNS) stimulants was realized. Both 4‐ and 2‐methoxyamphetamine were obtained in good yields up to 88 % (Figure 3, entries 39 and 40).

**Figure 3:**
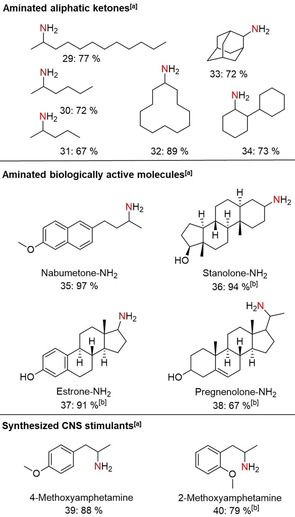
Reductive amination of purely aliphatic ketones and biologically active molecules. [a] Reaction conditions: Fe [10 mol %, 70 mg Fe/(N)SiC with 4.0 wt % Fe loading, 0.05 mmol Fe, 2.8 mg Fe], ketone (0.5 mmol), 140 °C, 20 h, 6.5 MPa H_2_, aq. NH_3_‐25 % (3.5 mL). Isolated yields of the corresponding hydrochloride salts. [b] 150 °C.

Regarding reusability and upscaling, finally, five consecutive runs without any loss of activity were performed (conditions for 99 % yield) to confirm the recyclability of our catalyst (Figure S12 in the Supporting Information). A scaled‐up reaction of acetophenone to 1‐phenylethanamine at gram scale was also performed with the same yield as the small‐scale experiment (see the Supporting Information).

In summary, we report a selective reusable iron‐based catalyst system for the synthesis of primary amines through reductive amination employing ammonia dissolved in water. Ketones, including purely aliphatic ones, aryl–alkyl, dialkyl, and heterocyclic, as well as aldehydes could be converted smoothly into their corresponding primary amines. In addition, the amination of pharmaceuticals, bioactive compounds, and natural products has been demonstrated. Many functional groups, such as hydroxy, methoxy, dioxol, sulfonyl, and boronate ester substituents, were tolerated. Our catalyst is easy to synthesize and handle. It is reusable, and upscaling has been demonstrated. The key is the use of a specific Fe complex and a N‐doped SiC material as a support to observe catalytic activity.

## Conflict of interest

*The authors declare no conflict of interest*.

## Supporting information

As a service to our authors and readers, this journal provides supporting information supplied by the authors. Such materials are peer reviewed and may be re‐organized for online delivery, but are not copy‐edited or typeset. Technical support issues arising from supporting information (other than missing files) should be addressed to the authors.

Supplementary
