# Supplementary material for: The Synthesis of Primary Amines through Reductive Amination Employing an Iron Catalyst
Source: ChemSusChem. 2020 May 26;13(12):3110–4. doi: 10.1002/cssc.202000856 (PMC7317915; doi:10.1002/cssc.202000856)
Supplement: Supplementary file 1 — Supplementary [file CSSC-13-3110-s001.pdf]

ChemSusChem

Supporting Information

The Synthesis of Primary Amines through Reductive Amination Employing an Iron Catalyst

Christoph Bäumler, Christof Bauer, and Rhett Kempe*[a]

Table of Contents

1 General considerations 3
2 Results and Discussion 5
2.1 Synthesis of the Fe salen complexes 5
2.2 Synthesis of the support material 7
2.3 Catalyst synthesis 8
2.4 Catalyst characterization 8
2.5 Catalytic studies 13
2.6 General catalytic procedures 16
2.7 Characterization of isolated products 17
2.8 NMR spectra 30
2.9 FTIR spectroscopy 76
2.10 Crystallographic data of complex I 80
3 References 84

1 General considerations

All air- and moisture sensitive reactions were performed under dry argon or nitrogen atmosphere using standard Schlenk and glove box techniques. All dried solvents were obtained from a solvent purification system (activated alumina cartridges) or purchased from Acros. Deuterated solvents were dried via molecular sieves. All chemicals were acquired from commercial sources with purity over 95 % and used without further purification. The precursor SMP 10 was purchased from Starfire Systems, New York, USA.

Hydrogen chemisorption measurements were performed by using a ChemBET Pulsar TPR/TPD instrument from Quantachrome.

Pyrolysis and reduction were performed under nitrogen or forming gas (90/10) atmosphere in a high temperature furnace (EHA 12/450B200, Carbolite) or in a ChemBET Pulsar TPR/TPD instrument from Quantachrome.

Transmission electron microscopy (TEM) was carried out by using a Variant LEO 9220 (200 kV) and a JEOL JEM 2200FS (200 kV) device. For the sample preparation the ceramic was suspended in chloroform and sonicated for 5 min. For analysis a LC200-Cu and a CF200-Cu grid were used.

Pore characterizations were carried out via nitrogen sorption measurements using a Nova2000e (Quantachrome) device. The pore size distribution was computed via DFT calculations [calculation model: N_2 at -196.15 °C on carbon (slit/cylindrical pore, NLDFT equilibrium model)]. The specific surface area was calculated by using p/p_0 values from 0.05-0.31 (BET).

Powder X-ray diffractograms (PXRD) were detected by a Panalytical Empyrean (Cu Kα radiation, 1.5405980 Å) instrument with a PIXcel1D-Medipix3 detector. The reference codes for comparison are 00-006-0696 for cubic iron and 01-075-1621 for graphite.

X-ray photoelectron spectroscopy (XPS) was performed using a PHI Versa Probe III instrument of Physical Electronics. As X-ray source a monochromatic Al K α with a spot size of 100 µm (24.5 W) was used. The kinetic pass energy of the photoelectrons was determined with a hemispheric analyzer (45°) set to pass energy of 26 eV for high-resolution spectra. Fourier transform infrared (FTIR) spectroscopy measurements were performed with a Cary 630 FTIR spectrometer (Agilent Technologies) over a range from 2000 cm^{-1} to 700 cm^{-1}.

Atom absorption spectroscopy (AAS) was performed with a Varian Spectr AA. 100 instrument.

Inductively coupled plasma optical emission spectrometry (ICP-OES) measurements were carried out on a Varian Vista-pro instrument. The digestion was performed in a Berghof Speed Wave 4 microwave. The carbon amount was determined by combustion analysis with a carbon analyzer Leco C-200 using SiC as standard and the nitrogen and oxygen content by hot gas

extraction with a Leco TC-436 N/O analyzer using TiN and WO3 as standards. The silicon amount was calculated.

SEM (scanning electron microscopy) and EDX (energy dispersive X-ray spectroscopy) were performed by using a Zeiss Ultra plus microscope with an acceleration voltage of 15 kV.

NMR measurements were carried out with a Varian INOVA 300 and 400 (300 MHz for ^{1}H, 75 MHz for ^{13}C; 400 MHz for ^{1}H, 100 MHz for ^{13}C) at 296 K. Chemical shifts are reported in ppm relative to the residual solvent signal (DMSO-d6: 7.26 ppm (1H), 77.16 ppm (^{13}C); DMSO-d6: 2.50 ppm (^{1}H), 39.51 ppm (^{13}C)), coupling constants (J) are reported in Hz.

All tested reactions were determined via GC and GC-MS analysis. GC analyses were carried out on an Agilent 6850 GC system equipped with an Optima 17 column (30 m x 0.32 mm x 0.25 μm). GC-MS analyses were carried out on an Agilent 7890A GC system equipped with a HP-5MS column (30 m x 0.32 mm x 0.25 μm) and a 5975C inert MSD.

The hydrogenation experiments were carried out with Parr Instrument stainless steel autoclaves N-MT5 300 mL equipped with heating mantles and temperature controller.

2 Results and Discussion

2.1 Synthesis of the Fe salen complexes

The Fe salen complex I was synthesized in a two-step procedure.

First, the 6,6'- ((1E,1'E) - ((1S,2S) – cyclohexane - 1,2 – diylbis (azanylylidene)) bis (methanylylidene)) bis (2-methoxyphenol) ligand was synthesized according to known literature procedure and characterized by NMR and IR and EA (calculated for $C_{22}H_{26}N_2O_4$: C, 69.09; H, 6.85; N, 7.32; found: C, 69.10; H, 7.04; N, 7.50).[1a]

Next, complex I was synthesized according to a known literature procedure[1b] and characterized by IR and EA (calculated for $C_{24}H_{27}FeN_2O_6$: C, 58.20; H, 5.49; N, 5.66; found: C, 57.17; H, 5.69; N, 5.41) and additionally characterized via X-ray single crystal structure analysis. 1.15 g (3 mmol) of the ligand was dissolved in 40 mL acetone while stirring. 521 mg (3mmol) Fe(II) acetate were added. Then, the solution was stirred under aerobic conditions for 24 h. The solvent was partially removed under reduced pressure and a purple precipitation was formed. The solution was filtered and washed with ethanol and acetone. Finally, a purple powder was obtained (1.5 g, 82 %).

($C_{24}H_{27}FeN_2O_6$, 495.12 g/mol)

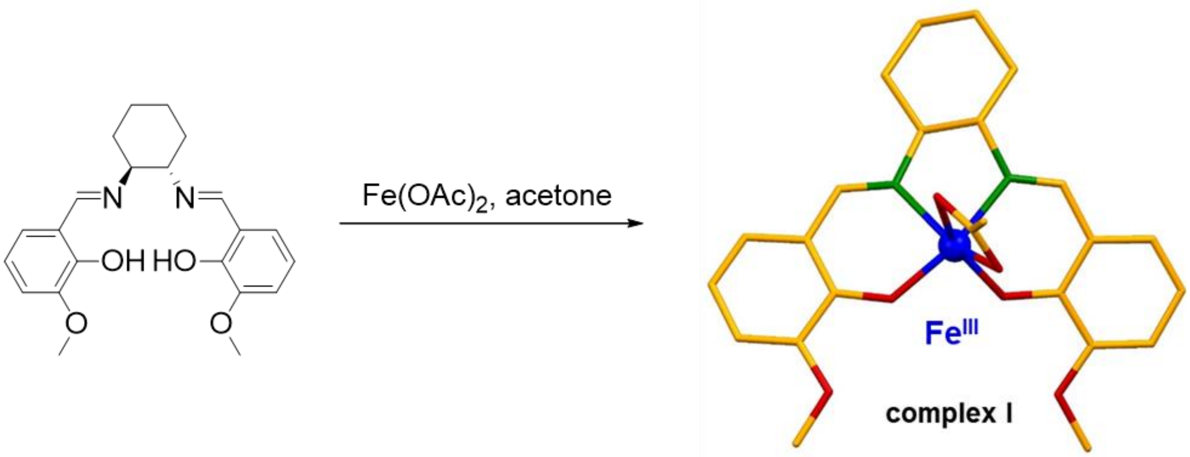

Figure S 1: Synthesis of the specific Fe complex I in the presence of air. The molecular structure of complex I was determined by X-ray single crystal structure analysis.

The synthesis of the complexes II-VI was accomplished in the same way as complex I. The ligands were characterized by EA and NMR analysis and the complexes by EA and IR analysis.

Table S 1: Synthesis of various Fe salen complexes.

Ligand II	Complex II
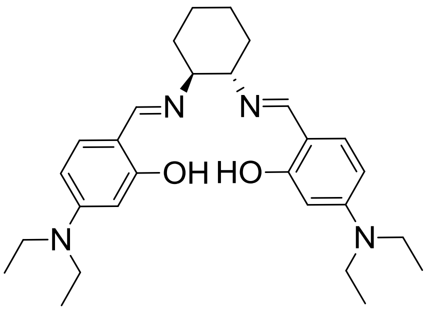	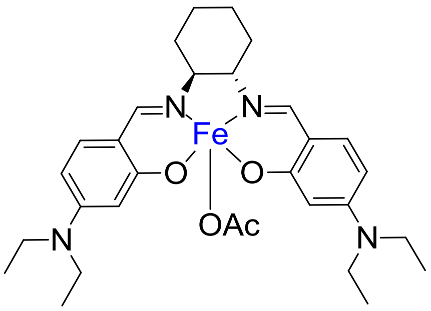
6,6'-((1E,1'E)-((1S,2S)-cyclohexane-1,2-diylbis(azanylylidene))bis(methanylylidene))bis(3-(diethylamino)phenol)<br>M: 464.32 g/mol (yellow-brown) $C_{28}H_{40}N_4O_2$	M: 577.25 g/mol (brown-purple) $C_{30}H_{41}FeN_4O_4$
EA: calc: C: 72.38; H: 8.68; N: 12.06<br>found: C: 71.99; H: 8.74; N: 11.84	EA: calc: C: 62.39; H: 7.16; N: 9.70<br>found: C: 61.98; H: 7.72; N: 9.85
Ligand III	Complex III
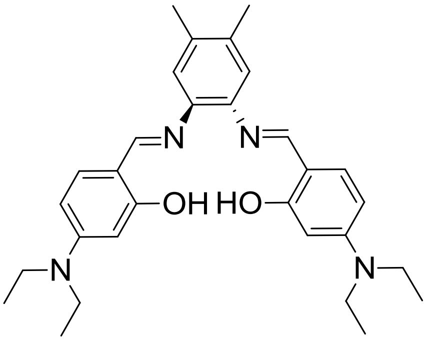	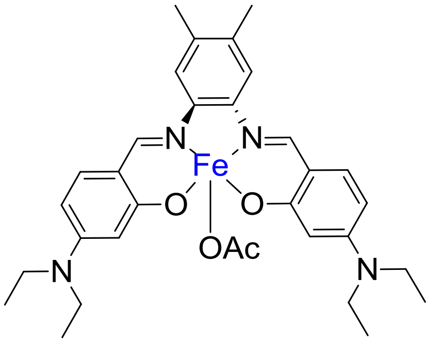
6,6'-((1E,1'E)-((4,5-dimethyl-1,2-phenylene)bis(azanylylidene))bis(methanylylidene))bis(3-(diethylamino)phenol)<br>M: 486.30 g/mol (orange-brown)/$C_{30}H_{38}N_4O_2$	M: 599.23 g/mol (purple)<br>$C_{32}H_{39}FeN_4O_4$
EA: calc: C: 74.04; H: 7.87; N: 11.51<br>found: C: 73.35; H: 8.15; N: 11.62	EA: calc: C: 64.11; H: 6.56; N: 9.35<br>found: C: 64.62; H: 7.26; N: 9.27
Ligand IV	Complex IV
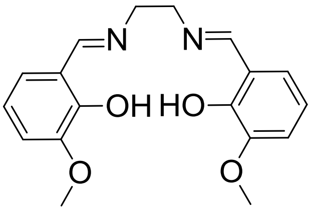	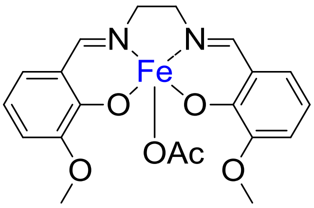
6,6'-((1E,1'E)-(ethane-1,2-diylbis(azanylylidene))bis(methanylylidene))bis(2-methoxyphenol)<br>M: 328.14 g/mol (yellow)/ $C_{18}H_{20}N_2O_4$	M: 441.07 g/mol (purple)<br>$C_{20}H_{21}FeN_2O_6$
EA: calc: C: 65.84; H: 6.14; N: 8.53<br>found: C: 65.89; H: 6.09; N: 8.26	EA: calc: C: 54.44; H: 4.80; N: 6.35<br>found: C: 54.00; H: 4.87; N: 6.06

Ligand V	Complex V
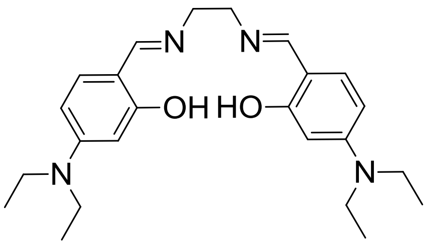	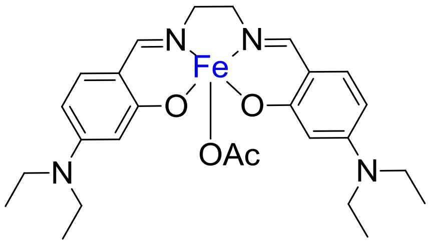
6,6'-((1E,1'E)-(ethane-1,2-diylbis(azanylylidene))bis(methanylylidene))bis(3-(diethylamino)phenol)<br>M: 410.27 g/mol (brown)// $C_{24}H_{34}N_4O_2$	M: 523.20 g/mol (dark purple) $C_{26}H_{35}FeN_4O_4$
EA: calc: C: 70.21; H: 8.35; N: 13.65<br>found: C: 69.63; H: 8.73; N: 13.61	EA: calc: C: 59.66; H: 6.74; N: 10.70<br>found: C: 59.46; H: 7.03; N: 10.94
Ligand VI	Complex VI
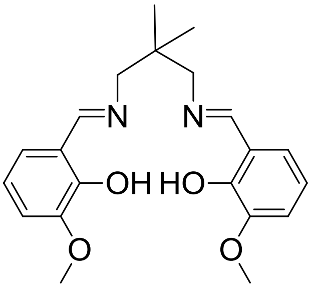	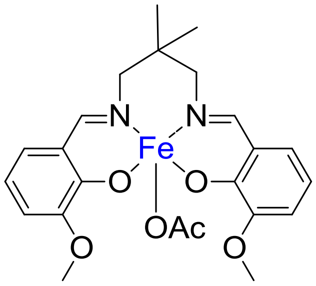
6,6'-((1E,1'E)-((2,2-dimethylpropane-1,3-diyl)bis(azanylylidene))bis(methanylylidene))bis(2-methoxyphenol)<br>M: 370.19 g/mol (yellow)// $C_{21}H_{26}N_2O_4$	M: 483.12 g/mol (purple)<br>$C_{23}H_{27}FeN_2O_6$
EA: calc: C: 68.09; H: 7.07; N: 7.56<br>found: C: 67.60; H: 7.14; N: 7.39	EA: calc: C: 57.16; H: 5.63; N: 5.80<br>found: C: 56.50; H: 5.62; N: 5.91

2.2 Synthesis of the support material

The (N)SiC material was prepared by modifying a known literature procedure.[2]

0.200 g SMP-10 (StarPCS^{TM}), 0.988 mL (0.800 g, 15.08 mmol) acrylonitrile (AN) and 0.075 g (0.46 mmol) azobisisobutyronitrile (AIBN) were dissolved in 4 mL dimethylformamide (DMF). After polymerization and crosslinking at 75 °C, the solvent was removed under reduced pressure. The obtained greenbody was pyrolyzed at 1000 °C under nitrogen atmosphere.

$$\text{RT} \xrightarrow[N_2]{5\ K/min} 200\ °C\ (0\ h) \xrightarrow{1\ K/min} 300\ °C\ (2h) \xrightarrow{5\ K/min} 1000\ °C\ (1h) \xrightarrow{20\ K/min} \text{RT}$$

The mass loss after pyrolysis was 45%. After ball milling for 20 minutes, 500 mg catalyst were washed by stirring in an aqueous solution of 6.7 mL NaOH (c = 1 mol/l) and 5 mL MeOH at 80 °C for 20 h under aerobic conditions. Afterwards the material was washed neutral and dried.

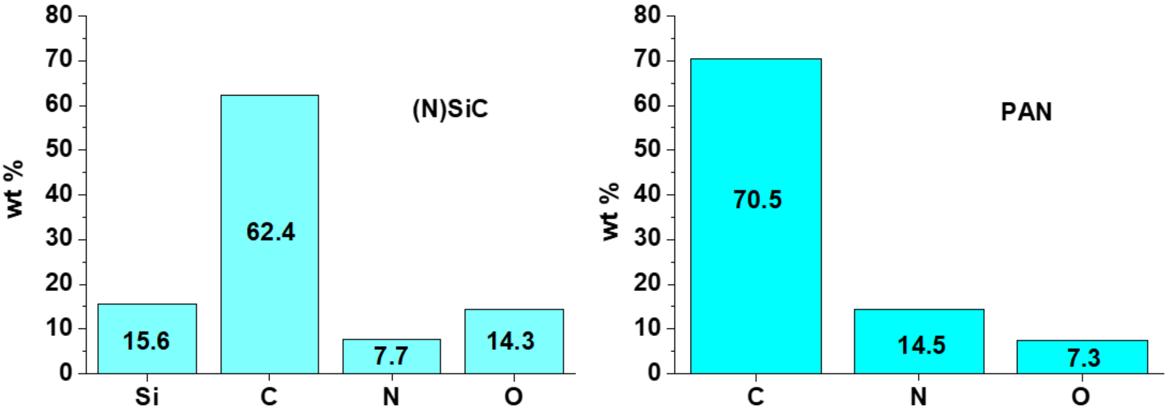

Figure S 2: Elemental analysis of the (N)SiC support and pyrolyzed polyacrylonitrile (PAN) as reference. As a result of the copolymerization of acrylonitrile and SiC, the nitrogen content is reduced. The high oxygen content is due to the polymerization in air atmosphere and the solvent DMF.

2.3 *Catalyst synthesis*

1.0 g (N)SiC were impregnated with 0.44 g **complex I** in 17 mL acetonitrile. After removal of the solvent, the sample was pyrolyzed under a nitrogen atmosphere at 750 °C followed by a treatment under a reductive atmosphere (N_2/H_2 90/10) at 550 °C.

$$\text{RT} \xrightarrow[N_2]{2\ K/min} 300\ °C\ (0.5\ h) \xrightarrow[N_2]{10\ K/min} 750\ °C\ (0.5\ h) \xrightarrow[N_2]{20\ K/min} 100\ °C \xrightarrow[H_2]{5\ K/min} 550\ °C\ (3\ h) \xrightarrow[N_2]{20\ K/min} \text{RT}$$

2.4 *Catalyst characterization*

AAS analysis of the catalyst:

Theoretical Fe content:	5.0 wt%
Measured Fe content:	4.0 wt%

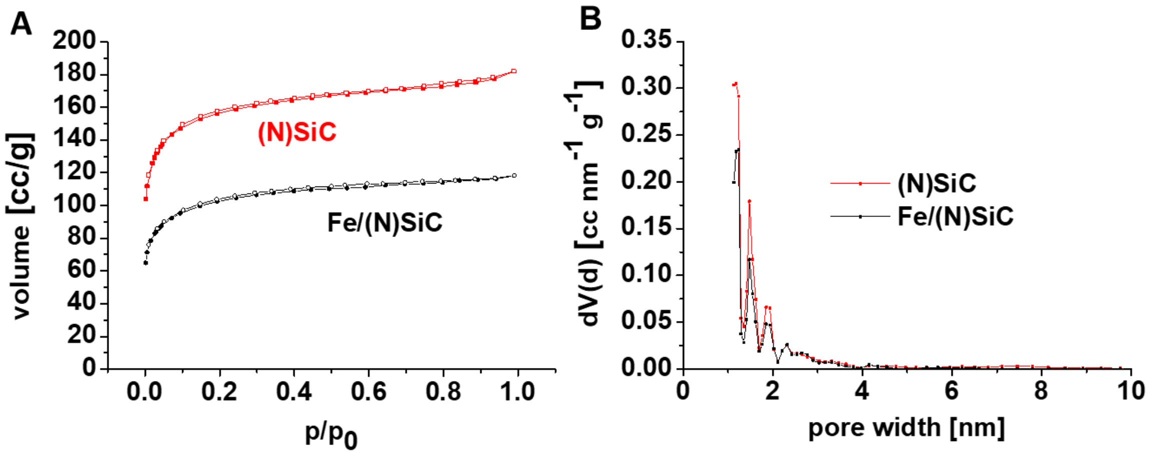

Figure S 3: Nitrogen physisorption measurements of the Fe catalyst and the (N)SiC support material. A: Isotherms of both materials. The course of the two isotherms indicates the presence of mainly micropores. A specific surface area of 485 m^2g^{-1} [(N)SiC] and 415 m^2g^{-1} (Fe catalyst) was calculated by the Brunauer-Emmett-Teller (BET) method. The lower surface area of the catalyst is due to the process of impregnation on the support material. **B:** The pore-size distributions show that micropores are responsible for the main part of the resulting surface areas [Calculation model: N_2 at 77 K on carbon (cylindr. pores, NLDFT equilibrium model)]. Both materials show a similar process in both isothermal and pore-size distributions.

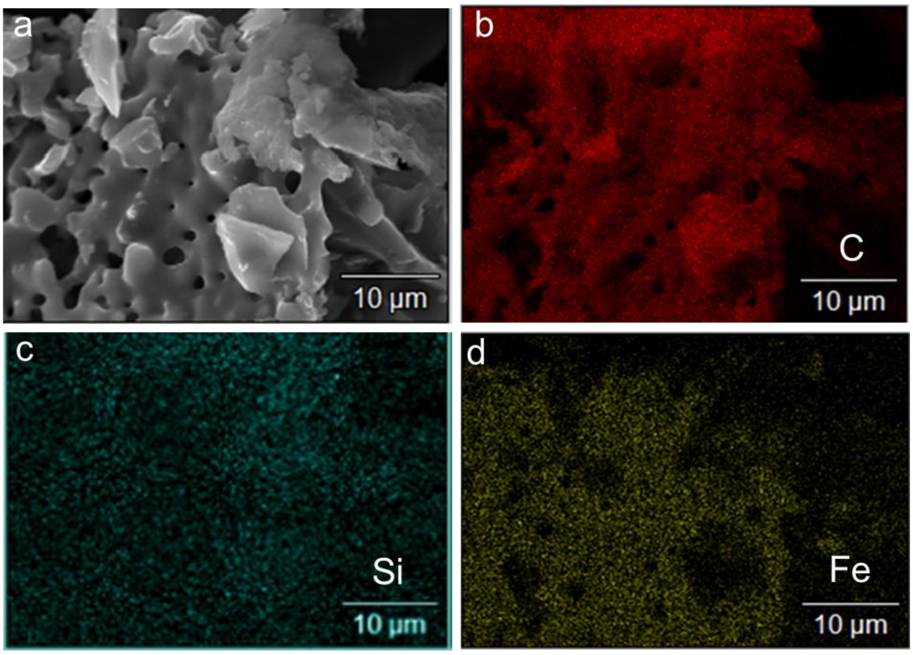

Figure S 4: Scanning electron microscopy (SEM, a) combined with energy dispersive X-ray (EDX, b-c) element maps. The iron nanoparticles have a homogeneous distribution on the surface. In addition, the individual elements of the carrier material are also very evenly distributed.

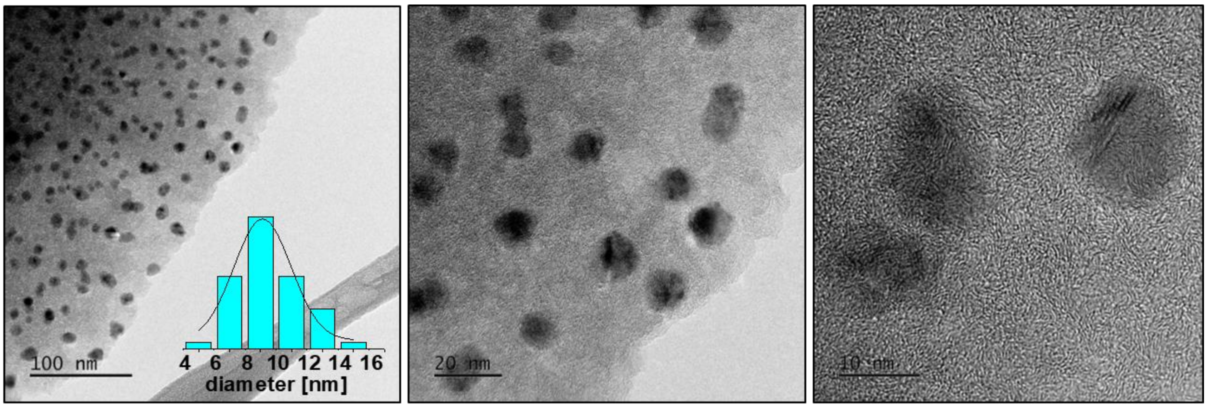

Figure S 5: Transmission electron microscopy (TEM) analysis of the Fe/(N)SiC catalyst surface. TEM analysis verifies a homogenous distribution of the Fe nanoparticles on the (N)SiC matrix. An average particle size of 10 nm could be determined.

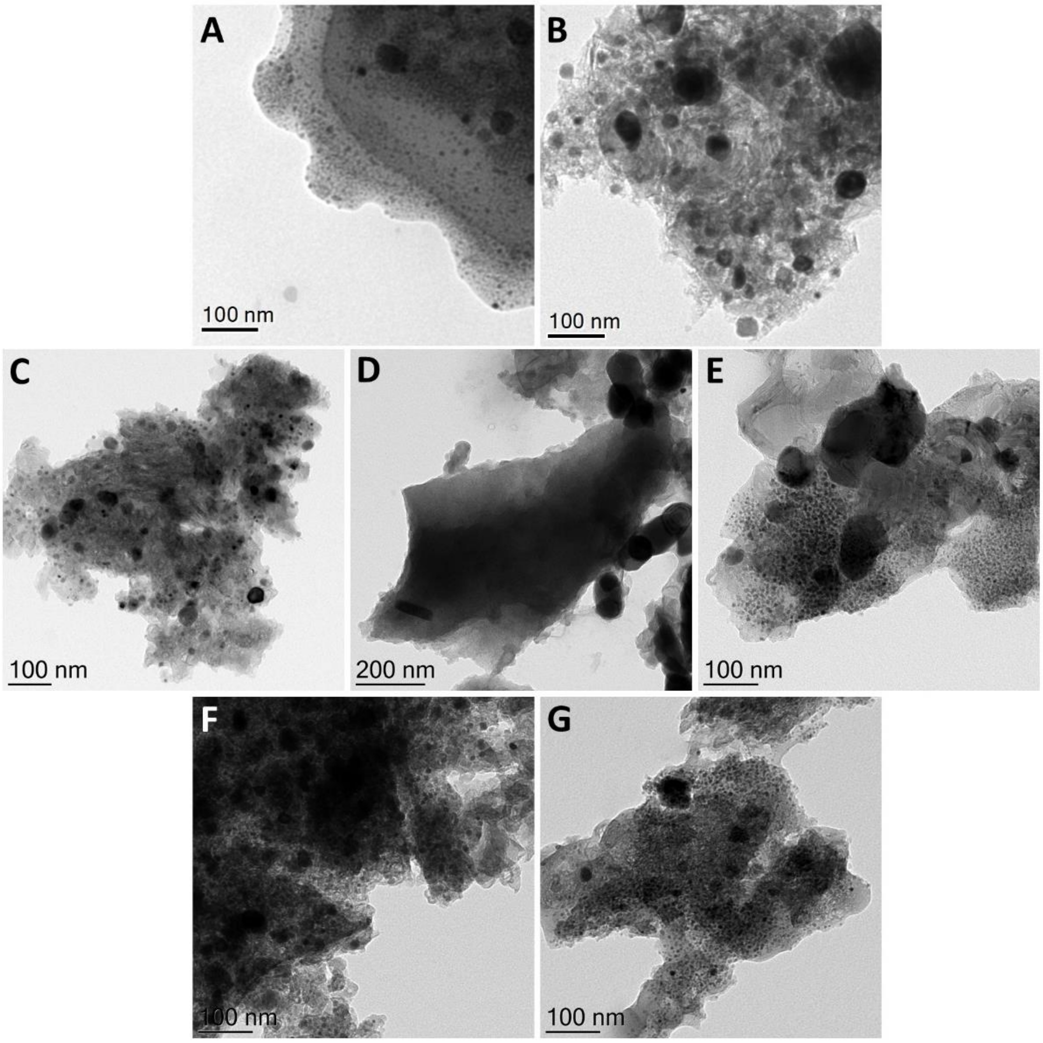

Figure S 6: TEM characterization of different catalysts used for the catalyst screening in the reductive amination of carbonyl compounds. Variation of the pyrolysis temperature for the synthesis of the Fe/NSiC catalyst with complex I and variation of the iron-salen complexes as metal source for the synthesis of various Fe/(N)SiC catalysts; A) 650 °C; B) 850 °C; C) Complex II; D) Complex III; E) Complex IV; F) Complex V; G) Complex VI.

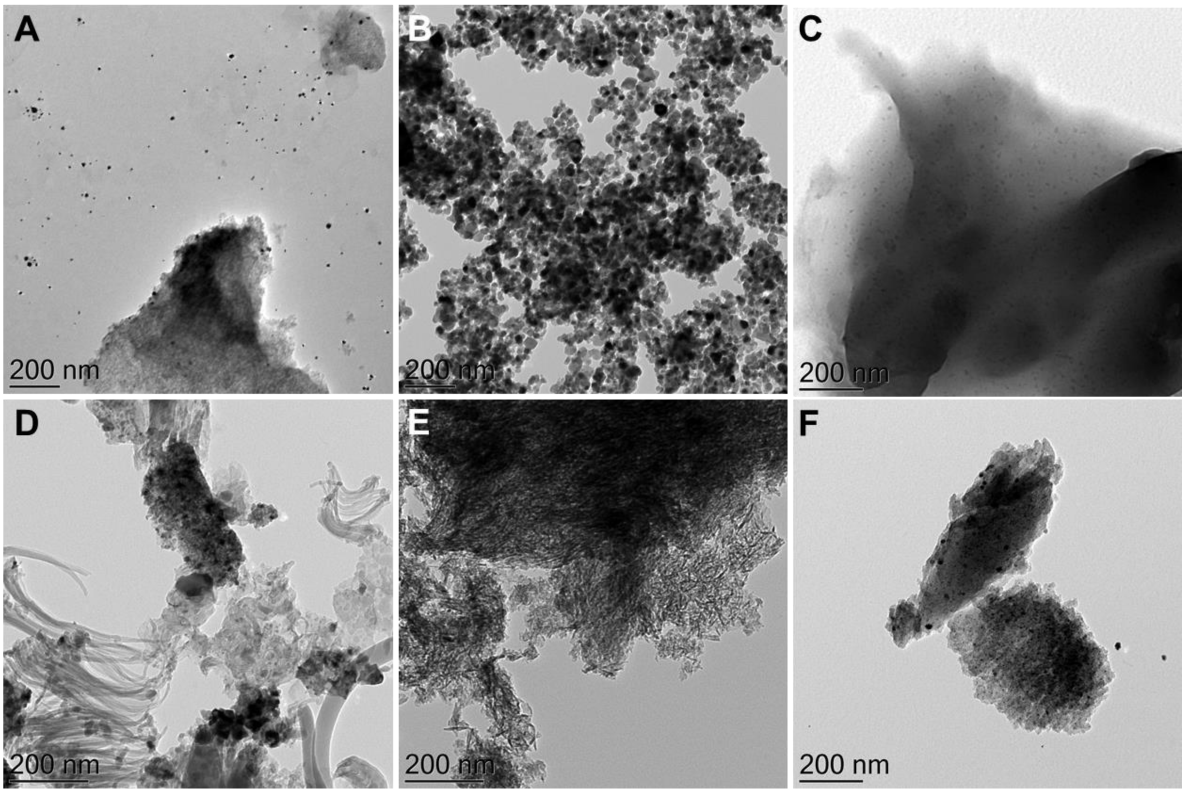

Figure S 7: TEM characterization of different catalysts used for the catalyst screening in the reductive amination of carbonyl compounds. Variation of the support material by using complex I as metal source; A) Fe/TiO_2; B) Fe/SiO_2; C) Fe/pyrolyzed PAN; D) Fe/CeO_2; E) Fe/Al_2O_3; F) Fe/active carbon.

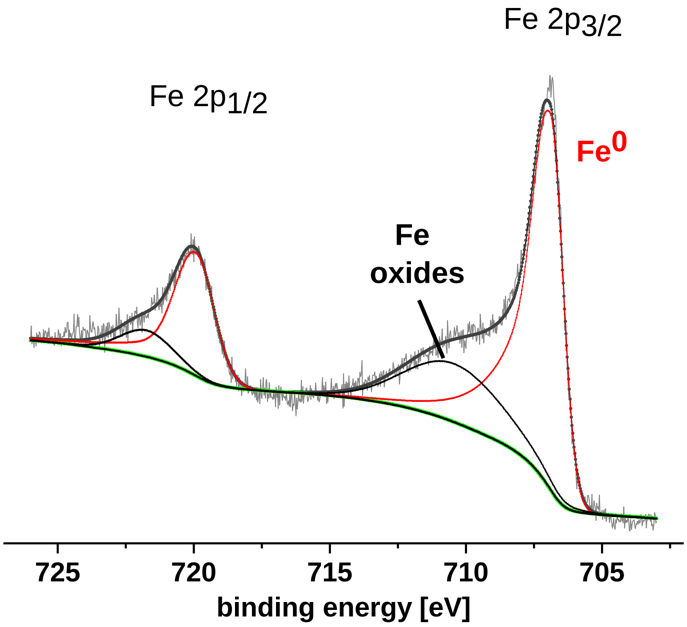

Figure S 8: XP spectrum of the catalyst. The Fe $2p_{3/2}$ signal can be divided in two signals with the binding energies of 707.0 and 711.0 eV. The presence of metallic Fe could be identified due to its sharp line shape and its binding energies of 707.0 eV. At higher binding energies small amounts of different oxides (e.g. FeO, Fe_2O_3) are found that cannot be distinguished in the analysis.

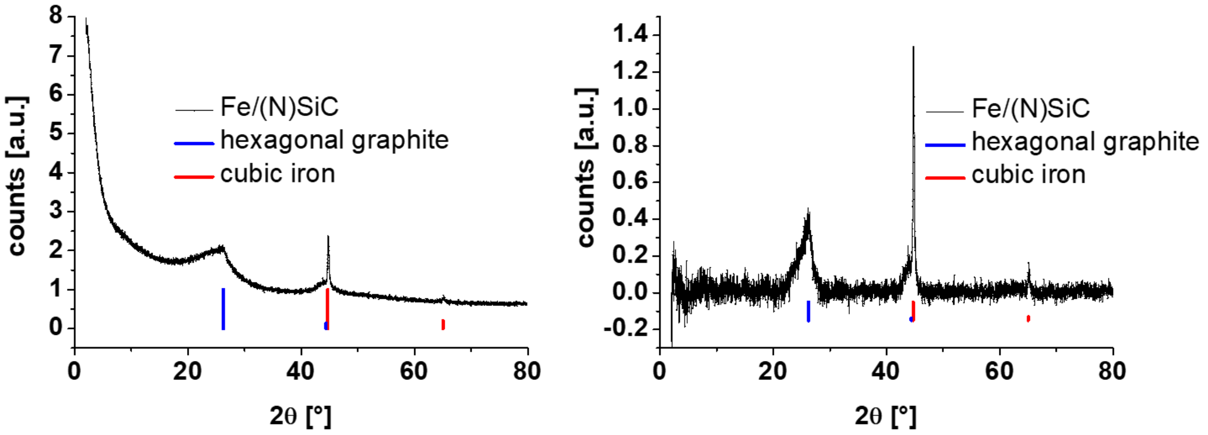

Figure S 9: Powder X-ray diffraction of the catalyst. The reflexes at the 2θ value of 44.67° and 65.02° can be assigned to the (110) and (200) reflexes of cubic iron (red). The reflexes at the 2θ value of 26.23° and 44.37° can be assigned to the (002) and (101) reflexes of hexagonal carbon (blue, graphite), what indicates a graphitic like structure what might be caused by the pyrolysis of polyacrylonitrile.

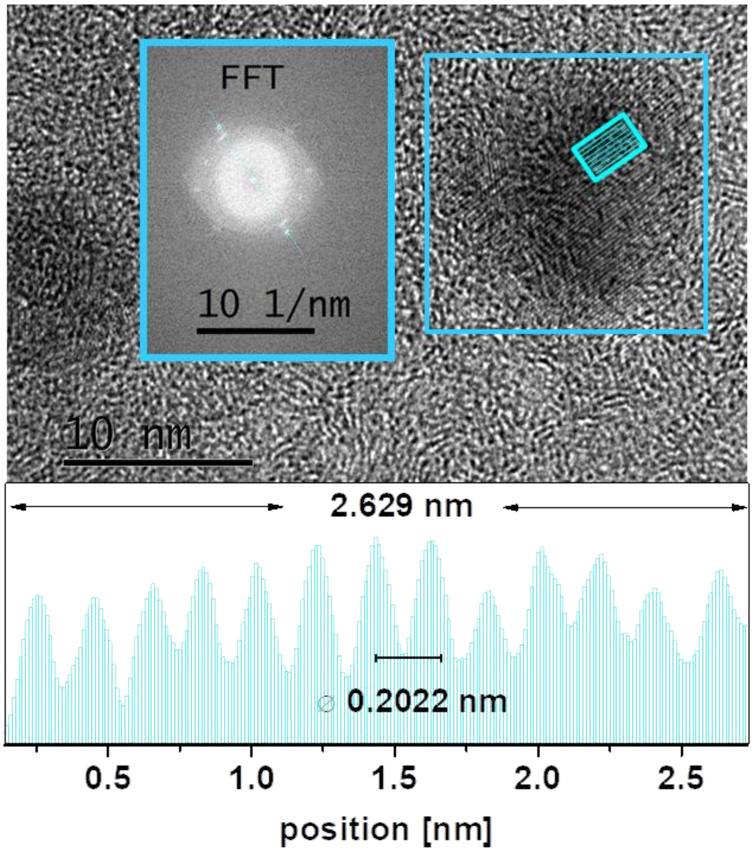

Figure S 10: HR-TEM analysis with focus on lattice spacing distances of the iron nanoparticles. Previous PXRD patterns showed matching reflections for cubic iron at 2θ = 44.67° with a lattice spacing of 0.2027 (110) nm. The FFT (Fast Fourier Transform) of the investigated nanoparticle, resulted in an averaged lattice plane distance of 0.2022 nm, which is in good agreement with the literature values.

2.5 Catalytic studies

Table S 2: Solvent screening for the reductive amination of acetophenone.

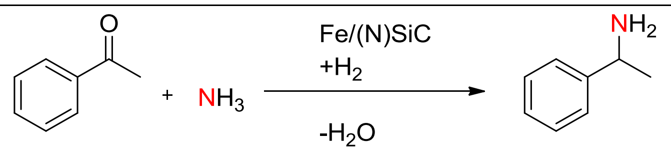

Solvent [2.0 mL]	Yield [%]
**H_2O**	**65**
Methanol	40
Ethanol	33
Toluene	15
Dioxane	32
Tetrahydrofuran	27

Reaction conditions: 10 mol% Fe (70 mg Fe/(N)SiC, 4.0 wt% Fe, 0.05 mmol Fe, 2.8 mg Fe), 0.5 mmol acetophenone, 140 °C, 20 h 6.5 MPa H_2, 1.5 mL aq. NH_3-25%. Yields were determined by GC using *n*-dodecane as an internal standard.

Table S 3: Screening of the NH_3 amount for the reductive amination of acetophenone.

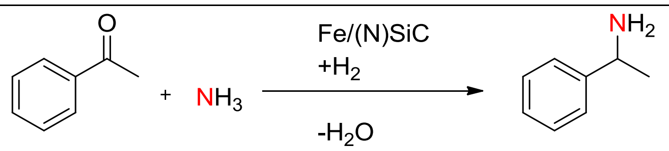

H_2O [mL]	aq. NH_3-25 % [mL]	Volume [mL]	Yield [%]
3.5	0.5 (6.7 mmol)	4.0	32
3.0	1.0 (13.4 mmol)	4.0	35
2.5	1.5 (20.0 mmol)	4.0	36
2.0	2.0 (26.7 mmol)	4.0	40
1.0	3.0 (40.1 mmol)	4.0	50
0	3.0 (40.1 mmol)	3.0	56
**0**	**3.5 (46.7 mmol)**	**3.5**	**60**
0	4.0 (53.4 mmol)	4.0	57
0	5.0 (66.8 mmol)	5.0	53

Reaction conditions: 10 mol% Fe (70 mg Fe/(N)SiC, 4.0 wt% Fe, 0.05 mmol Fe, 2.8 mg Fe), 0.5 mmol acetophenone, 130 °C, 20 h, 6.5 MPa H_2. Yields were determined by GC using *n*-dodecane as an internal standard.

Table S 4: Screening of the NH_3 amount for the reductive amination of benzaldehyde.

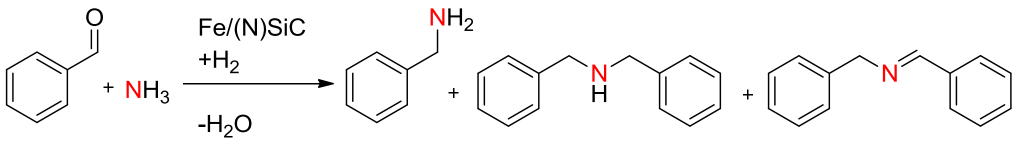

a b c

aq. NH_3-25 % [mL]	Volume [mL]	Yield[a] [%]		
		a	b	c
2.0 (26.7 mmol)	2.0	45	40	13
3.0 (40.1 mmol)	3.0	55	30	14
**3.5 (46.7 mmol)**	**3.5**	**63**	**18**	**18**
4.0 (53.4 mmol)	4.0	64	16	19
5.0 (66.8 mmol)	5.0	63	15	21

Reaction conditions: 8.6 mol% Fe (60 mg Fe/(N)SiC, 4.0 wt% Fe, 0.043 mmol Fe, 2.4 mg Fe), 0.5 mmol benzaldehyde, 125 °C, 20 h, 6.5 MPa H_2. Yields were determined by GC using *n*-dodecane as an internal standard.

Table S 5: Reductive amination of acetophenone / Optimization of the reaction parameters.

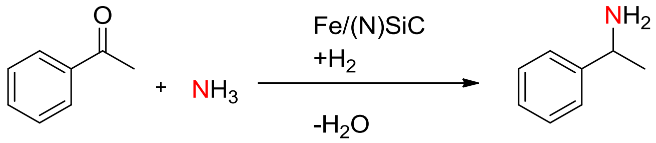

Entry	Catalyst loading [mol %]	Temperature [°C]	Pressure [MPa]	Time [h]	GC-yield [%]
1	8.6	120	6.5	20	32
2	10	120	6.5	20	39
3	8.6	130	6.5	20	46
4	10	130	6.5	20	60
5	8.6	140	5.0	20	54
6	10	140	5.0	20	76
7	8.6	140	6.5	20	72
8	10	140	6.5	2	11
9	10	140	6.5	4	27
10	10	140	6.5	16	87
**11**	**10**	**140**	**6.5**	**20**	**99**

Reaction conditions: 0.5 mmol acetophenone, 3.5 mL aq. NH_3-25%. Yields were determined by GC using *n*-dodecane as an internal standard.

Up-scaling of the reaction

In order to prove the usefulness of the catalyst, an up-scaling reaction to gram scale was carried out. Acetophenone was chosen as representative carbonyl substrate for this reaction. The optimized reaction conditions were scaled up for 10 mmol substrate (1.4 g Fe/(N)SiC, 10 mmol acetophenone, 140 °C, 20 h, 6.5 MPa H_2, 70 mL aq. NH_3-25%). The reaction was carried out in a 250 mL high pressure autoclave (Parr Instruments) in the same way as the 0.5 mmol reactions. Isolated yield of 1-phenylethanaminium chloride: 1.544 g, 98 %

Recycling studies

The recycling studies were accomplished with the optimized reaction conditions. Therefore, the Fe/(N)SiC catalyst was separated after each run via centrifugation, washed with ethyl acetate, dried under vacuum and used again without any reactivation. For determining the initial rate of each run, a recycling study with a run time of 4 h and the same reaction parameters were accomplished. The yields of 1-phenylethylamine were determined via GC using *n*-dodecane as an internal standard.

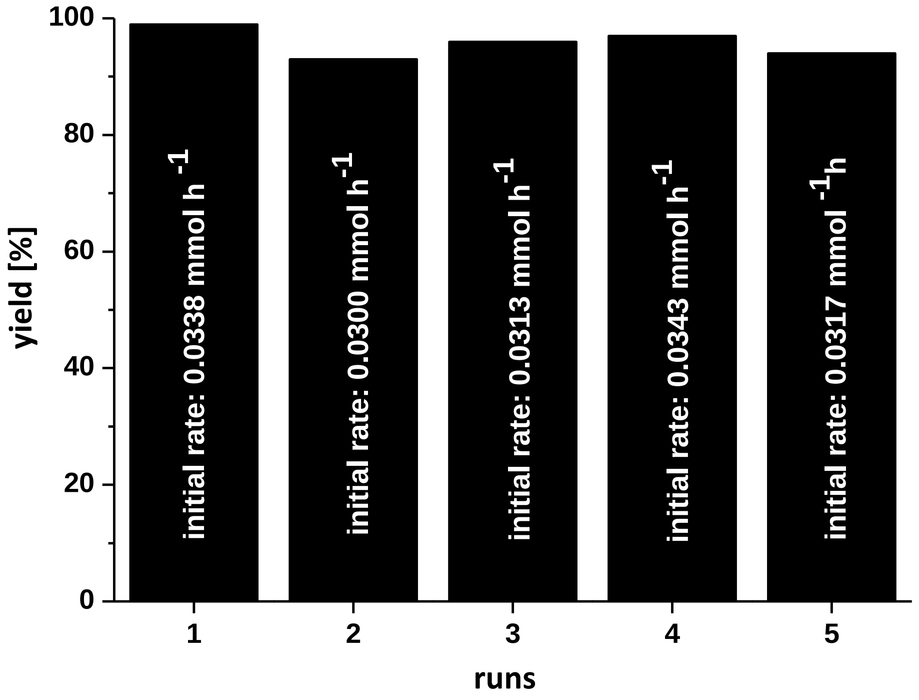

Figure S 11: Recycling study of the Fe/(N)SiC catalyst over 5 consecutive runs (conditions for 99 % yield), including initial rates. Reaction conditions: 10 mol% Fe (70 mg Fe/(N)SiC with 4.0 wt% Fe loading, 0.05 mmol Fe, 2.8 mg Fe), 0.5 mmol acetophenone, 140 °C, 20 h, 6.5 MPa H_2, 3.5 mL aq. NH_3-25%. Conditions for the initial rate 4 h instead of 20 h. Yields were determined by GC using *n*-dodecane as an internal standard.

2.6 General catalytic procedures

Reductive amination of aldehydes and ketones

A magnetic stirring bar, 0.5 mmol substrate, 3.5 mL aq. NH_3-25% and 10 mol % Fe catalyst (2.8 mg Fe, 0.05 mmol, 70 mg) were filled in a 5 mL reaction vial. The vial was placed in a high-pressure autoclave (Parr Instruments) and the autoclave was flushed three times with 2.0 MPa of hydrogen. The autoclave was pressured with 6.5 MPa of hydrogen and the reaction was stirred for 20 h at 130 °C for aldehydes and 140 °C for ketones. The autoclave was cooled to room temperature and the hydrogen pressure was released. Then, the solution was extracted with Methyl *tert*-butyl ether, dried over Na_2SO_4, filtered and the solvent was removed under reduced pressure. In case of primary amines based on aldehydes, column chromatography (silica, ethyl acetate-methanol mixture as eluent) was executed for purification and for isolating the primary amine. All amines were converted into their corresponding hydrochloride salts via HCl in Ether (0.7 mmol HCl) for further purification. The solvent was removed under reduced pressure and the salt was washed with ethyl acetate again twice. The obtained hydrochloride salts were further analyzed by ^{1}H and ^{13}C spectroscopy.

2.7 Characterization of isolated products

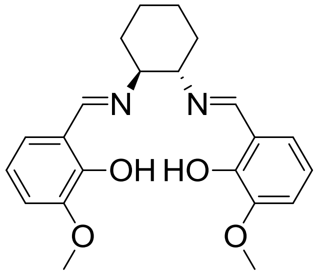

Ligand I:

6,6'-((1E,1'E)-((1S,2S)-cyclohexane-1,2-diylbis(azanylylidene))bis(methanylylidene)) bis(2-methoxyphenol)
[$C_{22}H_{26}N_2O_4$] 382.19 g/mol (M)

^{1}H NMR (400 MHz, $CDCl_3$, 296 K): δ = 13.85 (s, 2H), 8.24 (s, 2H), 6.85-6.83 (m, 2H), 6.79-6.77 (m, 2H), 6.73-6.69 (m, 2H), 3.85 (s, 6H), 3.32-3.29 (m, 2H), 1.95-1.85 (m, 4H), 1.74-1.66 (m, 2H), 1.52-1.44 (m, 2H) ppm.
^{13}C NMR (100 MHz, $CDCl_3$, 296 K): δ = 164.63, 151.42, 148.10, 123.02, 118.25, 117.76, 113.68, 72.28, 55.89, 32.90, 23.92 ppm.

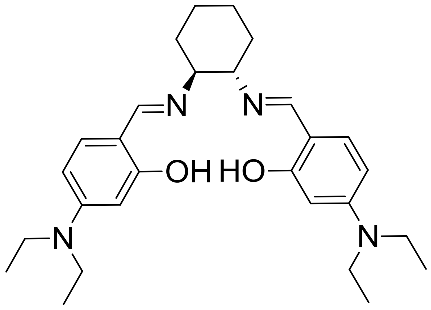

Ligand II:

6,6'-((1E,1'E)-((1S,2S)-cyclohexane-1,2-diylbis(azanylylidene))bis(methanylylidene))bis(3-(diethylamino)phenol)
[$C_{28}H_{40}N_4O_2$] 464.32 g/mol (M)

^{1}H NMR (400 MHz, $CDCl_3$, 296 K): δ = 7.94 (s, 2H), 6.90-6.88 (m, 2H), 6.08-6.03 (m, 4H), 3.35-3.30 (q, J = 7.03 Hz, 8H), 3.17-3.14 (m, 2H), 1.96-1.92 (m, 2H), 1.84-1.82 (m, 2H), 1.65-1.62 (m, 2H), 1.44-1.39 (m, 2H), 1.16-1.13 (t, J = 7.03 Hz, 12H) ppm.
^{13}C NMR (100 MHz, $CDCl_3$, 296 K): δ = 166.09, 162.63, 151.37, 108.16, 102.84, 98.06, 70.66, 44.38, 33.19, 24.28, 12.66 ppm.

Ligand III:

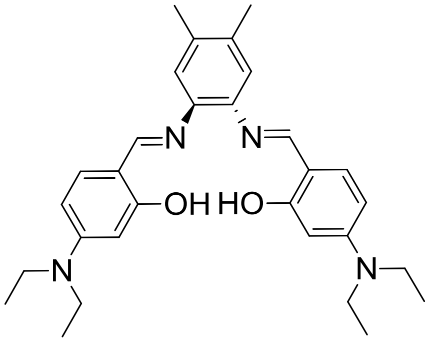

6,6'-((1E,1'E)-((4,5-dimethyl-1,2-phenylene)bis(azanylylidene)) bis(methanylylidene))bis(3-(diethylamino)phenol)
[$C_{30}H_{38}N_4O_2$] 486.30 g/mol (M)

^{1}H NMR (400 MHz, $CDCl_3$, 296 K): δ = 8.41 (s, 2H), 7.14-7.12 (m, 2H), 6.98 (s, 2H) 6.24-6.20 (m, 4H) 3.41-3.36 (q, J=7.03 Hz, 8H), 2.30 (s, 6H), 1.21-1.18 (t, J=7.03 Hz, 12H) ppm.
^{13}C NMR (100 MHz, $CDCl_3$, 296 K): δ = 164.77, 159.89, 151.69, 139.57, 134.45, 133.54, 120.17, 109.51, 103.47, 98.21, 44.52, 19.47, 12.72 ppm.

Ligand IV:

6,6'-((1E,1'E)-(ethane-1,2-diylbis(azanylylidene))bis(methanylylidene))bis(2-methoxyphenol)
[$C_{18}H_{20}N_2O_4$] 328.14 g/mol (M)

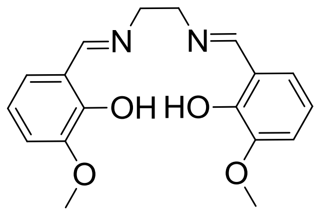

^{1}H NMR (400 MHz, $CDCl_3$, 296 K): δ = 13.55 (s, 2H), 8.32 (s, 2H), 6.90-6.75 (m, 6H), 3.94 (s, 4H), 3.88 (s, 6H) ppm.
^{13}C NMR (100 MHz, $CDCl_3$, 296 K): δ = 166.57, 151.34, 148.19, 123.07, 118.32, 117.96, 113.96, 59.37, 55.94 ppm.

Ligand V:

6,6'-((1E,1'E)-(ethane-1,2-diylbis(azanylylidene))bis(methanylylidene))bis(3-(diethylamino)phenol)
[$C_{24}H_{34}N_4O_2$] 410.27 g/mol (M)

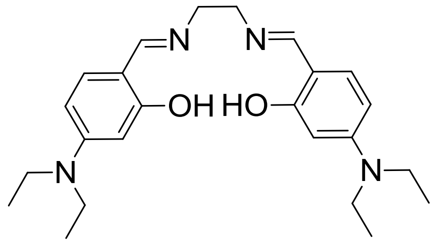

^{1}H NMR (400 MHz, $CDCl_3$, 296 K): δ = 8.02 (s, 2H), 6.98-6.95 (m, 2H), 6.14-6.09 (m, 4H) 3.75 (s, 4H), 3.38-3.33 (q, J=7.03 Hz, 6H), 1.19-1.15 (t, J=7.03 Hz, 12H) ppm.
^{13}C NMR (100 MHz, $CDCl_3$, 296 K): δ = 165.83, 164.27, 151.48, 132.95, 108.23, 102.99, 98.08, 58.09, 44.42, 12.68 ppm.

Ligand VI:

6,6'-((1E,1'E)-((2,2-dimethylpropane-1,3-diyl)bis(azanylylidene))bis(methanylylidene))bis(2-methoxyphenol)
[$C_{21}H_{26}N_2O_4$] 370.19 g/mol (M)

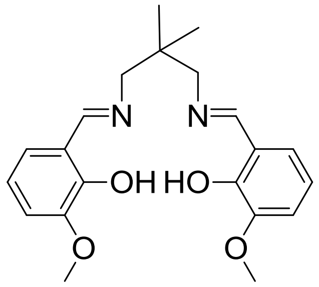

^{1}H NMR (400 MHz, $CDCl_3$, 296 K): δ = 8.32 (s, 2H), 6.94-6.87 (m, 4H), 6.82-6.78 (m, 2H), 3.91 (s, 6H), 3.49 (s, 4H), 1.07 (s, 6H) ppm.
^{13}C NMR (100 MHz, $CDCl_3$, 296 K): δ = 165.72, 152.00, 148.39, 122.84, 118.29, 117.81, 113.70, 67.19, 55.93, 36.08, 24.16 ppm.

1:

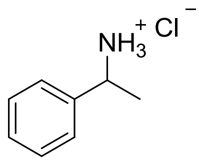

1-Phenylethanaminium chloride
[$C_8H_{12}ClN$] 157.64 g/mol (M)

^{1}H NMR (300 MHz, DMSO-d6, 296 K): δ = 8.78 (s, 3H), 7.57-7.55 (d, J = 7.03 Hz, 2H), 7.41-7.33 (m, 3H), 4.38-4.32 (q, J = 6.44 Hz, 1H), 1.54-1.51 (d, J = 6.44 Hz, 3H) ppm.
^{13}C NMR (75 MHz, DMSO-d6, 296 K): δ = 139.54, 128.63, 128.28, 126.92, 50.10, 20.93 ppm.

2:

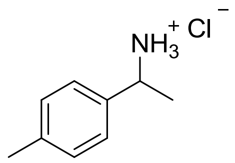

1-(p-Tolyl)ethanaminium chloride
[$C_9H_{14}ClN$] 171.67g/mol (M)

^{1}H NMR (300 MHz, DMSO-d6, 296 K): δ = 8.68 (s, 3H), 7.43-7.41 (d, J = 8.2 Hz, 2H), 7.21-7.19 (d, J = 7.81 Hz, 2H), 4.32-4.29 (m, 1H), 2.29 (s, 3H), 1.51-1.49 (d, J = 6.64 Hz, 3H) ppm.
^{13}C NMR (75 MHz, DMSO-d6, 296 K): δ = 137.95, 136.92, 129.49, 127.20, 50.16, 21.22, 21.10 ppm.

3:

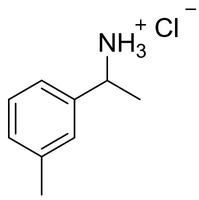

1-(m-Tolyl)ethanaminium chloride
[$C_9H_{14}ClN$] 171.67g/mol (M)

^{1}H NMR (300 MHz, DMSO-d6, 296 K): δ = 8.75 (s, 3H), 7.36-7.16 (m, 4H), 4.30 (m, 1H), 2.30 (s, 3H), 1.51 (m, 3H) ppm.
^{13}C NMR (75 MHz, DMSO-d6, 296 K): δ = 139.49, 137.74, 128.82, 128.55, 127.52, 123.92, 50.09, 21.07, 20.94 ppm.

4:

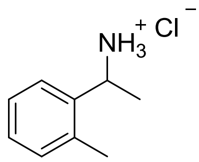

1-(o-Tolyl)ethanaminium chloride
[$C_9H_{14}ClN$] 171.67g/mol (M)

^{1}H NMR (300 MHz, DMSO-d6, 296 K): δ = 8.67 (s, 3H), 7.64-7.22 (m,4H), 4.50 (m, 1H), 2.34 (s, 3H), 1.49-1.48 (d, J = 6.25 Hz, 3H) ppm.
^{13}C NMR (75 MHz, DMSO-d6, 296 K): δ = 137.87, 135.04, 130.49, 128.01, 126.47, 125.45, 46.16, 20.37, 18.80 ppm.

5:

1-(4-Chlorophenyl)ethanaminium chloride
[$C_8H_{11}Cl_2N$] 192.02 g/mol (M)

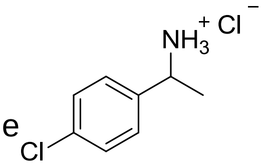

^{1}H NMR (300 MHz, DMSO-d6, 296 K): δ = 8.70 (s, 3H), 7.58-7.47 (m, 4H), 4.38-4.32 (m, 1H), 1.51 (m, 3H) ppm.
^{13}C NMR (75 MHz, DMSO-d6, 296 K): δ = 138.48, 132.91, 129.00, 128.57, 49.37, 20.68 ppm.

6:

1-(4-Bromophenyl)ethanaminium chloride
[$C_8H_{11}BrClN$] 236.54 g/mol (M)

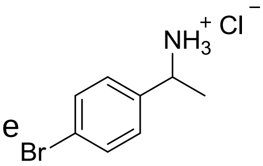

^{1}H NMR (300 MHz, DMSO-d6, 296 K): δ = 8.63 (s, 3H), 7.64-7.62(m, 2H), 7.51-7.48 (m, 2H), 4.39 (m, 1H), 1.50-1.49 (d, J = 6.63 Hz, 3H) ppm.
^{13}C NMR (75 MHz, DMSO-d6, 296 K): δ = 138.78, 131.56, 129.21, 121.57, 49.36, 20.57 ppm.

7:

1-(4-Fluorophenyl)ethanaminium chloride
[$C_8H_{11}ClFN$] 175.63 g/mol (M)

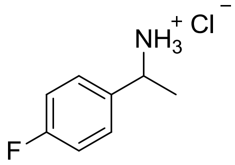

^{1}H NMR (300 MHz, DMSO-d6, 296 K): δ = 8.75 (s, 3H), 7.64-7.60 (m, 2H), 7.27-7.21 (m, 2H), 4.43-4.36 (q, J = 6.44 Hz, 1H), 1.52-1.50 (d, J = 6.44 Hz, 3H) ppm.
^{13}C NMR (75 MHz, DMSO-d6, 296 K): δ = 163.51, 160.28, 135.74, 129.31, 129.20, 115.54, 115.25, 49.34, 20.79 ppm.

8:

1-(4-Methoxyphenyl)ethanaminium chloride
[$C_9H_{14}ClNO$] 187.67 g/mol (M)

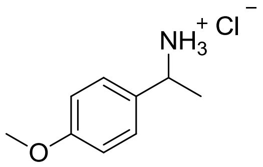

^{1}H NMR (300 MHz, DMSO-d6, 296 K): δ = 8.54 (s, 3H), 7.47-7.44 (m, 2H), 6.97-6.94 (m, 2H), 4.31-4.29 (m, 1H), 3.75 (s, 3H), 1.52-1.50 (d, J = 6.44 Hz, 3H) ppm.
^{13}C NMR (75 MHz, DMSO-d6, 296 K): δ = 159.26, 131.36, 128.33, 114.00, 55.26, 49.53, 20.76 ppm.

9:

1-(4-(Trifluoromethyl)phenyl)ethanaminium chloride
[$C_9H_{11}ClF_3N$] 225.64 g/mol (M)

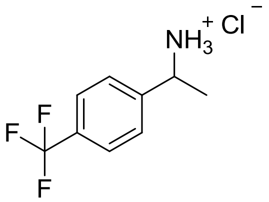

^{1}H NMR (300 MHz, DMSO-d6, 296 K): δ = 8.75 (s, 3H), 7.82-7.76 (m, 4H), 4.53-4.48 (m, 1H), 3.75 (s, 3H), 1.54-1.53 (d, J = 7.03 Hz, 3H) ppm.
^{13}C NMR (75 MHz, DMSO-d6, 296 K): δ = 144.00, 127.84, 125.60, 125.57, 125.46, 49.52, 20.62 ppm.

10:

1-(3,4-Dimethylphenyl)ethanaminium chloride
[$C_{10}H_{16}ClN$] 185.69 g/mol (M)

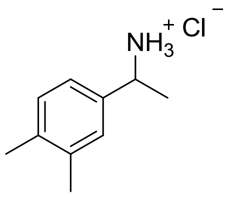

^{1}H NMR (300 MHz, DMSO-d6, 296 K): δ = 7.43 (s, 3H), 7.23-7.12 (m, 3H), 4.23-4.16 (m, 1H), 2.22-2.20 (d, J = 4.69 Hz, 6H), 1.43-1.41 (d, J = 7.03 Hz, 3H) ppm.
^{13}C NMR (75 MHz, DMSO-d6, 296 K): δ = 136.81, 136.40, 136.32, 129.64, 127.95, 124.13, 49.81, 20.82, 19.49, 19.08 ppm.

11:

1-(4-(Methylsulfonyl)phenyl)ethanaminium chloride
[$C_9H_{14}ClNO_2S$] 235.73 g/mol (M)

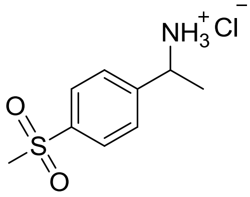

^{1}H NMR (300 MHz, DMSO-d6, 296 K): δ = 8.97 (s, 3H), 7.98-7.95 (d, J = 7.61 Hz, 2H), 7.87-7.84 (m, J = 7.61 Hz, 2H), 4.53-4.49 (m, 1H), 3.23 (s, 3H), 1.56-1.54 (d, J = 7.03 Hz, 3H) ppm.
^{13}C NMR (75 MHz, DMSO-d6, 296 K): δ = 145.19, 140.61, 128.05, 127.26, 49.58, 43.42, 20.74 ppm.

12:

1-Phenylpropan-1-aminium chloride
[$C_9H_{14}ClN$] 171.67 g/mol (M)

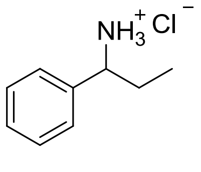

^{1}H NMR (300 MHz, DMSO-d6, 296 K): δ = 8.70 (s, 3H), 7.53-7.51 (m, 2H), 7.44-7.38 (m, 3H), 4.11-4.06 (m, 1H), 2.05-1.98 (m, 1H), 1.86-1.76 (m,1H), 0.76-0.71 (t, 3H) ppm.
^{13}C NMR (75 MHz, DMSO-d6, 296 K): δ = 137.80, 128.65, 128.44, 127.55, 55.87, 27.52, 10.05 ppm.

13:

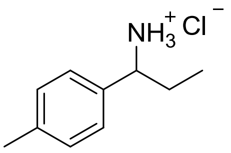

1-(p-Tolyl)propan-1-aminium chloride
[$C_{10}H_{16}ClN$] 185.69 g/mol (M)

^{1}H NMR (400 MHz, DMSO-d6, 296 K): δ = 8.68 (s, 3H), 7.40-7.38 (d, J = 8.2 Hz, 2H), 7.21-7.19 (d, J = 7.81 Hz, 2H), 4.02 (m, 1H), 2.29 (s, 3H), 2.05-2.00 (m, 1H), 1.81-1.77 (m,1H), 0.74-0.70 (t, J = 7.42 Hz, 3H) ppm.
^{13}C NMR (100 MHz, DMSO-d6, 296 K): δ = 137.66, 134.75, 129.11, 127.48, 55.63, 27.42, 20.73, 10.05 ppm.

14:

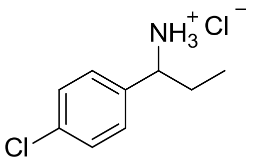

1-(4-Chlorophenyl)propan-1-aminium chloride
[$C_9H_{13}Cl_2N$] 206.11 g/mol (M)

^{1}H NMR (400 MHz, DMSO-d6, 296 K): δ = 8.80 (s, 3H), 7.58-7.56 (d, J = 8.59 Hz, 2H), 7.48-7.46 (d, J = 8.2 Hz, 2H), 4.13 (m, 1H), 2.06-2.00 (m, 1H), 1.82-1.78 (m,1H), 0.74-0.70 (t, J = 7.42 Hz, 3H) ppm.
^{13}C NMR (100 MHz, DMSO-d6, 296 K): δ = 136.80, 133.03, 129.63, 128.58, 55.12, 27.35, 9.98 ppm.

15:

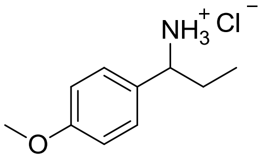

1-(4-Methoxyphenyl)propan-1-aminium chloride
[$C_{10}H_{16}ClNO$] 201.69 g/mol (M)

^{1}H NMR (400 MHz, DMSO-d6, 296 K): δ = 8.60 (s, 3H), 7.40-7.38 (d, J = 8.59 Hz, 2H), 6.92-6.90 (d, J = 8.2 Hz, 2H), 3.96 (m, 1H), 3.70 (s, 3H), 2.00-1.93 (m, 1H), 1.79-1.70 (m,1H), 0.68-0.64 (t, J = 7.42 Hz, 3H) ppm.
^{13}C NMR (100 MHz, DMSO-d6, 296 K): δ = 159.20, 129.60, 128.92, 113.93, 55.38, 55.17, 27.39, 10.10 ppm.

16:

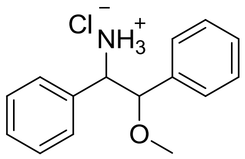

2-Methoxy-1,2-diphenylethanaminium chloride
[$C_{15}H_{18}ClNO$] 263.76 g/mol (M)

^{1}H NMR (300 MHz, DMSO-d6, 296 K): δ = 8.96 (s, 3H), 7.49-7.45 (m, 1H), 7.31-7.16 (m, 7H), 7.09-7.01 (m, 2H), 5.04-5.03 (d, J = 4.1 Hz, 0.8 H), 4.73-4.70 (d, J = 10.5 Hz, 0.2H), 4.49-4.48 (d, J = 4.7 Hz, 0.2H), 4.45-4.43 (d, J = 4.1 Hz, 0.8 H), 3.24-3.22 (d, J = 5.2 Hz, 3H) ppm.
^{13}C NMR (75 MHz, DMSO-d6, 296 K): δ = 137.13, 136.69, 133.39, 129.23, 128.98, 128.56, 128.39, 128.24, 128.13, 127.92, 127.67, 126.83, 84.14, 82.17, 59.28, 58.84, 57.04, 55.88 ppm.

17:

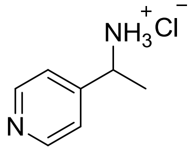

1-(Pyridin-4-yl)ethanaminium chloride
[$C_7H_{11}ClN_2$] 158.63 g/mol (M)

^{1}H NMR (300 MHz, DMSO-d6, 296 K): δ = 9.21 (s, 3H), 8.97-8.95 (m, 2H), 8.19-8.17 (m, 2H), 4.71-4.68 (m, 1H), 1.58-1.56 (d, J = 7.03 Hz, 3H) ppm.
^{13}C NMR (75 MHz, DMSO-d6, 296 K): δ = 156.77, 143.33, 124.73, 49.06, 20.15 ppm.

18:

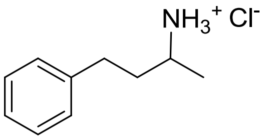

4-Phenylbutan-2-aminium chloride
[$C_{10}H_{16}ClN$] 185.69 g/mol (M)

^{1}H NMR (300 MHz, DMSO-d6, 296 K): δ = 8.29 (s, 3H), 7.30-7.16 (m, 5H), 3.11-3.10 (m, 1H), 2.68-2.62 (m, 2H), 1.96-1.92 (m, 1H), 1.76-1.73 (m, 1H), 1.25-1.23 (d, J = 6.24 Hz, 3H) ppm.
^{13}C NMR (75 MHz, DMSO-d6, 296 K): δ = 141.03, 128.40, 128.22, 125.96, 46.38, 35.84, 30.85, 18.01 ppm.

19:

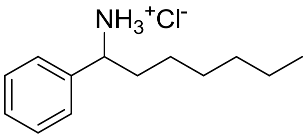

1-Phenylheptan-1-aminium chloride
[$C_{13}H_{22}ClN$] 227.77g/mol (M)

^{1}H NMR (300 MHz, DMSO-d6, 296 K): δ = 8.73 (s, 3H), 7.55-7.36 (m, 5H), 4.15-4.13 (m, 1H), 2.02-1.97 (m, 2H), 1.22-1.17 (m, 8H), 0.83-0.79 (t, J = 7.02 Hz, 3H) ppm.
^{13}C NMR (75 MHz, DMSO-d6, 296 K): δ = 138.06, 128.60, 128.37, 127.52, 54.52, 34.21, 30.97, 28.14, 24.98, 21.91, 13.86 ppm.

20:

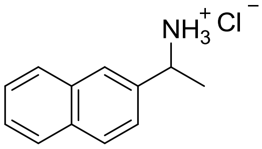

1-(Naphthalen-2-yl)ethanaminium chloride
[$C_{12}H_{14}ClN$] 207.70 g/mol (M)

^{1}H NMR (400 MHz, DMSO-d6, 296 K): δ = 8.81(s, 3H), 8.03-7.91 (m, 4H), 7.74-7.72 (m, 1H), 7.56-7.53 (m, 2H), 4.55-4.52 (m, 1H), 1.62-1.61 (d, J = 6.64 Hz, 3H) ppm.
^{13}C NMR (100 MHz, DMSO-d6, 296 K): δ = 136.91, 132.61, 128.36, 127.85, 127.62, 126.59, 126.48, 125.81, 124.68, 50.14, 20.73 ppm.

21:

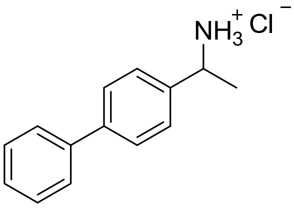

1-([1,1'-Biphenyl]-4-yl)ethanaminium chloride
[$C_{14}H_{16}ClN$] 233.74 g/mol (M)

^{1}H NMR (400 MHz, DMSO-d6, 296 K): δ = 8.77 (s, 3H), 7.72-7.63 (m, 5H), 7.48-7.45 (m, 3H), 7.39-7.35 (m, 1H), 4.45-4.39 (m, 1H), 1.58-1.56 (d, J = 6.64 Hz, 3H) ppm.
^{13}C NMR (100 MHz, DMSO-d6, 296 K): δ = 140.14, 139.56, 138.63, 128.99, 127.65, 127.54, 126.89, 126.71, 49.73, 20.79 ppm.

22:

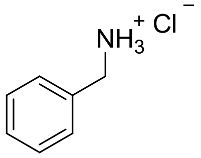

Phenylmethanaminium chloride
[$C_7H_{10}ClN$] 143.61 g/mol (M)

^{1}H NMR (300 MHz, DMSO-d6, 296 K): δ = 8.55 (s, 3H), 7.53-7.51 (m, 2H), 7.41-7.34 (m, 3H), 3.97 (s, 2H) ppm.
^{13}C NMR (75 MHz, DMSO-d6, 296 K): δ = 134.46, 128.94, 128.47, 128.24, 42.16 ppm.

23:

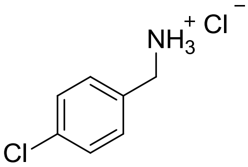

(4-Chlorophenyl)methanaminium chloride
[$C_7H_9Cl_2N$] 178.06 g/mol (M)

^{1}H NMR (300 MHz, DMSO-d6, 296 K): δ = 8.76 (s, 3H), 7.59-7.56 (m, 2H), 7.46-7.43 (m, 2H), 4.00 (s, 2H) ppm.
^{13}C NMR (75 MHz, DMSO-d6, 296 K): δ = 133.15, 133.09, 131.11, 128.45, 41.39 ppm.

24:

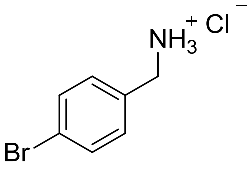

(4-Bromophenyl)methanaminium chloride
[C_7H_9BrClN] 222.51 g/mol (M)

^{1}H NMR (300 MHz, DMSO-d6, 296 K): δ = 8.71 (s, 3H), 7.61-7.58 (m, 2H), 7.50-7.48 (m, 2H), 3.98-3.96 (m, 2H) ppm.
^{13}C NMR (75 MHz, DMSO-d6, 296 K): δ = 133.58, 131.35, 121.66, 41.39 ppm.

25:

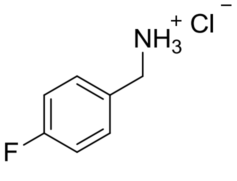

(4-Fluorophenyl)methanaminium chloride
[C_7H_9ClFN] 161.60 g/mol (M)

^{1}H NMR (300 MHz, DMSO-d6, 296 K): δ = 8.68 (s, 3H), 7.61-7.56 (m, 2H), 7.26-7.20 (m, 2H), 4.01-3.96 (m, 2H) ppm.
^{13}C NMR (75 MHz, DMSO-d6, 296 K): δ = 163.68, 160.45, 131.50, 130.46, 115.45, 115.18, 41.34 ppm.

26:

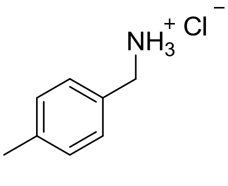

p-tolylmethanaminium chloride
[$C_8H_{12}ClN$] 157.64 g/mol (M)

^{1}H NMR (300 MHz, DMSO-d6, 296 K): δ = 9.05 (s, 3H), 7.57-7.54 (m, 2H), 7.27-7.25 (m, 2H), 4.85 (m, 2H), 2.34 (s, 3H) ppm.
^{13}C NMR (75 MHz, DMSO-d6, 296 K): δ = 163.68, 160.45, 131.50, 130.46, 115.45, 115.18, 41.34 ppm.

27:

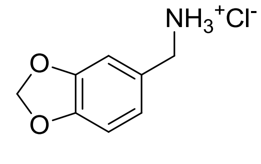

Benzo[d][1,3]dioxol-5-ylmethanaminium chloride
[$C_8H_{10}ClNO_2$] 187.62 g/mol (M)

^{1}H NMR (400 MHz, DMSO-d6, 296 K): δ = 8.61 (s, 3H), 7.16 (m, 1H), 6.99-6.96 (m, 1H), 6.92-6.90 (m, 1H), 6.01 (s, 2H), 3.89 (s, 2H) ppm.
^{13}C NMR (100 MHz, DMSO-d6, 296 K): δ = 147.25, 127.75, 122.95, 109.61, 108.22, 101.21, 41.94 ppm.

28:

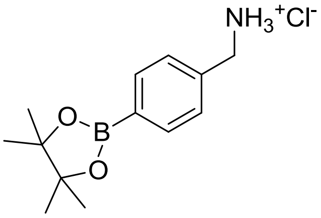

(4-(4,4,5,5-Tetramethyl-1,3,2-dioxaborolan-2-yl)phenyl)methanaminium chloride
[$C_{13}H_{21}BClNO_2$] 269,58 g/mol (M)

^{1}H NMR (400 MHz, DMSO-d6, 296 K): δ = 8.69 (s, 3H), 7.69-7.67 (d, J = 7.81 Hz, 2H), 7.52-7.51 (d, J = 7.81 Hz, 2H), 4.01 (s, 2H), 1.28 (s, 12H) ppm.
^{13}C NMR (100 MHz, DMSO-d6, 296 K): δ = 137.34, 134.49, 128.34, 83.74, 42.00, 24.66 ppm.

29:

Dodecan-2-aminium chloride
[$C_{12}H_8ClN$] 221.81 g/mol (M)

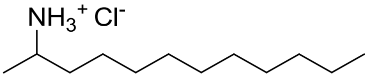

^{1}H NMR (300 MHz, DMSO-d6, 296 K): δ = 8.18 (s, 3H), 3.08-3.05 (m, 1H), 1.23-1.16 (m, 18H), 0.85-0.82 (m, 3H) ppm.
^{13}C NMR (75 MHz, DMSO-d6, 296 K): δ = 46.71, 34.02, 31.32, 29.02, 28.90, 28.83, 28.75, 28.73, 24.83, 22.12, 17.99, 13.91 ppm.

30:

Octan-2-aminium chloride
[$C_8H_{20}ClN$] 165.70 g/mol (M)

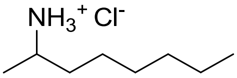

^{1}H NMR (300 MHz, DMSO-d6, 296 K): δ = 8.19 (s, 3H), 3.09-3.04 (m, 1H), 1.64-1.58 (m, 1H), 1.43-1.36 (m, 1H), 1.17-1.16 (d, J = 6.64 Hz, 3H), 0.86-0.83 (m, 3H) ppm.
^{13}C NMR (75 MHz, DMSO-d6, 296 K): δ = 47.14, 34.41, 31.48, 28.86, 25.16, 22.40, 18.40, 14.32 ppm.

31:

Pentan-2-aminium chloride
[$C_5H_{14}ClN$] 123.62 g/mol (M)

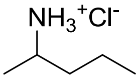

^{1}H NMR (300 MHz, DMSO-d6, 296 K): δ = 8.19 (s, 3H), 3.11-3.06 (m, 1H), 1.62-1.58 (m, 1H), 1.39-1.29 (m, 3H) 1.17-1.16 (d, J = 6.64 Hz, 3H), 0.87-0.83 (t, J = 7.03 Hz, 3H) ppm.
^{13}C NMR (75 MHz, DMSO-d6, 296 K): δ = 46.51, 36.18, 18.15, 18.03, 13.72 ppm.

32:

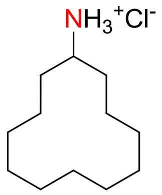

Cyclododecanaminium chloride
[$C_{22}H_{38}ClN$] 219.79 g/mol (M)

^{1}H NMR (300 MHz, DMSO-d6, 296 K): δ = 8.18 (s, 3H), 3.08-3.05 (m, 1H), 1.23-1.16 (m, 18H), 0.85-0.82 (m, 3H) ppm.
^{13}C NMR (75 MHz, DMSO-d6, 296 K): δ = 46.71, 34.02, 31.32, 29.02, 28.90, 28.83, 28.75, 28.73, 24.83, 22.12, 17.99, 13.91 ppm.

33:

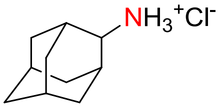

Adamantan-2-aminium chloride
[$C_{10}H_{18}ClN$] 187.71 g/mol (M)

^{1}H NMR (300 MHz, DMSO-d6, 296 K): δ = 8.37 (s, 3H), 3.24 (m, 1H), 2.32 (m, 1H), 2.08-2.01 (m, 3H) 1.82-1.68 (m, 8H), 1.53-1.50 (m, 2H) ppm.
^{13}C NMR (75 MHz, DMSO-d6, 296 K): δ = 54.66, 36.86, 36.15, 29.93, 29.49, 26.43, 26.34 ppm.

34:

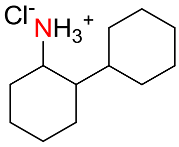

[1,1'-bi(cyclohexan)]-2-aminium chloride
[$C_{12}H_{24}ClN$] 217.78 g/mol (M)

^{1}H NMR (300 MHz, DMSO-d6, 296 K): δ = 8.06 (s, 3H), 3.46 (m, 1H), 2.22-0.74 (m, 20H) ppm.
^{13}C NMR (75 MHz, DMSO-d6, 296 K): δ = 55.26, 47.27, 44.00, 41.37, 35.59, 35.51, 30.84, 30.05, 29.28, 28.77, 28.65, 27.38, 26.07, 25.90, 25.82, 23.65, 22.13, 18.83 ppm.

35:

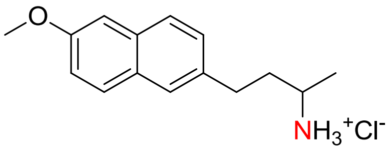

Nabutmetone- $NH_3^+Cl^-$
[$C_{15}H_{20}ClNO$] 265.78 g/mol (M)

^{1}H NMR (300 MHz, DMSO-d6, 296 K): δ = 8.22 (s, 3H), 7.78-7.66 (m, 3H), 7.38-7.29 (m, 2H), 7.17-7.13 (m, 1H), 3.87 (s, 3H), 3.16 (m, 1H), 2.86-2.77 (m, 2H), 2.12-1.77 (m, 2H), 1.29-1.27 (d, J = 6.44 Hz, 3H) ppm.
^{13}C NMR (75 MHz, DMSO-d6, 296 K): δ = 156.83, 136.06, 132.81, 128.79, 128.56, 127.55, 126.86, 125.95, 118.58, 105.75, 55.15, 46.42, 35.80, 30.80, 18.09 ppm.

36:

Stanolone-$NH_3^+Cl^-$
[$C_{19}H_{34}ClNO$] 327.93 g/mol (M)

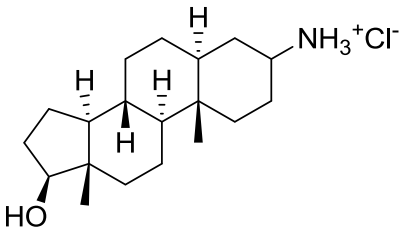

^{1}H NMR (300 MHz, DMSO-d6, 296 K): δ = 8.08 (s, 3H (2 H)), 4.44-4.43 (m, 1H), 3.45-3.41 (m, 1H), 2.93 (m,1H), 1.79-0.76 (m, 25H), 0.61 (s, 3H) ppm.
^{13}C NMR (75 MHz, DMSO-d6, 296 K): δ = 80.01, 53.62, 50.56, 49.43, 44.12, 42.55, 36.58, 36.02, 35.10, 32.32, 31.13, 29.84, 27.96, 25.82, 23.06, 20.33, 11.82, 11.33 ppm.

37:

Estrone-$NH_3^+Cl^-$
[$C_{18}H_{26}ClNO$] 307.86 g/mol (M)

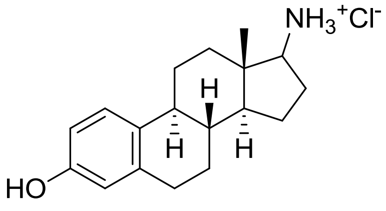

^{1}H NMR (300 MHz, DMSO-d6, 296 K): δ = 9.02 (s, 3H (1.4H)), 7.69-7.67 (m, 1H), 7.05-7.03 (m, 1H), 6.53-6.46 (m,2H), 4.20 (m, 1H), 2.74-2.73 (m, 2H), 2.41-2.29 (m, 2H), 2.08-1.93 (m, 4h), 1.75-1.72 (m, 1H), 1.54-1.43 (m, 2H), 1.37-1.22 (m, 4H), 0.81 (s, 3H) ppm.
^{13}C NMR (75 MHz, DMSO-d6, 296 K): δ = 155.51, 137.49, 130.37, 126.54, 115.43, 113.28, 59.90, 51.25, 50.06, 47.83, 43.94, 42.49, 38.68, 38.45, 35.87, 31.84, 29.54, 26.62, 26.04, 23.54, 21.62, 14.02, 12.08 ppm.

38:

Pregnenolone-$NH_3^+Cl^-$
[$C_{22}H_{38}ClNO$] 369.00 g/mol (M)

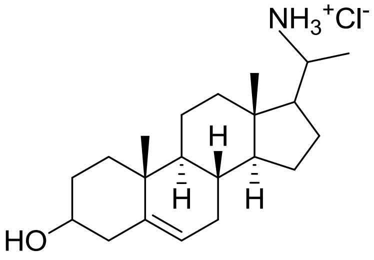

^{1}H NMR (300 MHz, DMSO-d6, 296 K): δ = 7.96 (s, 3H), 5.26 (m, 1H), 3.27-3.22 (m, 1H), 3.10-3.03 (m,1H), 2.16-0.64 (m, 30H) ppm.
^{13}C NMR (75 MHz, DMSO-d6, 296 K): δ = 141.28, 120.27, 69.97, 55.84, 53.86, 50.11, 49.47, 49.39, 48.20, 42.20, 41.68, 41.38, 38.38, 36.93, 36.06, 31.40, 31.26, 26.23, 25.49, 23.76, 23.55, 20.44, 19.15, 18.89, 18.77, 11.63, 11.49 ppm.

39:

1-(4-methoxyphenyl)propan-2-aminium chloride
[$C_{10}H_{16}ClNO$] 201.69 g/mol (M)

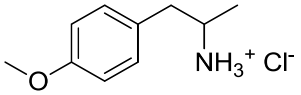

^{1}H NMR (300 MHz, DMSO-d6, 296 K): δ = 8.26 (s, 3H), 7.16-7.14 (m, 2H), 6.89-6.87 (m, 2H), 3.72 (s, 3H), 3.30 (m, 1H), 3.02-2.98 (m, 1H), 2.62-2.57 (m, 1H), 1.10-1.09 (d, J = 6.25 Hz, 3H) ppm.
^{13}C NMR (75 MHz, DMSO-d6, 296 K): δ = 158.06, 130.26, 128.67, 113.96, 55.02, 48.18, 39.14, 17.38 ppm.

40:

1-(2-methoxyphenyl)propan-2-aminium chloride
[$C_{10}H_{16}ClNO$] 201.69 g/mol (M)

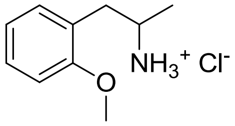

1-(2-methoxyphenyl)propan-2-aminium chloride

^{1}H NMR (300 MHz, DMSO-d6, 296 K): δ = 8.07 (s, 3H), 7.28-7.24 (m, 1H), 7.17-7.15 (m, 1H), 7.01-6.99 (m, 1H), 6.93-6.89 (m, 1H), 3.79 (s, 3H), 3.40-3.39 (m, 1H), 2.99-2.94 (m, 1H), 2.71-2.66 (m, 1H), 1.09-1.07 (d, J = 6.64 Hz, 3H) ppm.
^{13}C NMR (75 MHz, DMSO-d6, 296 K): δ = 157.28, 130.93, 128.43, 124.48, 120.40, 110.94, 55.34, 46.71, 34.92, 17.75 ppm.

2.8 NMR spectra

Ligand I:

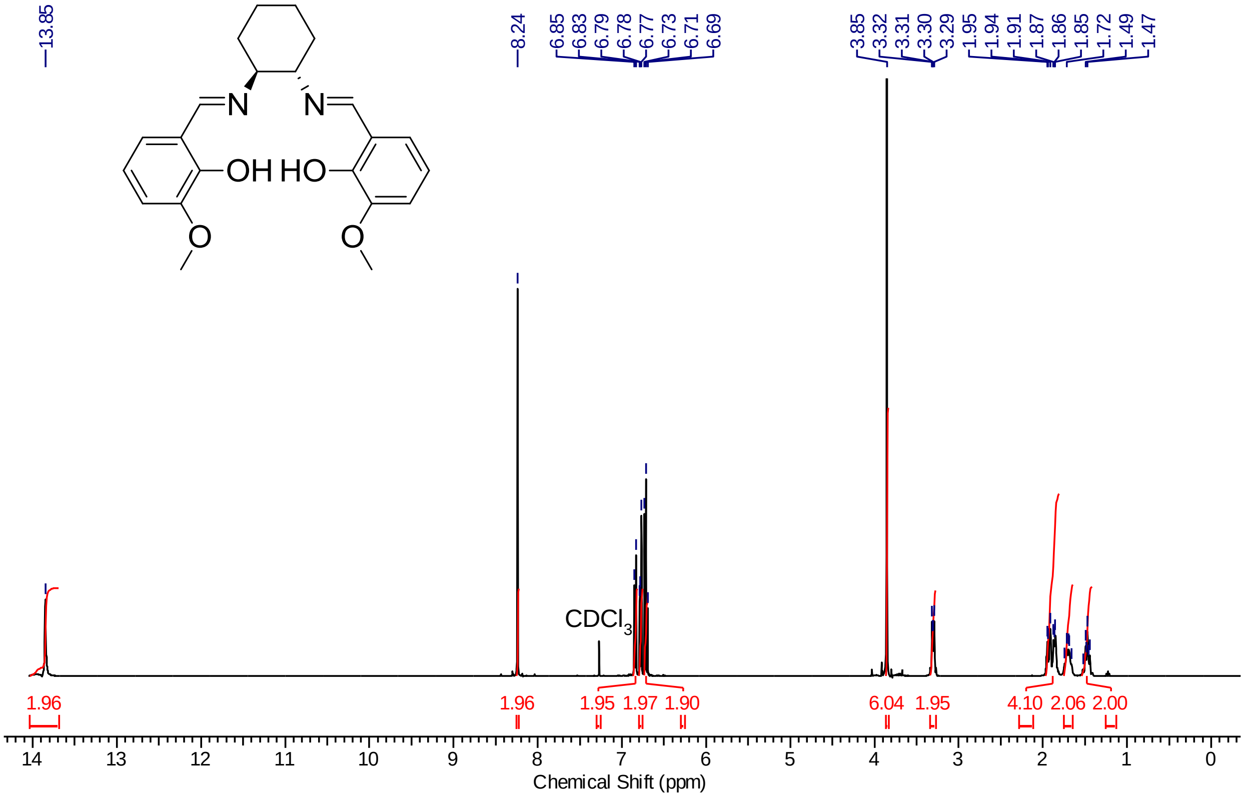

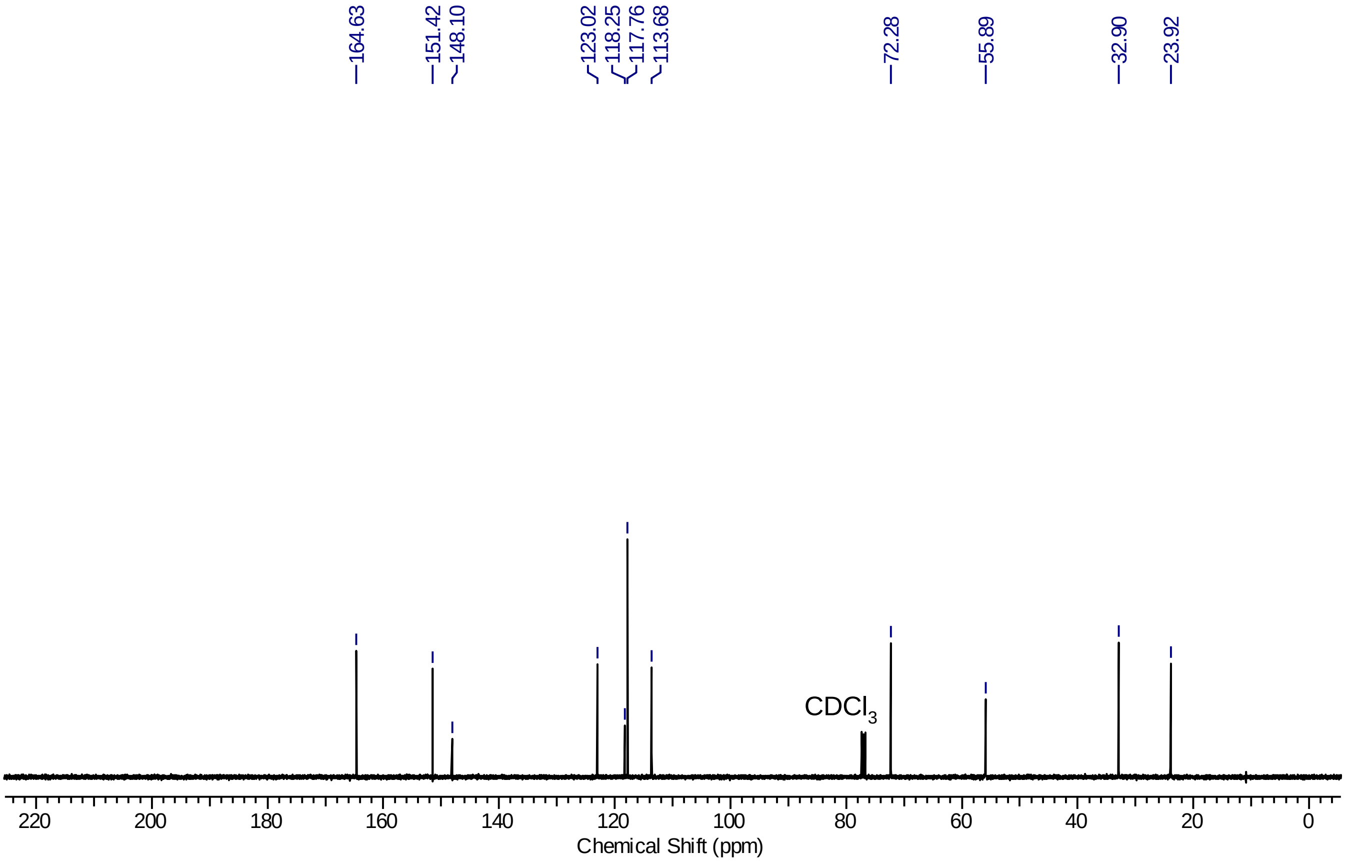

Ligand II

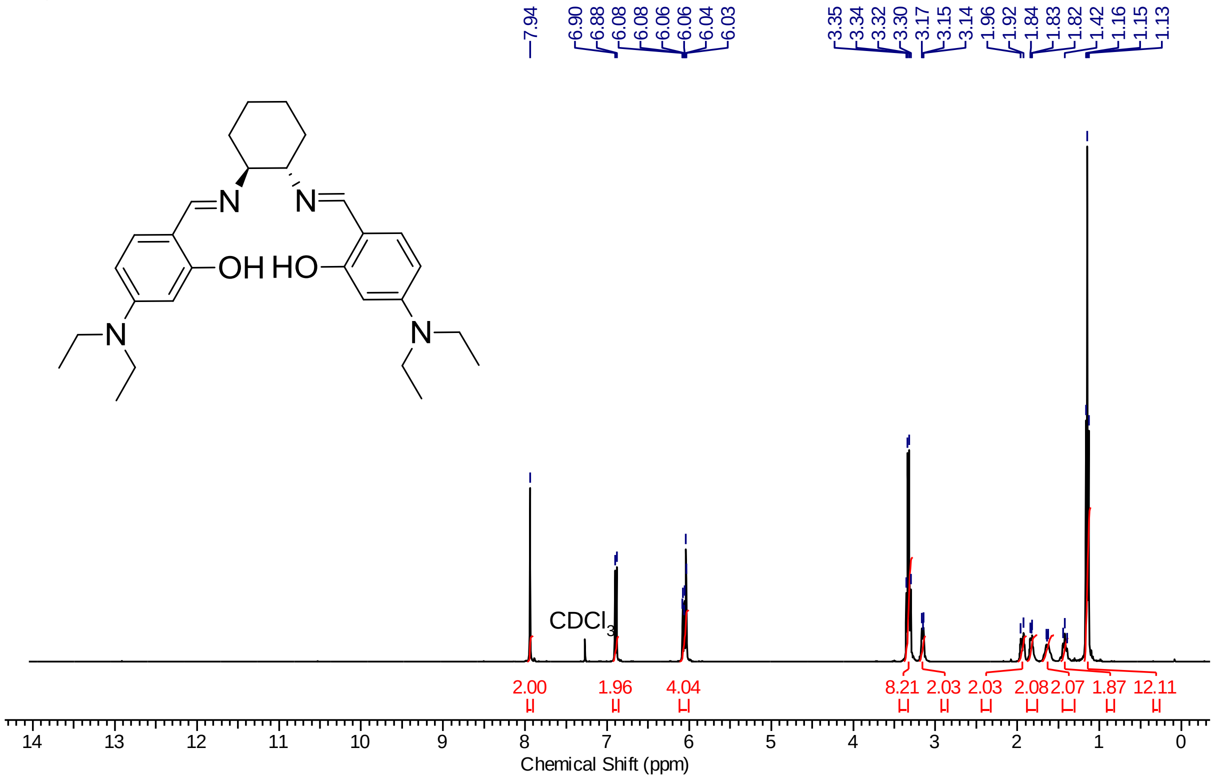

7.94
6.90 6.88 6.08 6.08 6.06 6.06 6.04 6.03
3.35 3.34 3.32 3.30 3.17 3.15 3.14 1.96 1.92 1.84 1.83 1.82 1.42 1.16 1.15 1.13
N N OH HO N N
CDCl3
2.00 1.96 4.04 8.21 2.03 2.03 2.08 2.07 1.87 12.11
14 13 12 11 10 9 8 7 6 5 4 3 2 1 0
Chemical Shift (ppm)

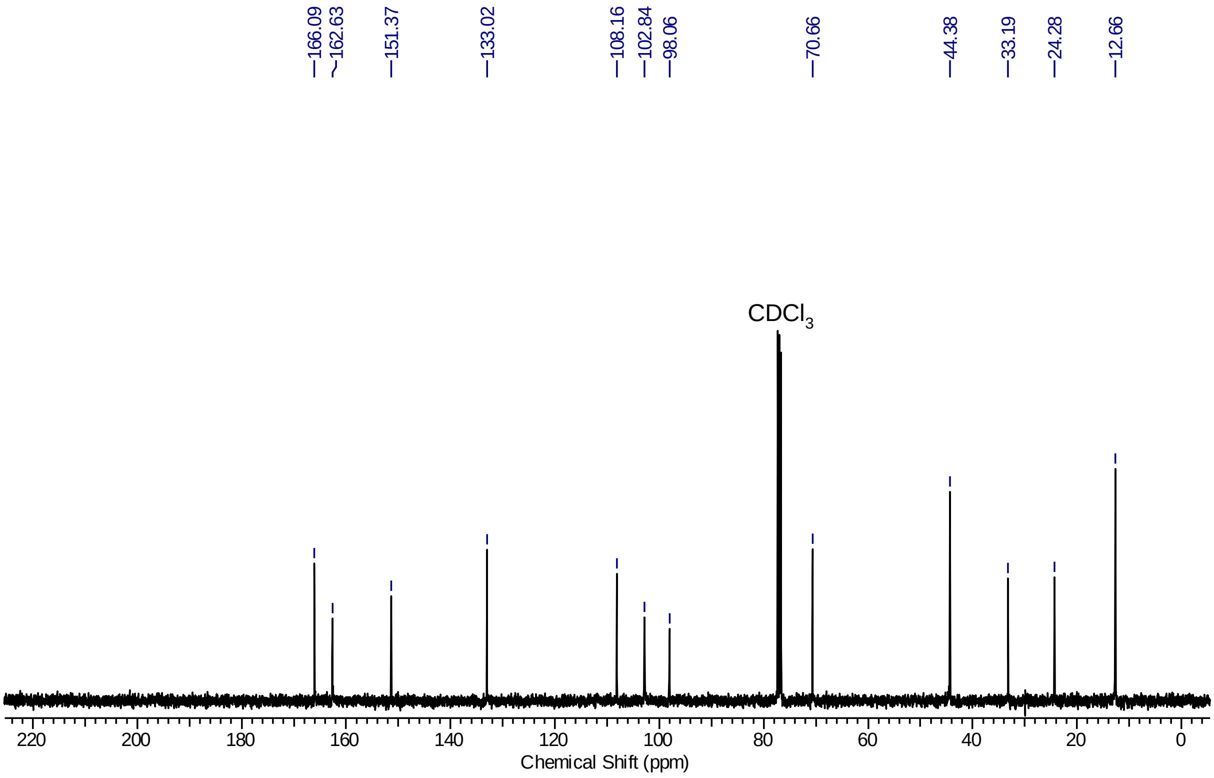

166.09 162.63 151.37 133.02 108.16 102.84 98.06 70.66 44.38 33.19 24.28 12.66
CDCl3
220 200 180 160 140 120 100 80 60 40 20 0
Chemical Shift (ppm)

Ligand III

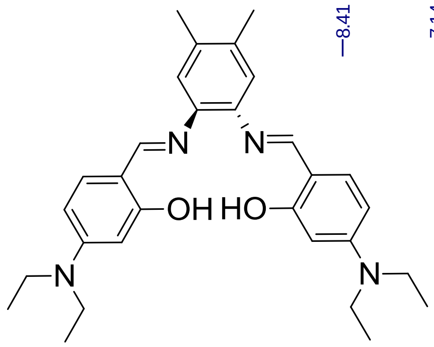
N
N
OH
HO
N
N

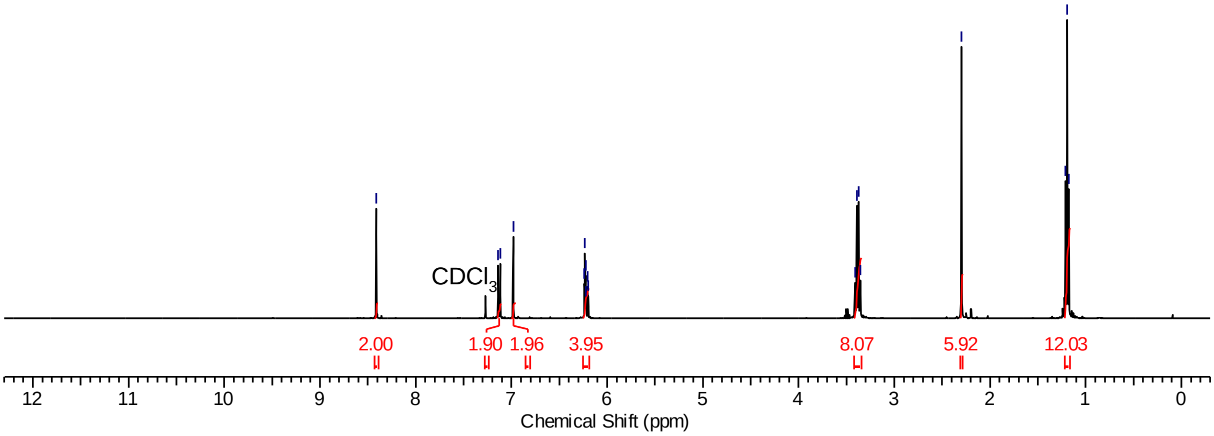
8.41
7.14
7.12
6.98
6.24
6.23
6.22
6.22
6.20
6.20
3.41
3.39
3.37
3.36
2.30
1.21
1.19
1.18
CDCl3
2.00
1.90
1.96
3.95
8.07
5.92
12.03
Chemical Shift (ppm)

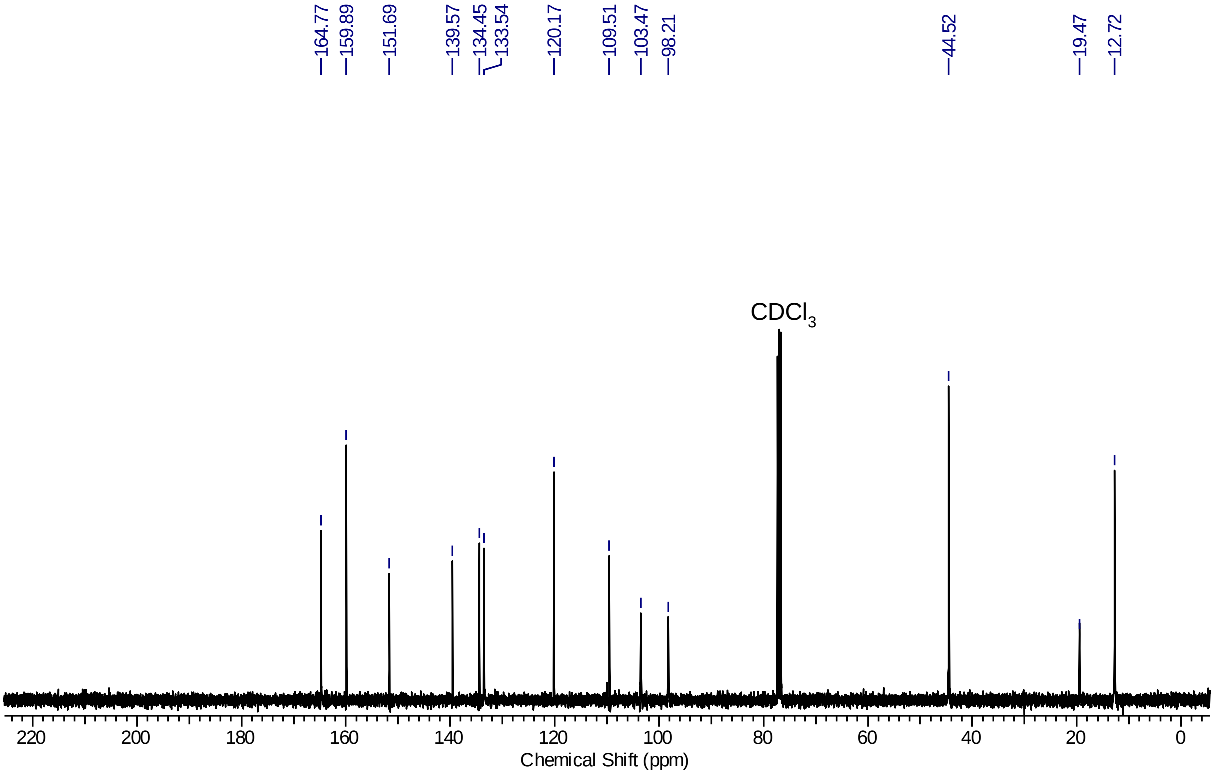
164.77
159.89
151.69
139.57
134.45
133.54
120.17
109.51
103.47
98.21
44.52
19.47
12.72
CDCl3
Chemical Shift (ppm)

Ligand IV

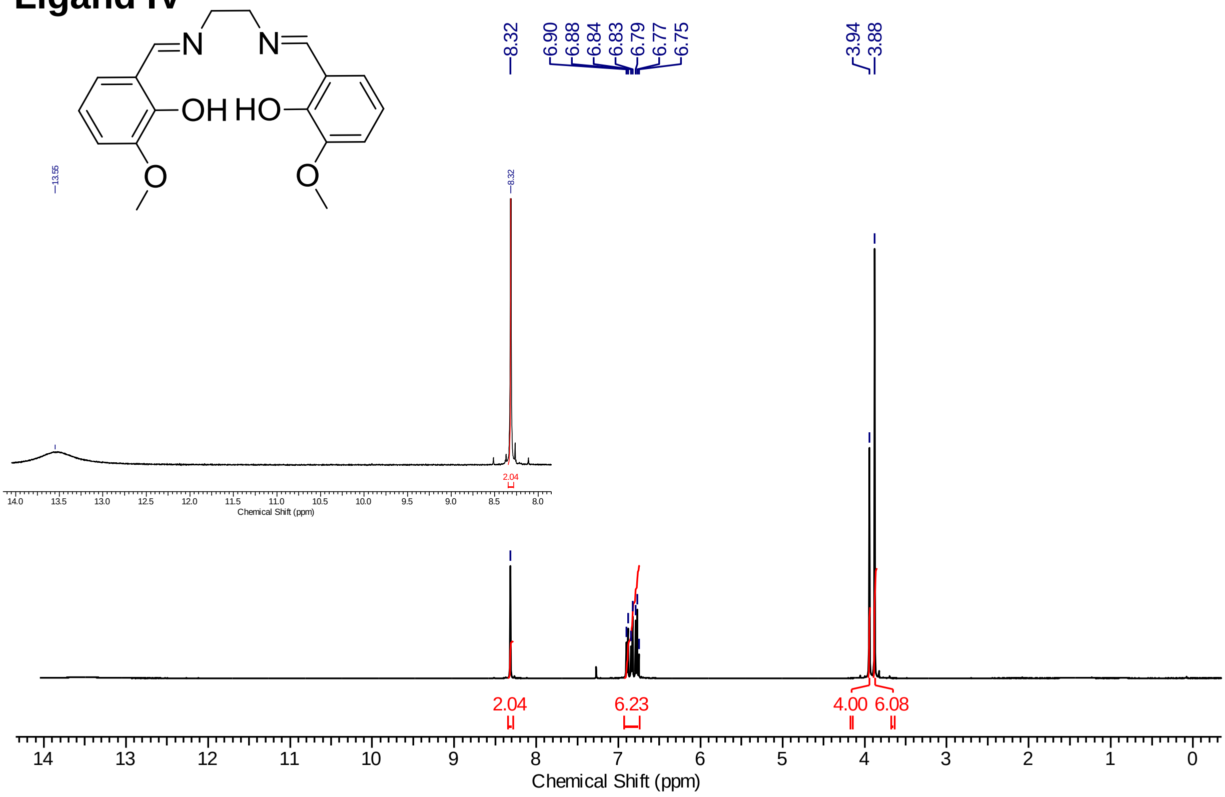

OH HO
N N
O O
8.32
6.90 6.88 6.84 6.83 6.79 6.77 6.75
3.94 3.88
13.55
2.04
6.23
4.00 6.08
Chemical Shift (ppm)

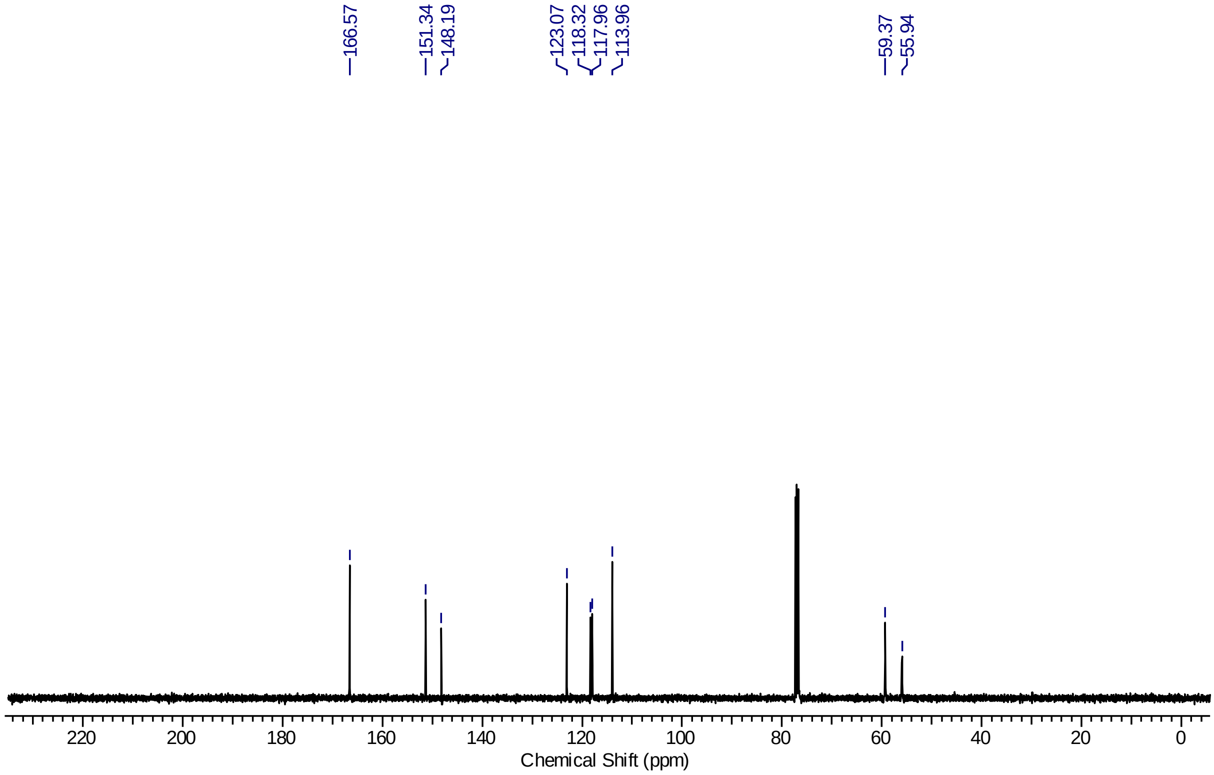

166.57
151.34 148.19
123.07 118.32 117.96 113.96
59.37 55.94
Chemical Shift (ppm)

Ligand V

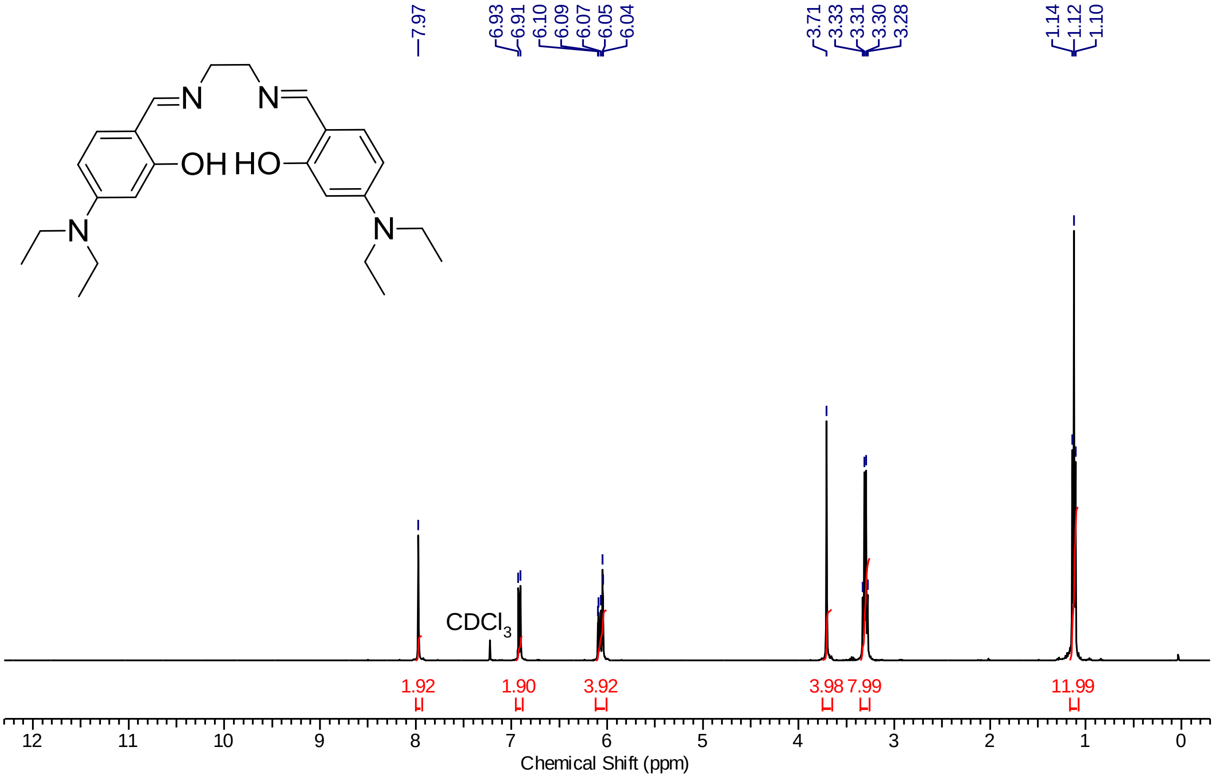

N
N
OH HO
N
N
7.97
6.93
6.91
6.10
6.09
6.07
6.05
6.04
3.71
3.33
3.31
3.30
3.28
1.14
1.12
1.10
CDCl3
1.92
1.90
3.92
3.98 7.99
11.99
12 11 10 9 8 7 6 5 4 3 2 1 0
Chemical Shift (ppm)

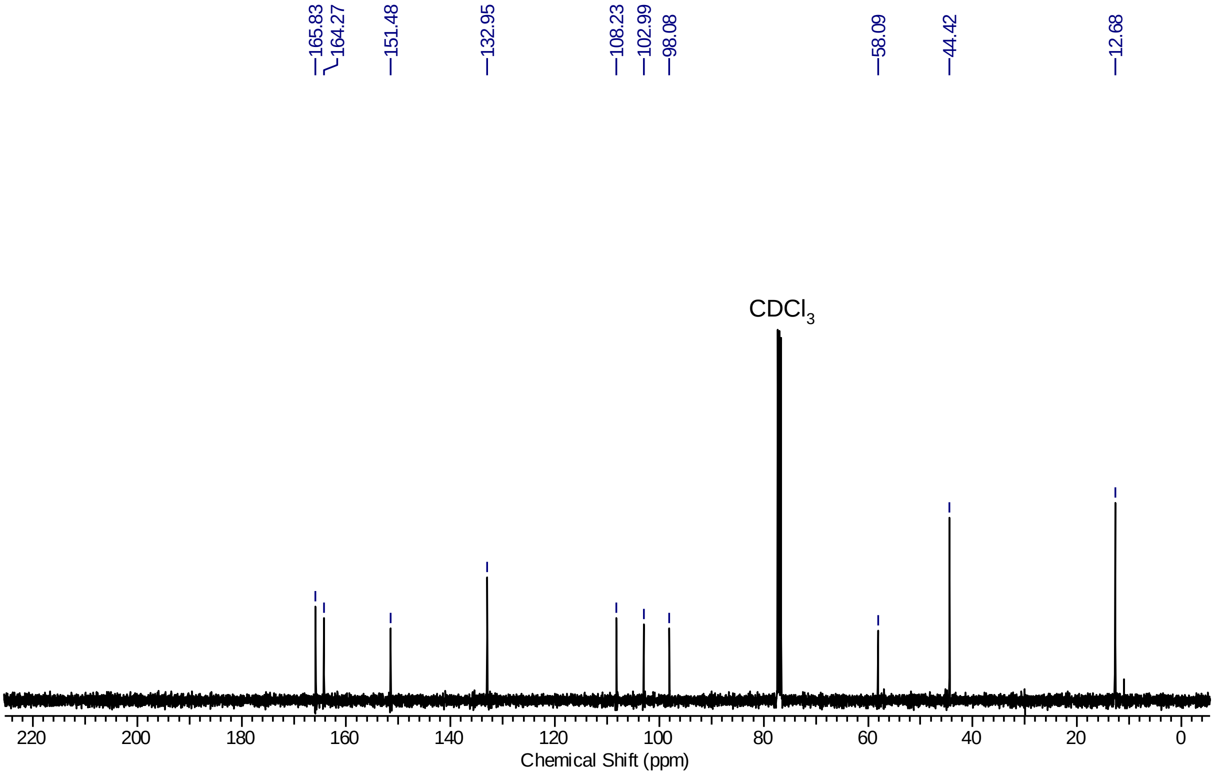

165.83
164.27
151.48
132.95
108.23
102.99
98.08
58.09
44.42
12.68
CDCl3
220 200 180 160 140 120 100 80 60 40 20 0
Chemical Shift (ppm)

Ligand VI

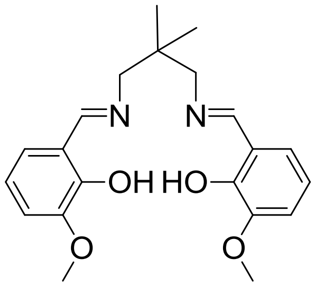

N
N
OH
HO
O
O

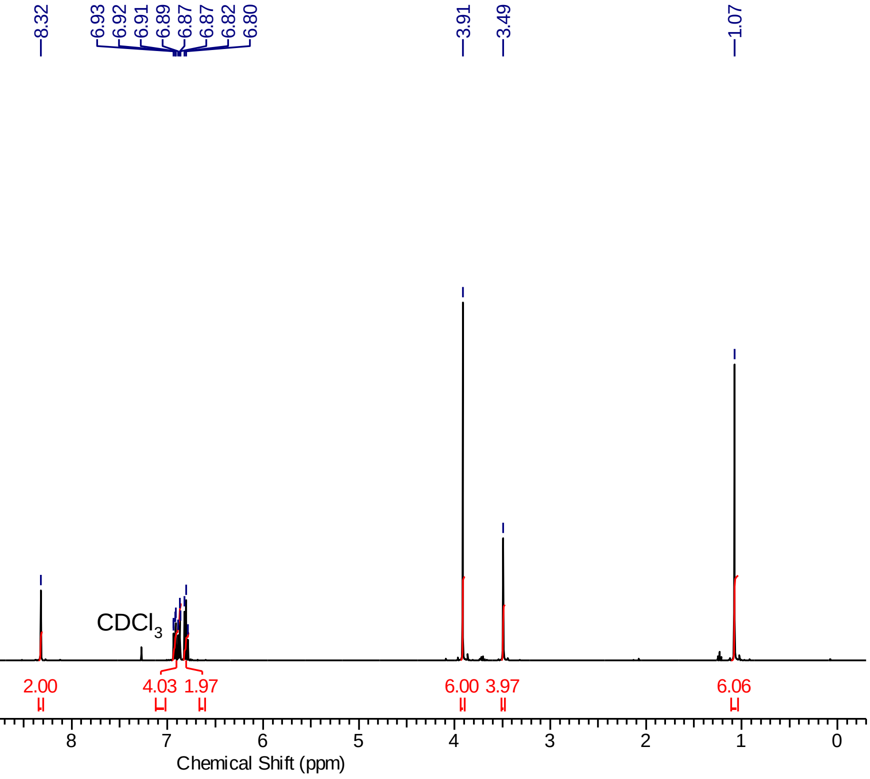

8.32
6.93
6.92
6.91
6.89
6.87
6.87
6.82
6.80
3.91
3.49
1.07
CDCl$_3$
2.00
4.03
1.97
6.00
3.97
6.06
12 11 10 9 8 7 6 5 4 3 2 1 0
Chemical Shift (ppm)

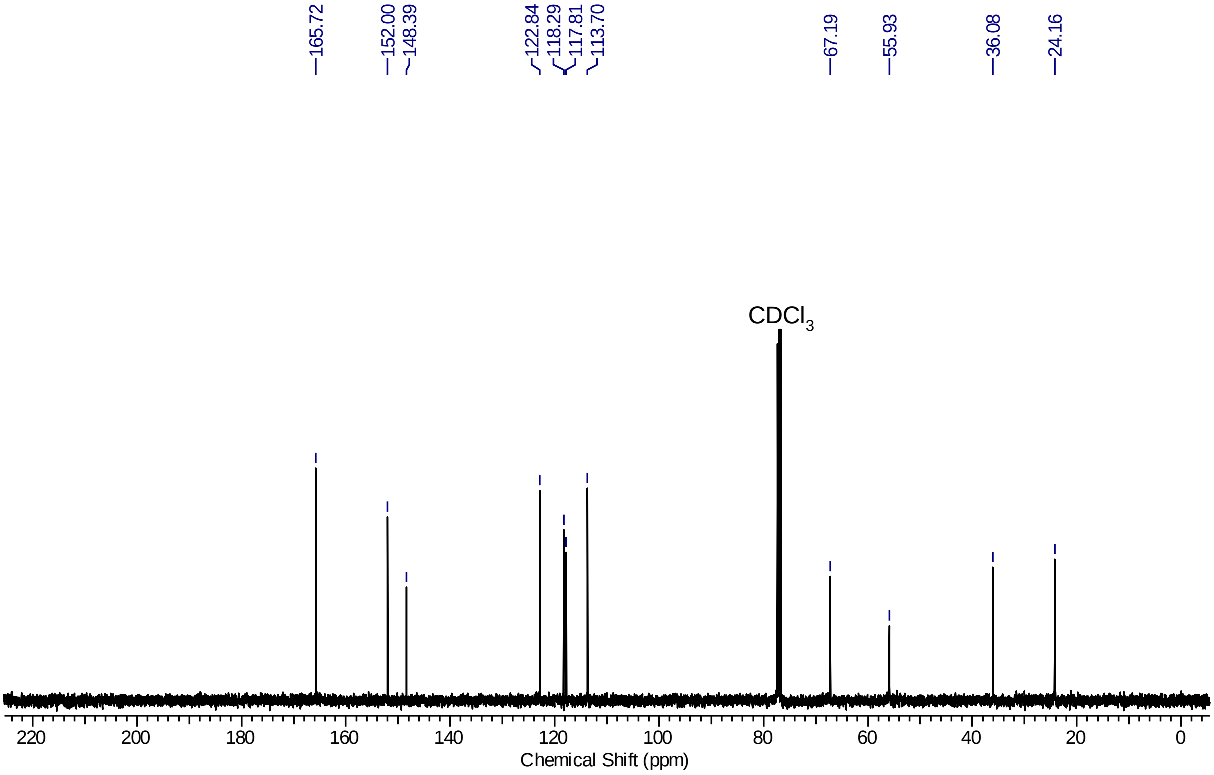

165.72
152.00
148.39
122.84
118.29
117.81
113.70
67.19
55.93
36.08
24.16
CDCl$_3$
220 200 180 160 140 120 100 80 60 40 20 0
Chemical Shift (ppm)

1:

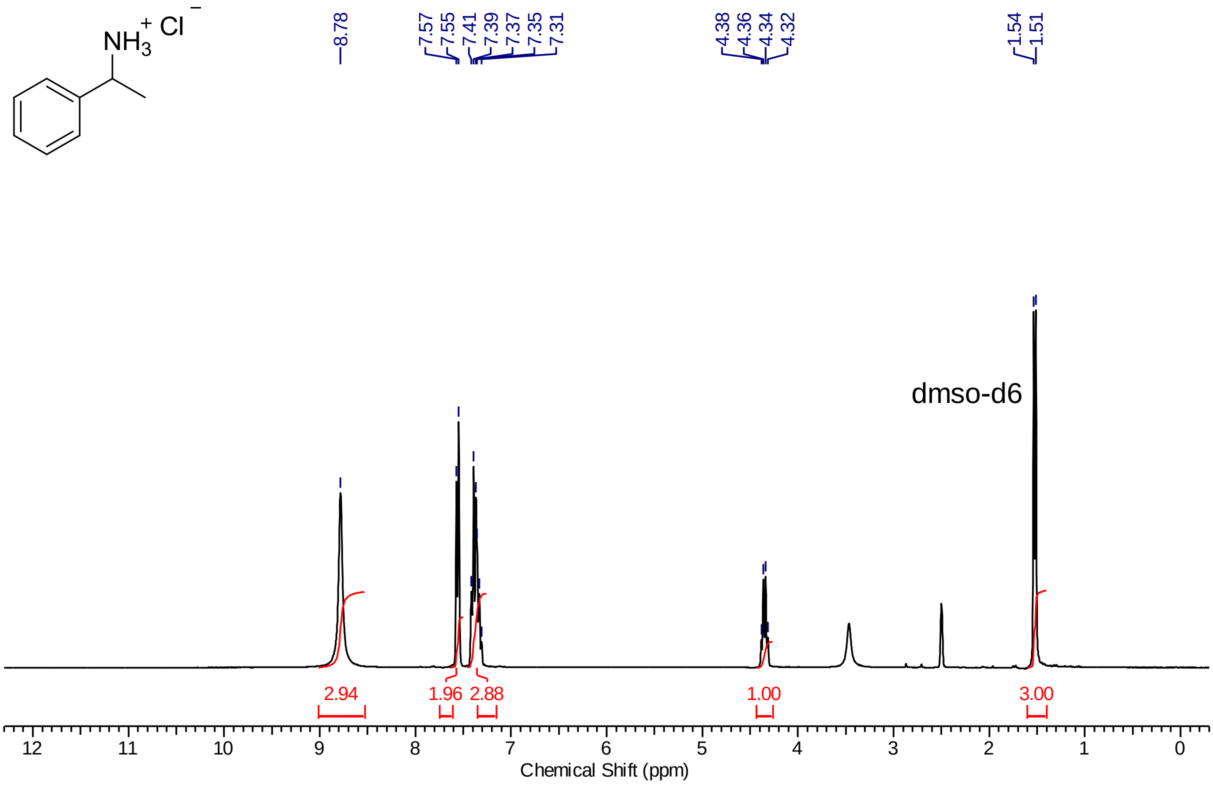
NH3+ Cl−
8.78
7.57
7.55
7.41
7.39
7.37
7.35
7.31
4.38
4.36
4.34
4.32
1.54
1.51
dmso-d6
2.94
1.96
2.88
1.00
3.00
12
11
10
9
8
7
6
5
4
3
2
1
0
Chemical Shift (ppm)

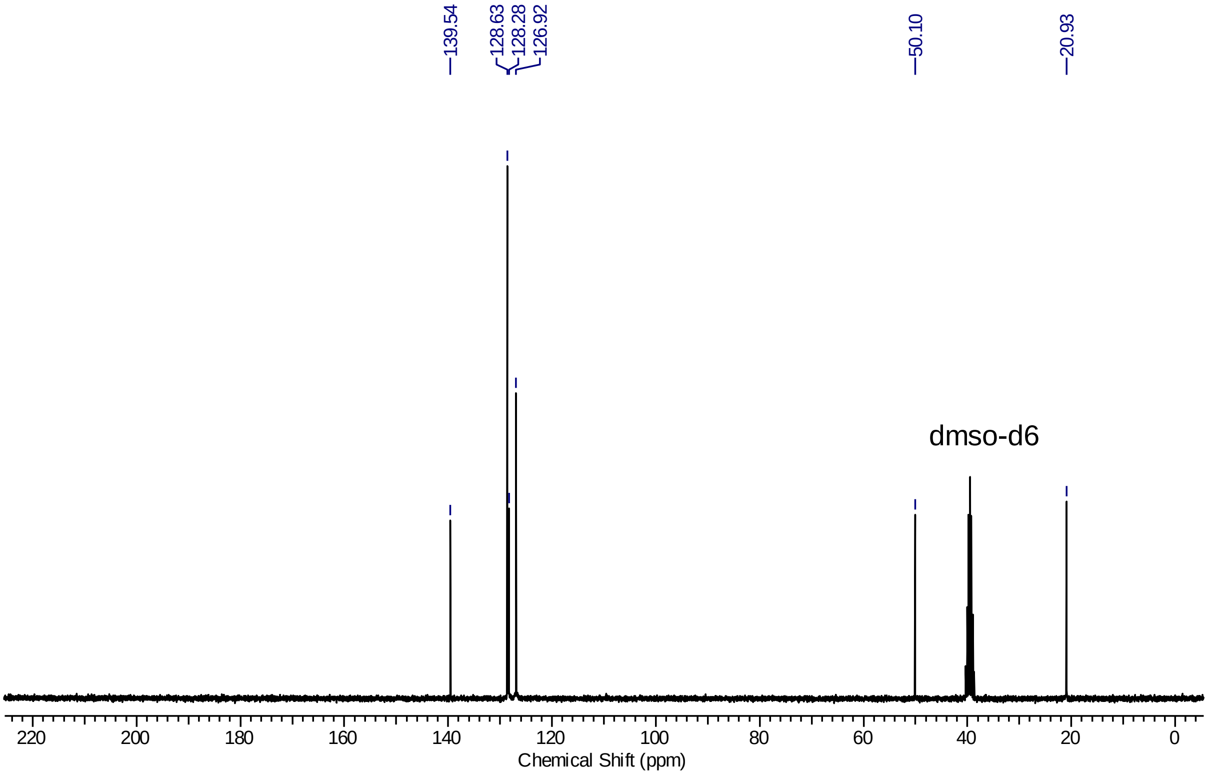
139.54
128.63
128.28
126.92
50.10
20.93
dmso-d6
220
200
180
160
140
120
100
80
60
40
20
0
Chemical Shift (ppm)

2:

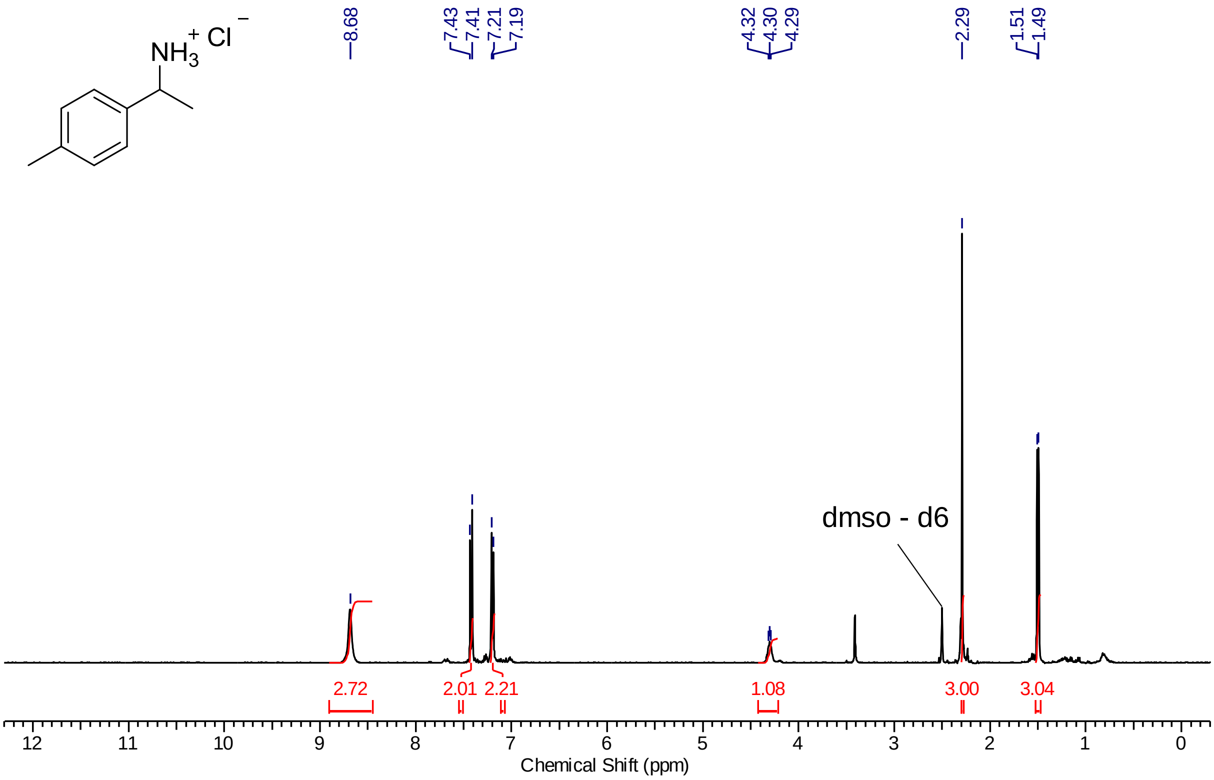

NH3+ Cl−
8.68
7.43
7.41
7.21
7.19
4.32
4.30
4.29
2.29
1.51
1.49
dmso - d6
2.72
2.01
2.21
1.08
3.00
3.04
12
11
10
9
8
7
6
5
4
3
2
1
0
Chemical Shift (ppm)

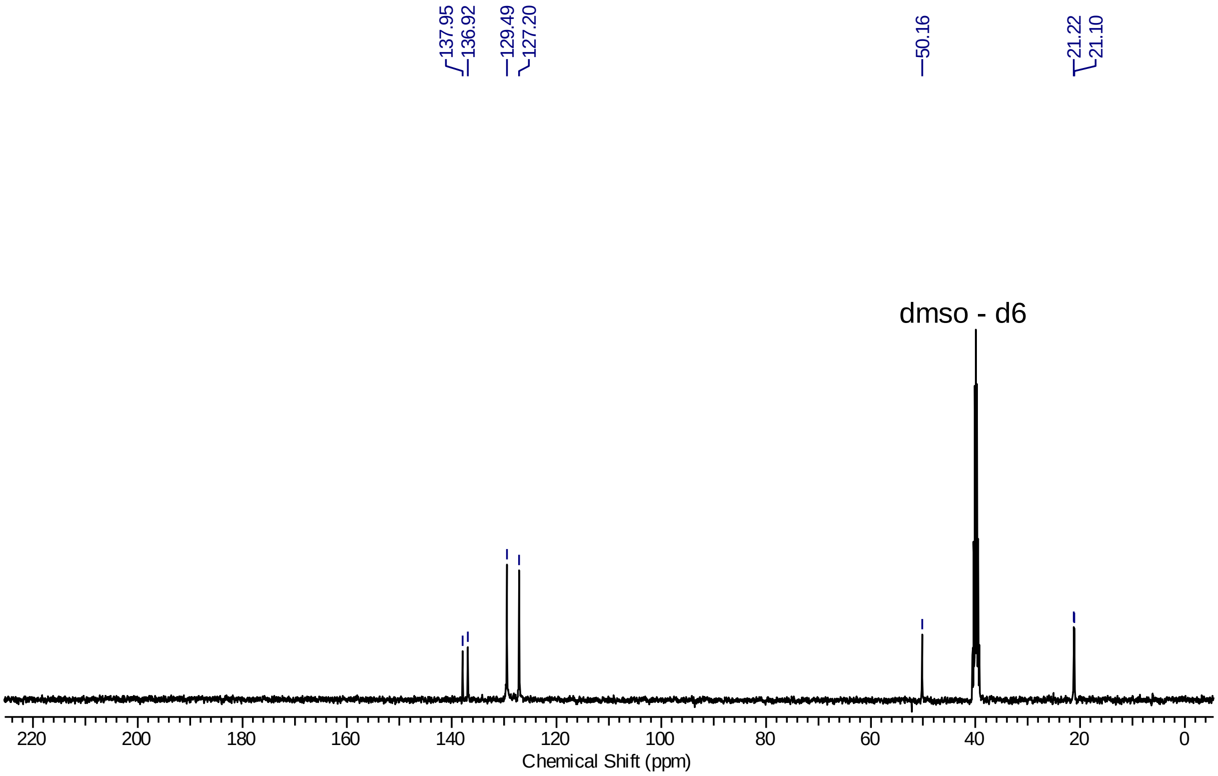

137.95
136.92
129.49
127.20
50.16
21.22
21.10
dmso - d6
220
200
180
160
140
120
100
80
60
40
20
0
Chemical Shift (ppm)

3:

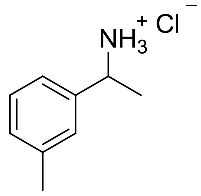
NH3+ Cl−

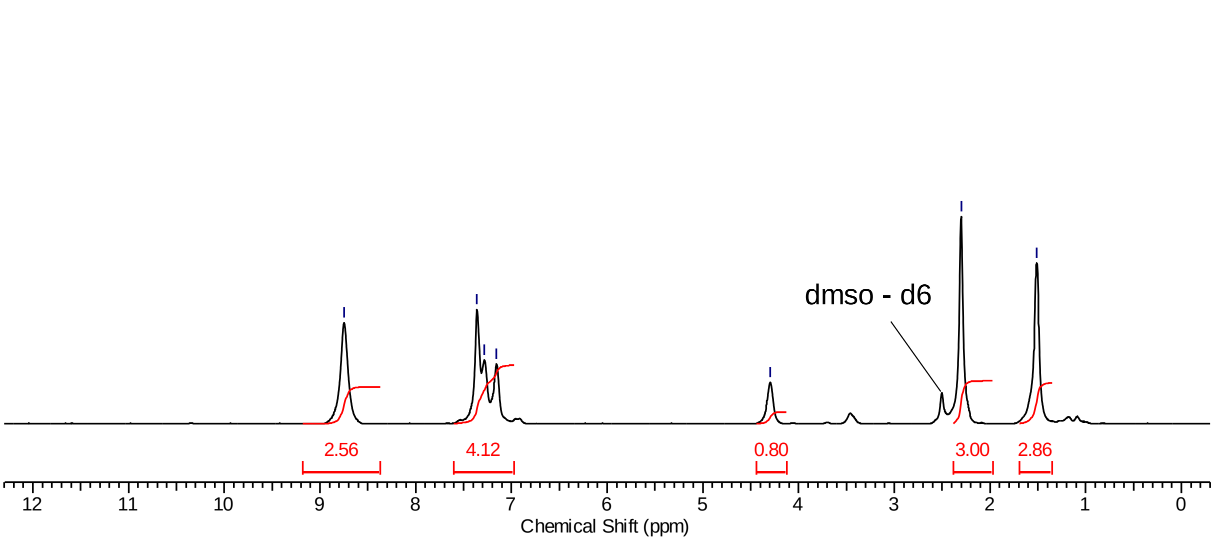
8.75
7.36
7.28
7.16
4.30
2.30
1.51
dmso - d6
2.56
4.12
0.80
3.00
2.86
12 11 10 9 8 7 6 5 4 3 2 1 0
Chemical Shift (ppm)

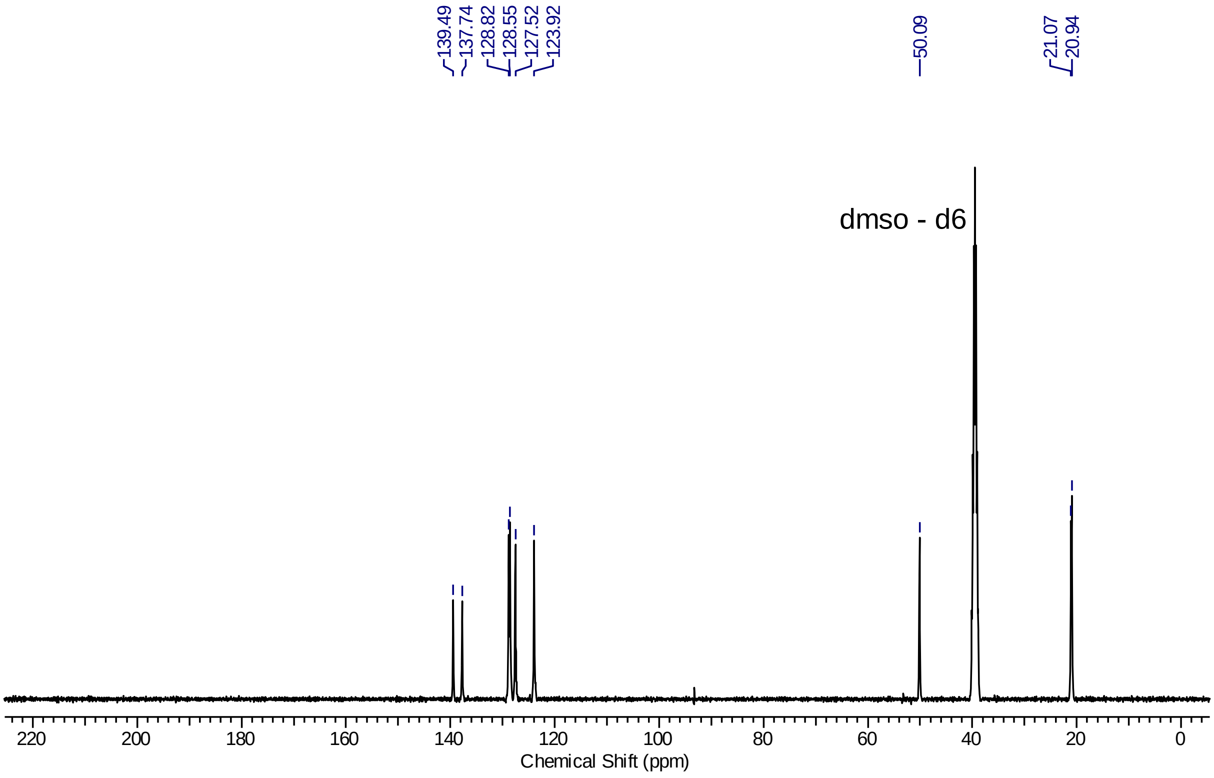
139.49
137.74
128.82
128.55
127.52
123.92
50.09
21.07
20.94
dmso - d6
220 200 180 160 140 120 100 80 60 40 20 0
Chemical Shift (ppm)

4:

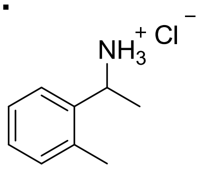
NH3+ Cl−

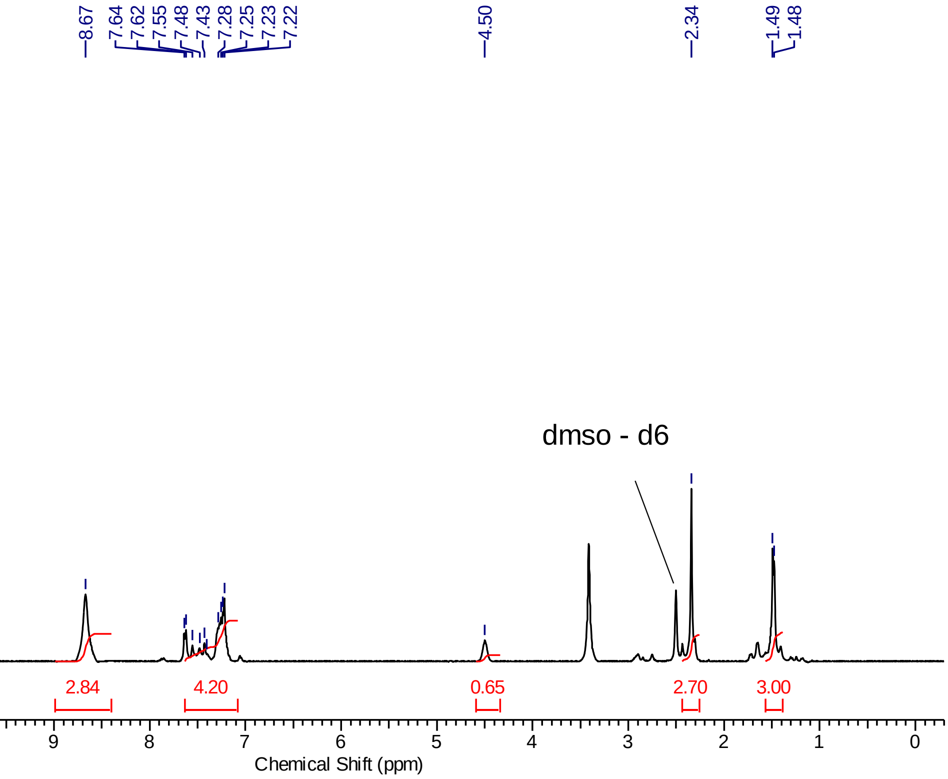
8.67
7.64
7.62
7.55
7.48
7.43
7.28
7.25
7.23
7.22
4.50
2.34
1.49
1.48
dmso - d6
2.84
4.20
0.65
2.70
3.00
12
11
10
9
8
7
6
5
4
3
2
1
0
Chemical Shift (ppm)

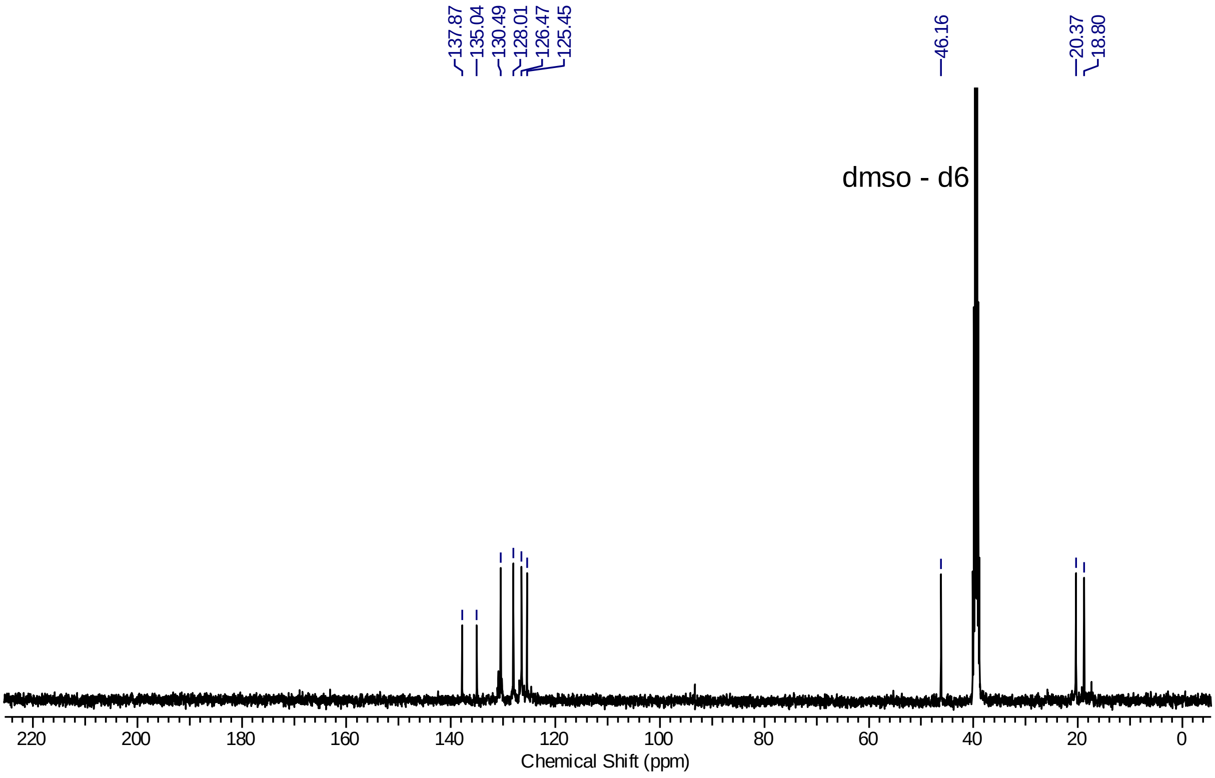
137.87
135.04
130.49
128.01
126.47
125.45
46.16
20.37
18.80
dmso - d6
220
200
180
160
140
120
100
80
60
40
20
0
Chemical Shift (ppm)

5:

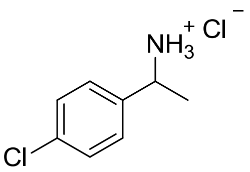
NH3+ Cl−
Cl

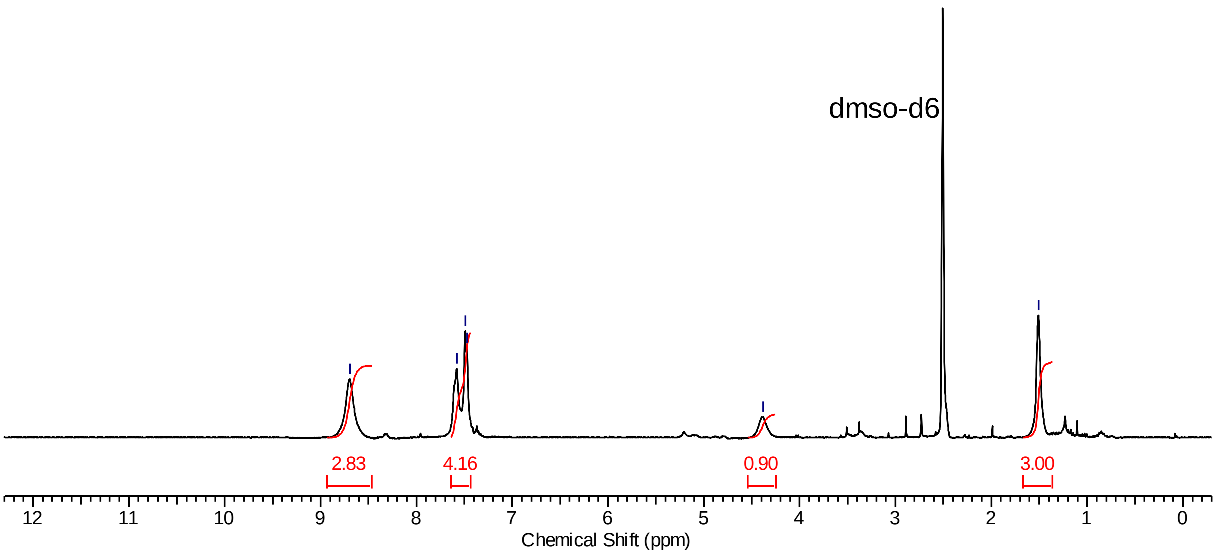
8.70
7.58
7.49
7.47
4.38
1.51
dmso-d6
2.83
4.16
0.90
3.00
12 11 10 9 8 7 6 5 4 3 2 1 0
Chemical Shift (ppm)

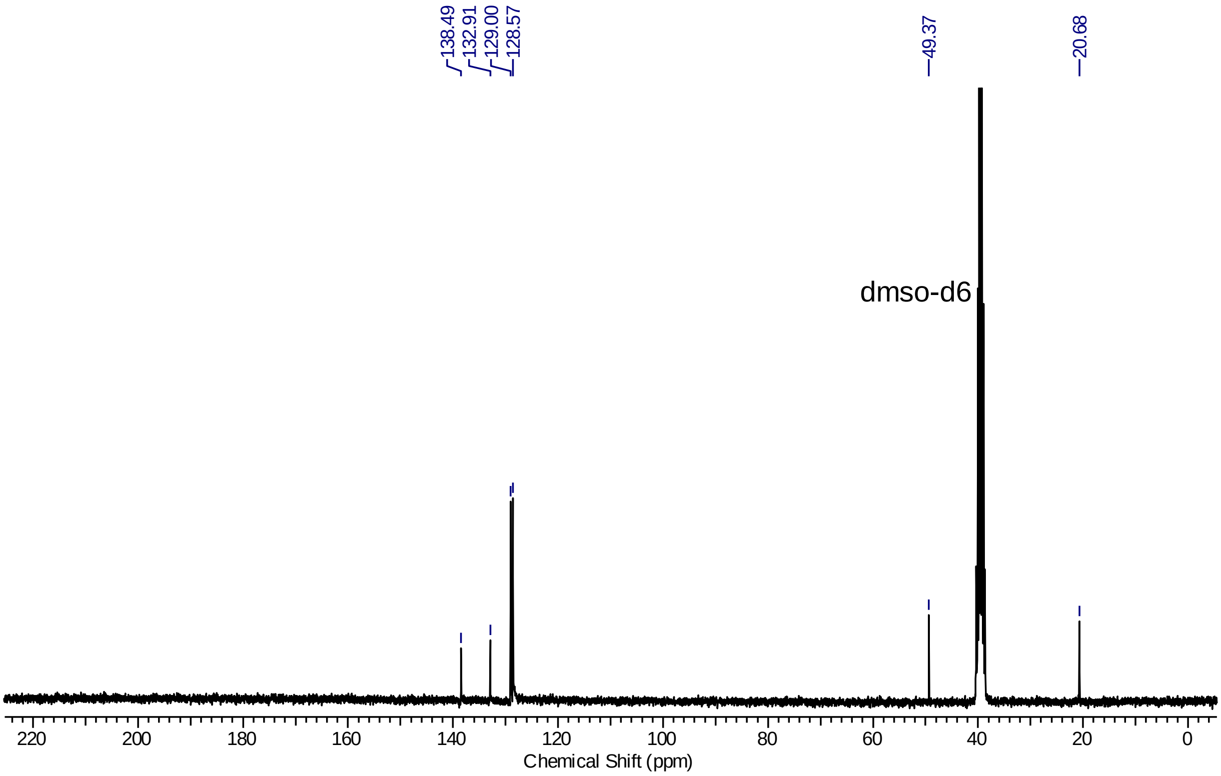
138.49
132.91
129.00
128.57
49.37
20.68
dmso-d6
220 200 180 160 140 120 100 80 60 40 20 0
Chemical Shift (ppm)

6:

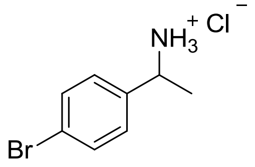

NH3+ Cl−
Br

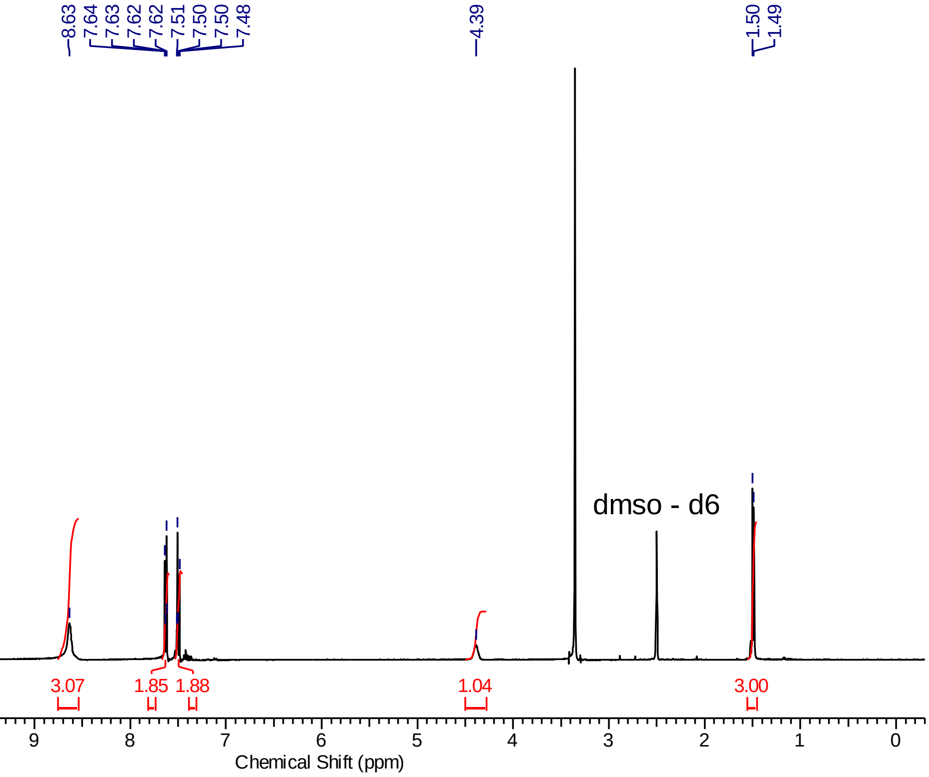

8.63
7.64
7.63
7.62
7.62
7.51
7.50
7.50
7.48
4.39
1.50
1.49
dmso - d6
3.07
1.85
1.88
1.04
3.00
Chemical Shift (ppm)

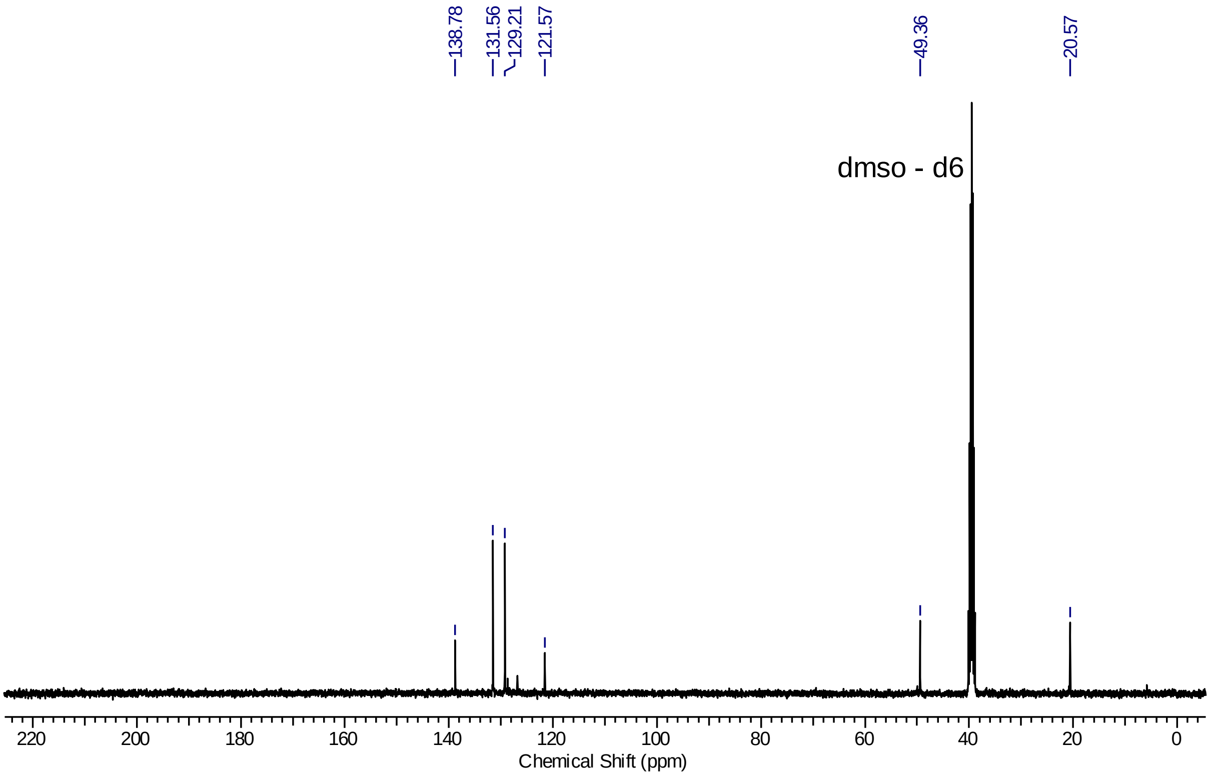

138.78
131.56
129.21
121.57
49.36
20.57
dmso - d6
Chemical Shift (ppm)

7:

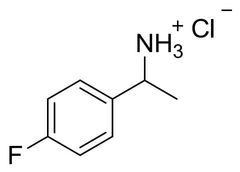

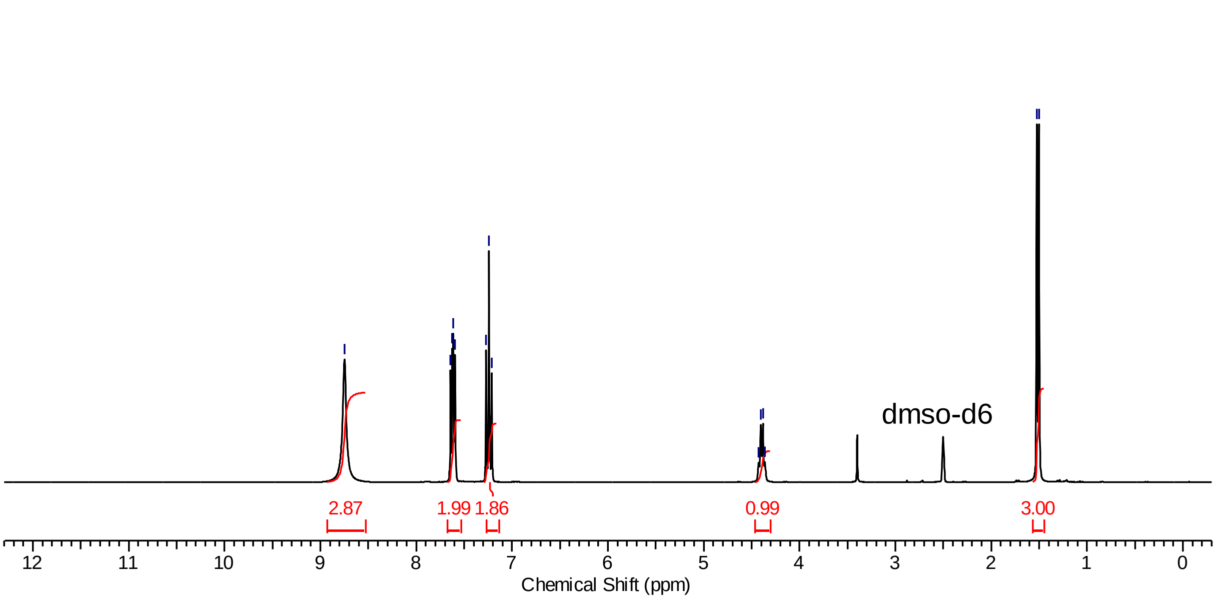

8.75
7.64
7.62
7.61
7.60
7.27
7.24
7.21
4.43
4.40
4.38
4.36
1.52
1.50
dmso-d6
2.87
1.99
1.86
0.99
3.00
12
11
10
9
8
7
6
5
4
3
2
1
0
Chemical Shift (ppm)

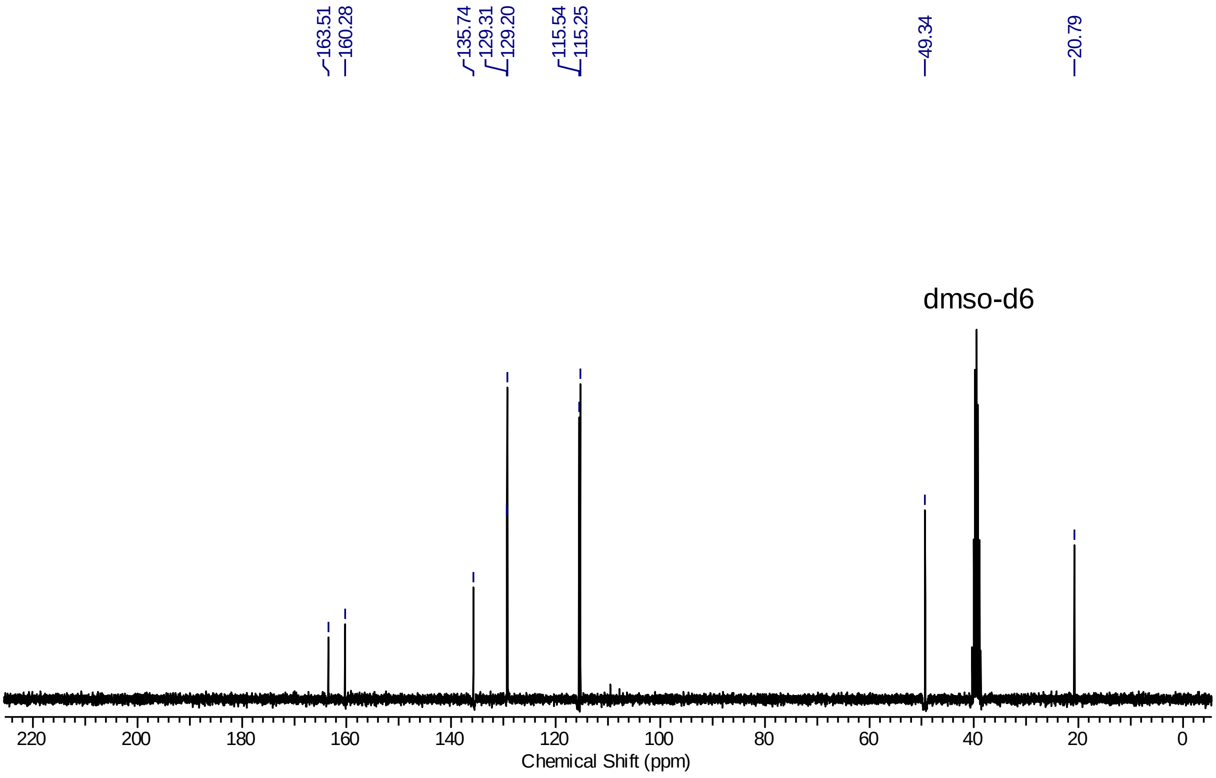

163.51
160.28
135.74
129.31
129.20
115.54
115.25
49.34
20.79
dmso-d6
220
200
180
160
140
120
100
80
60
40
20
0
Chemical Shift (ppm)

8:

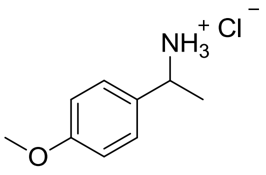
NH3+ Cl−
O

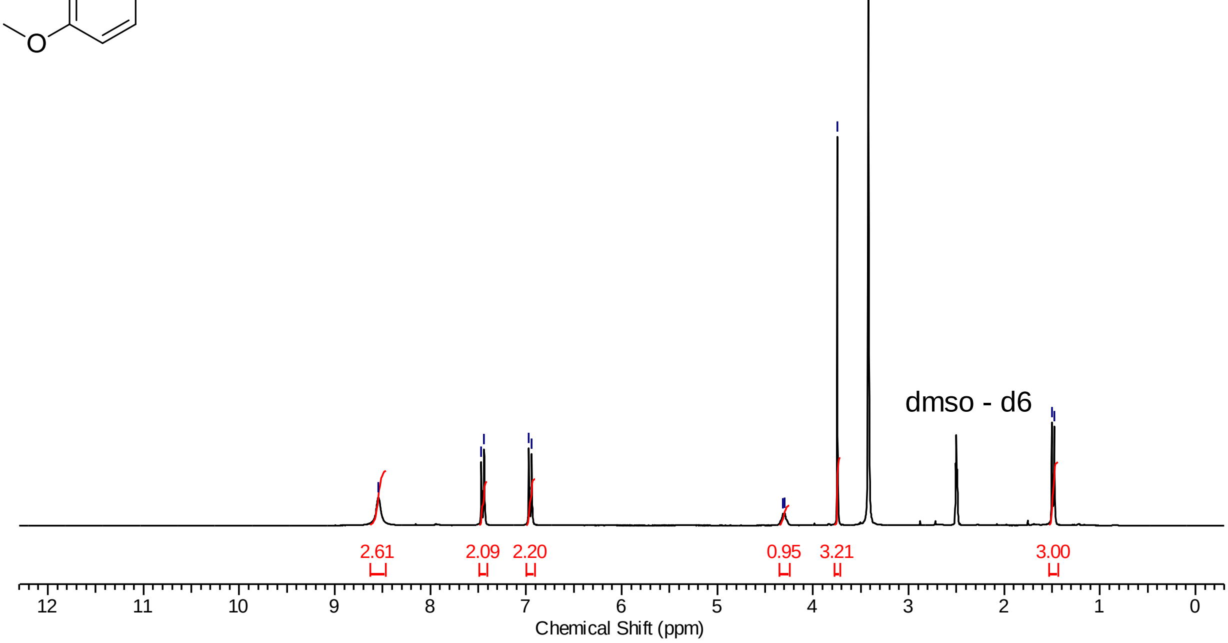
8.54
7.47
7.44
6.97
6.94
4.31
4.29
3.75
1.50
1.48
dmso - d6
2.61
2.09
2.20
0.95
3.21
3.00
12 11 10 9 8 7 6 5 4 3 2 1 0
Chemical Shift (ppm)

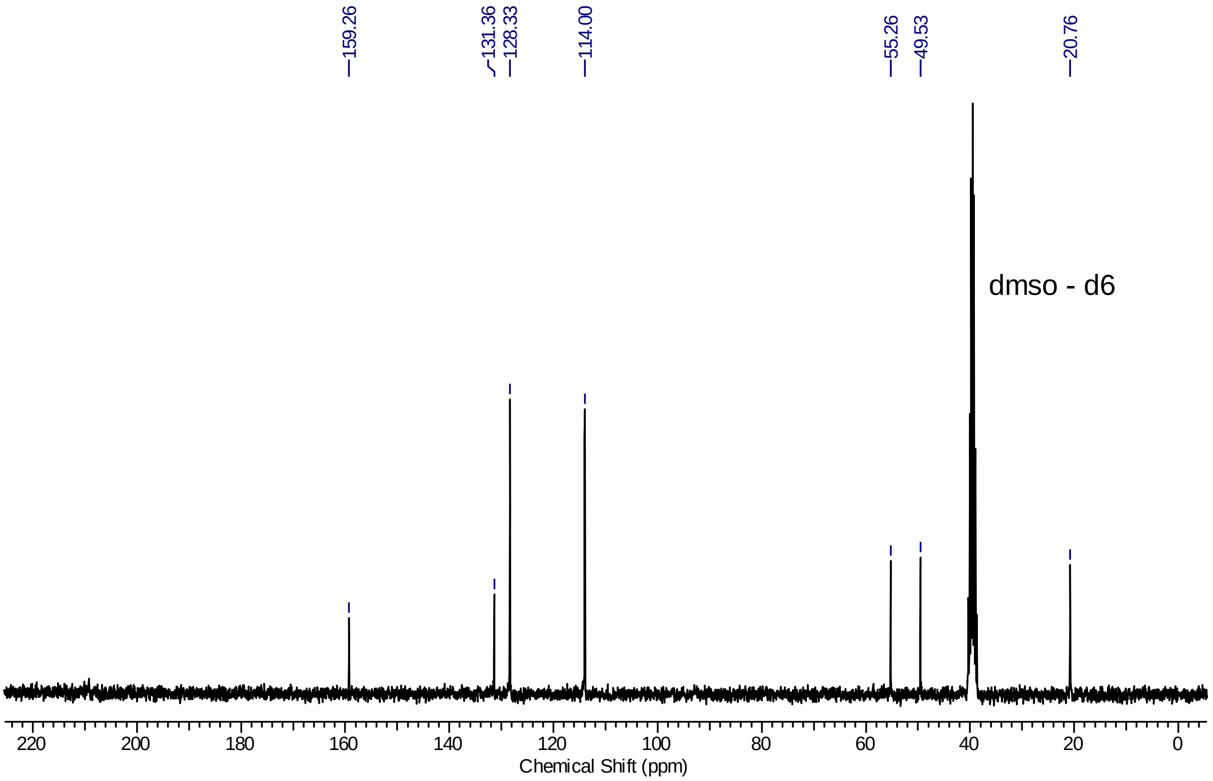
159.26
131.36
128.33
114.00
55.26
49.53
20.76
dmso - d6
220 200 180 160 140 120 100 80 60 40 20 0
Chemical Shift (ppm)

9:

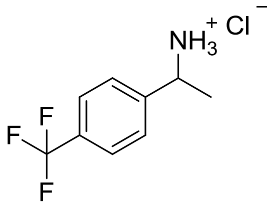

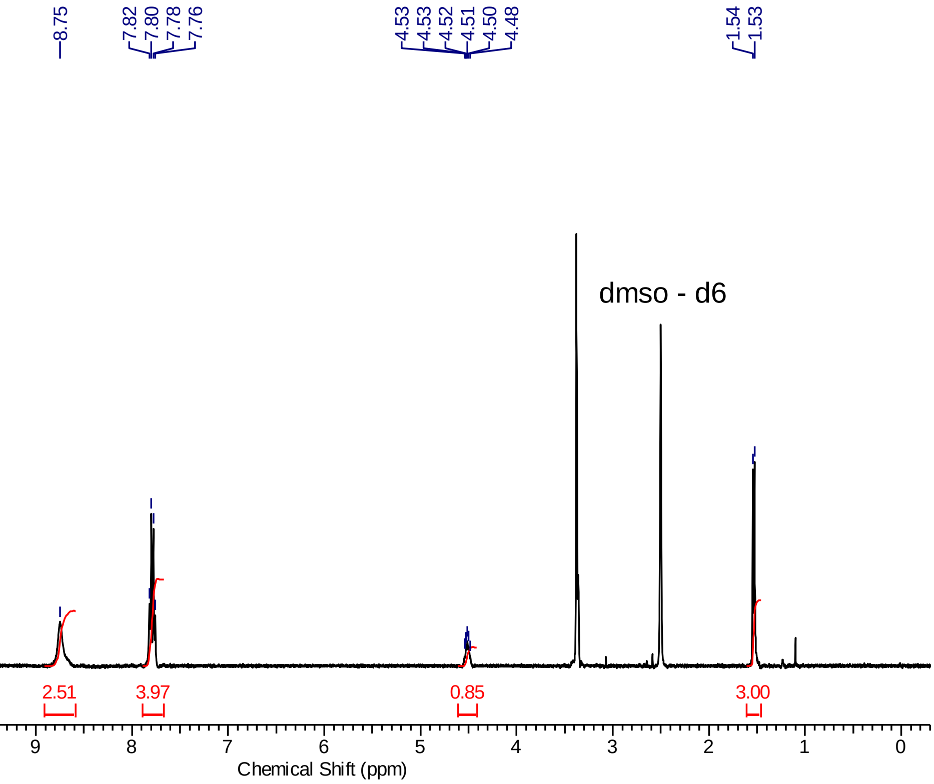
8.75
7.82
7.80
7.78
7.76
4.53
4.53
4.52
4.51
4.50
4.48
1.54
1.53
dmso - d6
2.51
3.97
0.85
3.00
Chemical Shift (ppm)

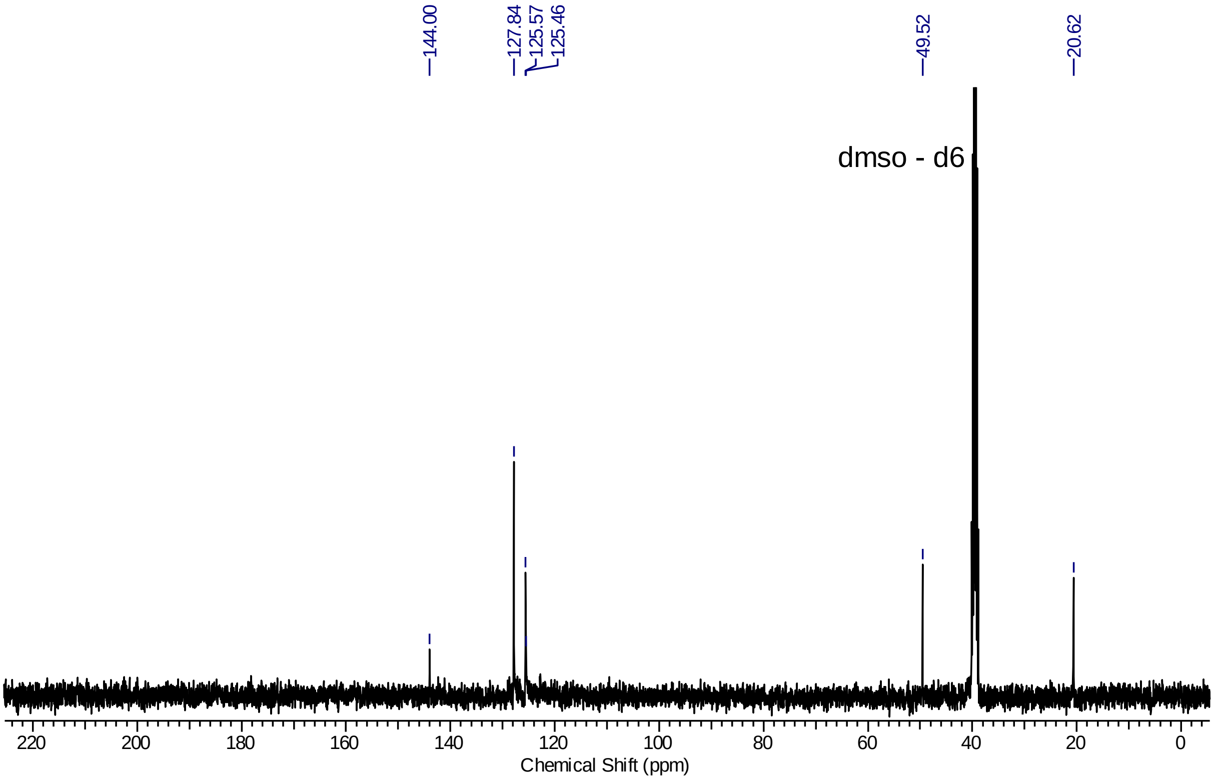
144.00
127.84
125.57
125.46
49.52
20.62
dmso - d6
Chemical Shift (ppm)

10:

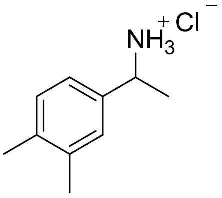
NH3+ Cl−

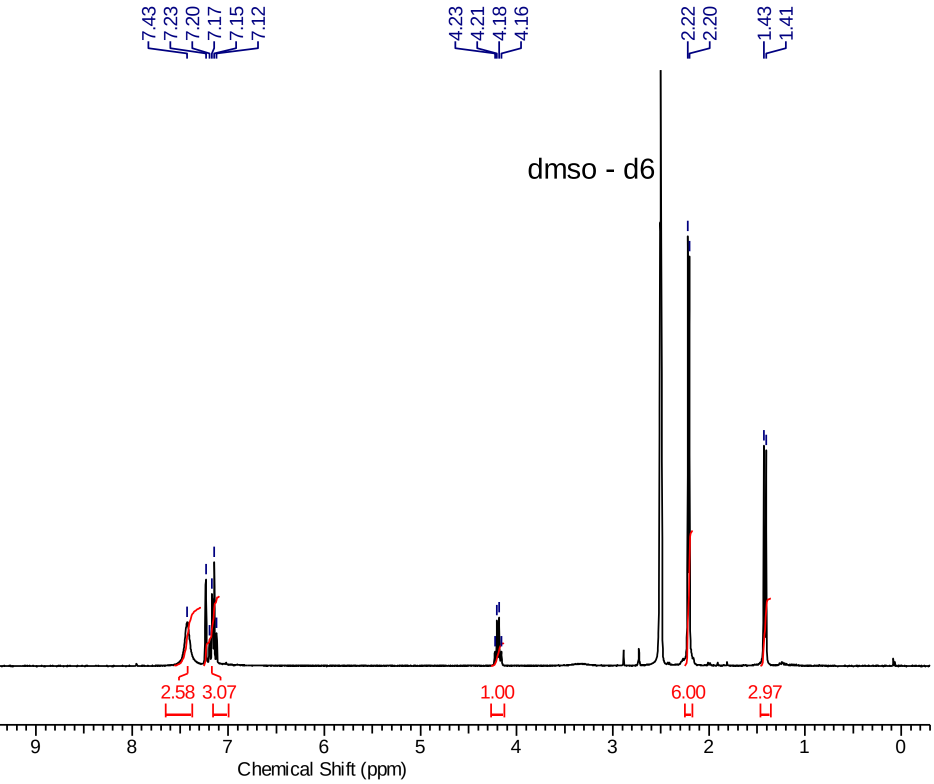
7.43
7.23
7.20
7.17
7.15
7.12
4.23
4.21
4.18
4.16
2.22
2.20
1.43
1.41
dmso - d6
2.58
3.07
1.00
6.00
2.97
12
11
10
9
8
7
6
5
4
3
2
1
0
Chemical Shift (ppm)

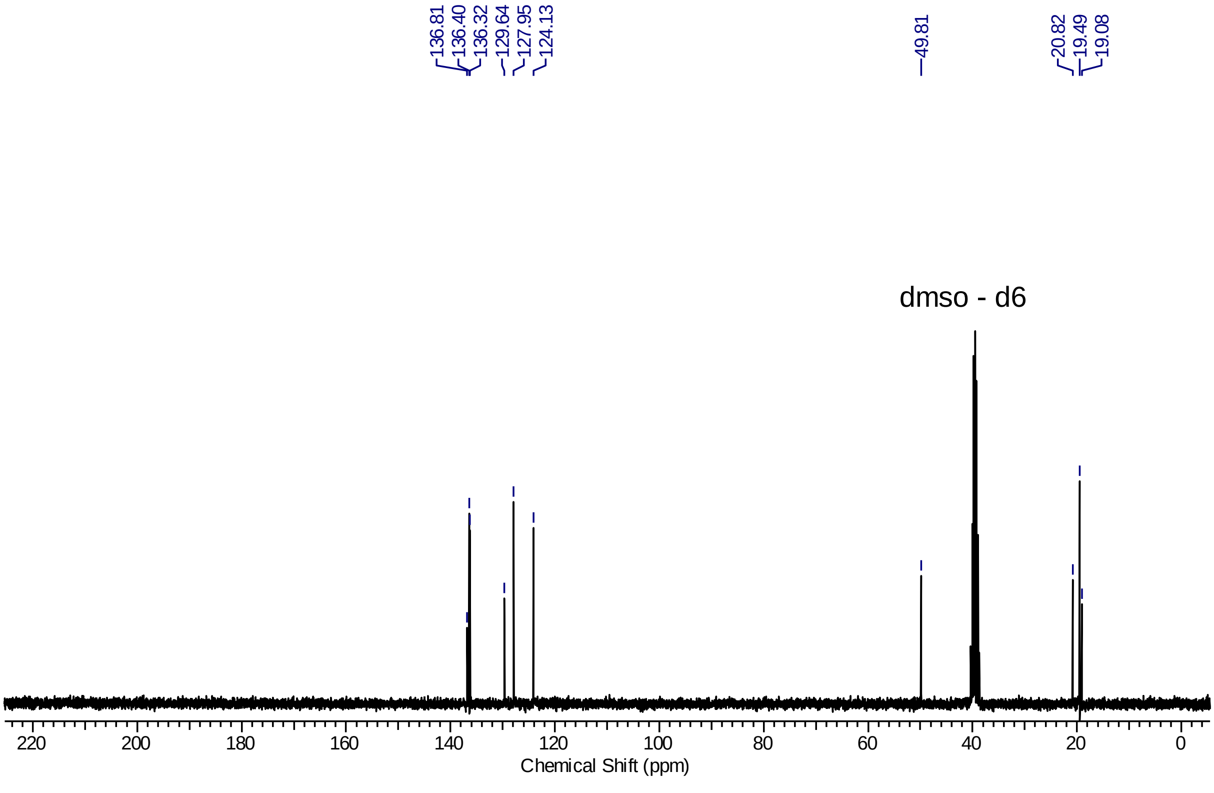
136.81
136.40
136.32
129.64
127.95
124.13
49.81
20.82
19.49
19.08
dmso - d6
220
200
180
160
140
120
100
80
60
40
20
0
Chemical Shift (ppm)

11:

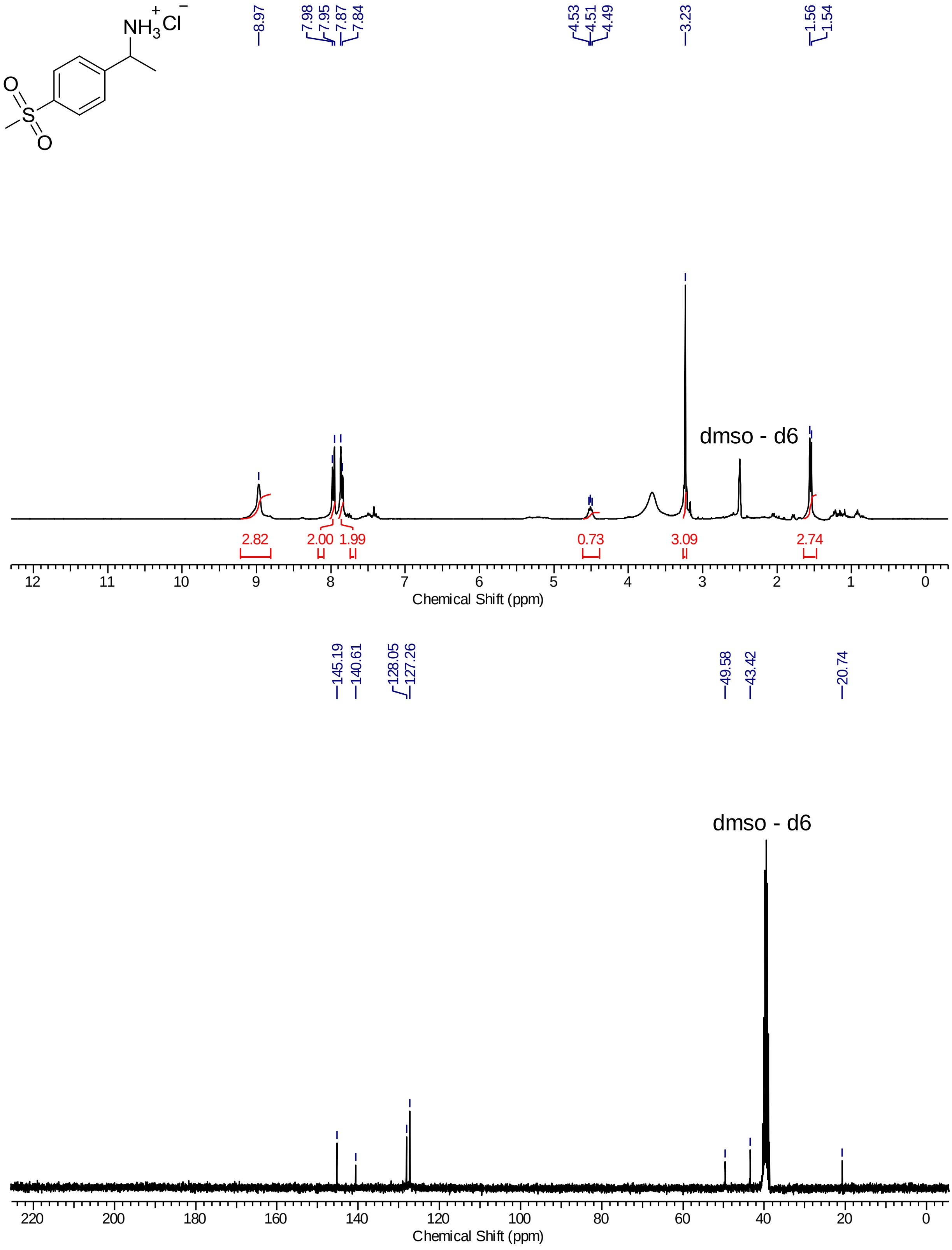
NH3+ Cl−
O
S
O
8.97
7.98
7.95
7.87
7.84
4.53
4.51
4.49
3.23
1.56
1.54
dmso - d6
2.82
2.00
1.99
0.73
3.09
2.74
12
11
10
9
8
7
6
5
4
3
2
1
0
Chemical Shift (ppm)
145.19
140.61
128.05
127.26
49.58
43.42
20.74
dmso - d6
220
200
180
160
140
120
100
80
60
40
20
0
Chemical Shift (ppm)

12:

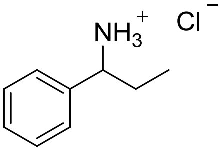
NH3+
Cl−

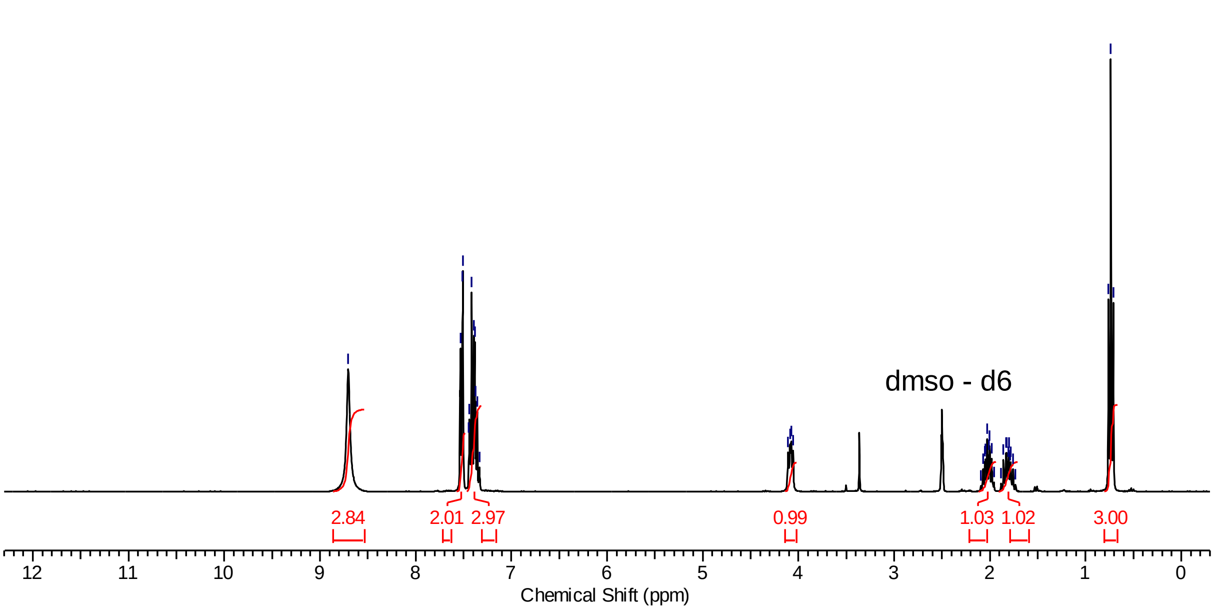
8.70
7.53
7.51
7.51
7.44
7.41
7.39
7.38
7.38
4.11
4.09
4.08
4.06
2.05
2.05
2.03
2.01
2.00
1.98
1.83
1.83
1.80
0.76
0.74
0.71
dmso - d6
2.84
2.01
2.97
0.99
1.03
1.02
3.00
12 11 10 9 8 7 6 5 4 3 2 1 0
Chemical Shift (ppm)

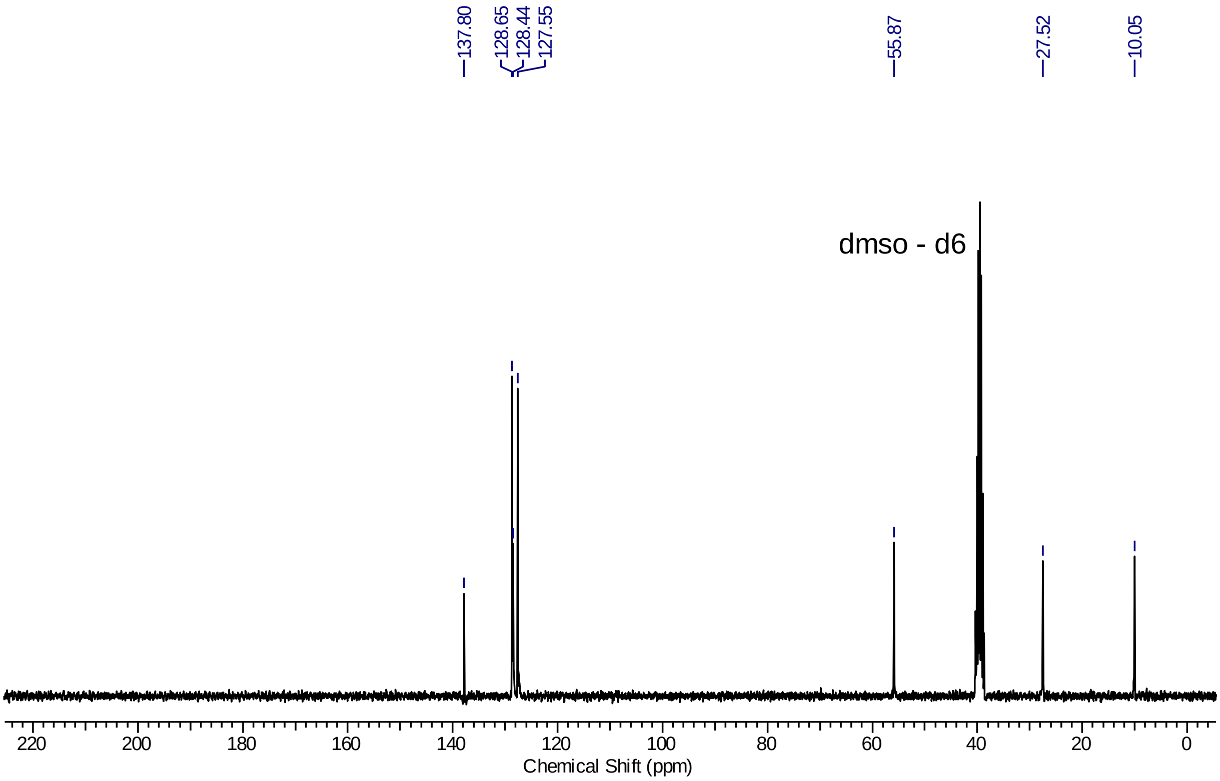
137.80
128.65
128.44
127.55
55.87
27.52
10.05
dmso - d6
220 200 180 160 140 120 100 80 60 40 20 0
Chemical Shift (ppm)

13:

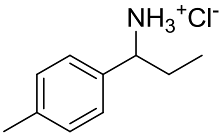
NH3+Cl-

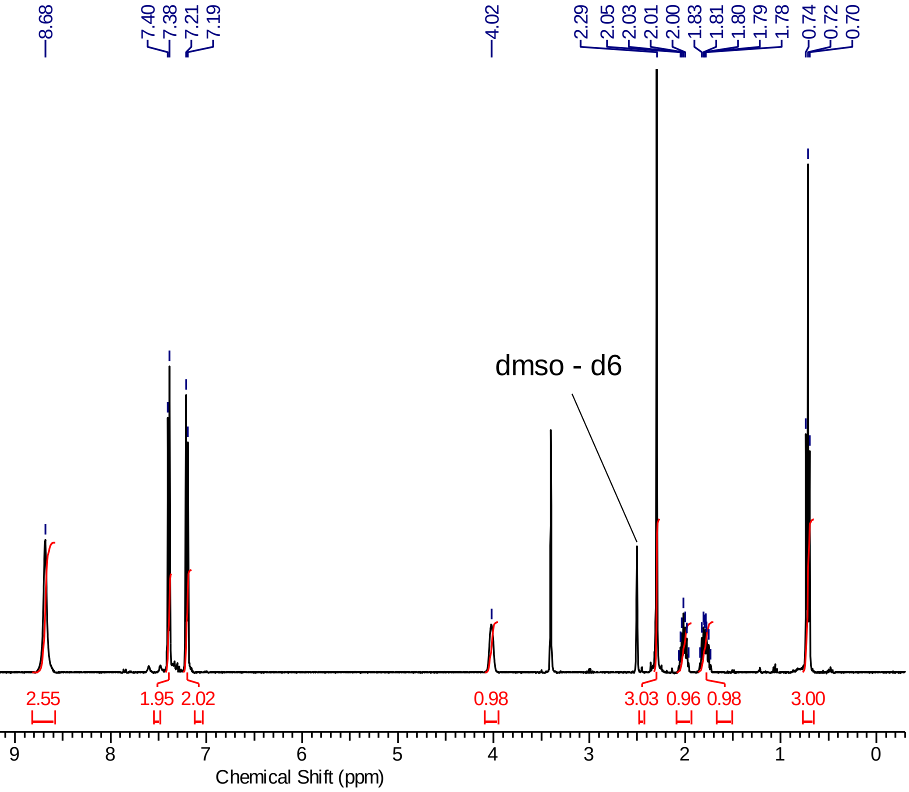
8.68
7.40
7.38
7.21
7.19
4.02
2.29
2.05
2.03
2.01
2.00
1.83
1.81
1.80
1.79
1.78
0.74
0.72
0.70
dmso - d6
2.55
1.95
2.02
0.98
3.03
0.96
0.98
3.00
12
11
10
9
8
7
6
5
4
3
2
1
0
Chemical Shift (ppm)

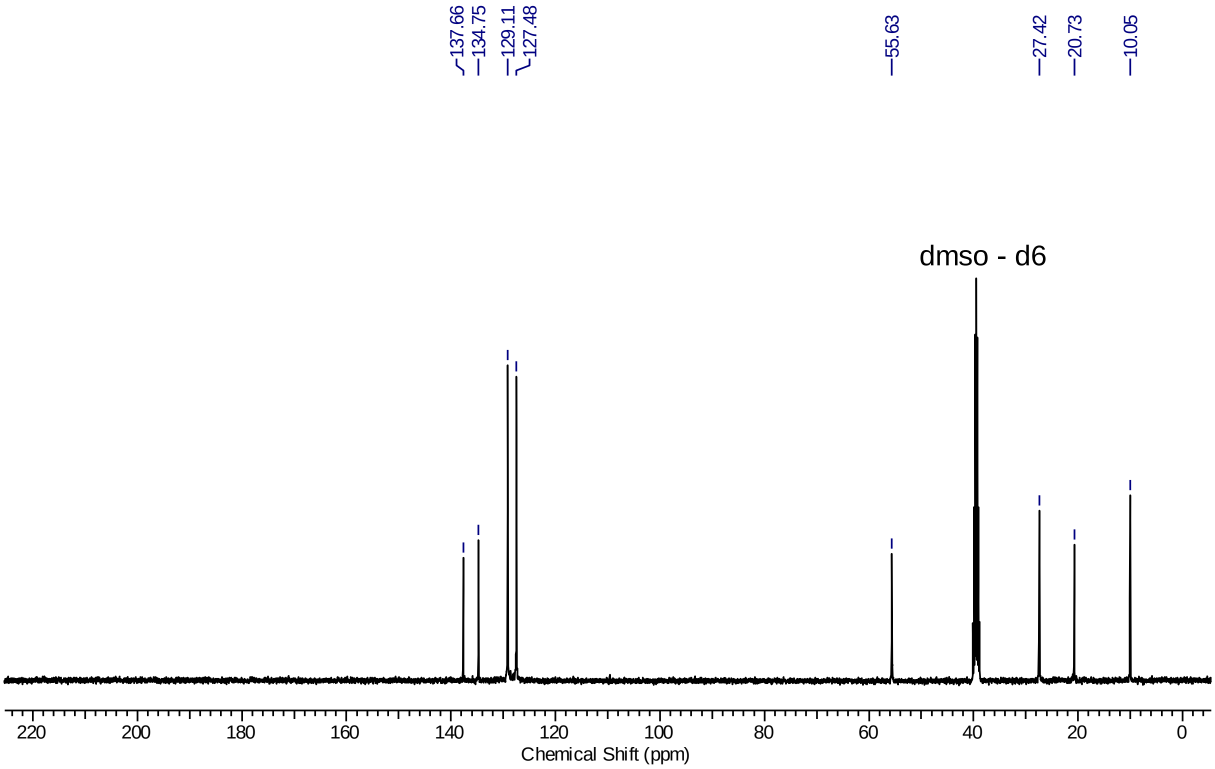
137.66
134.75
129.11
127.48
55.63
27.42
20.73
10.05
dmso - d6
220
200
180
160
140
120
100
80
60
40
20
0
Chemical Shift (ppm)

14:

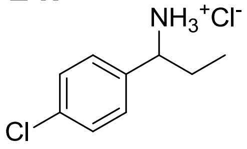

NH3+Cl-
Cl

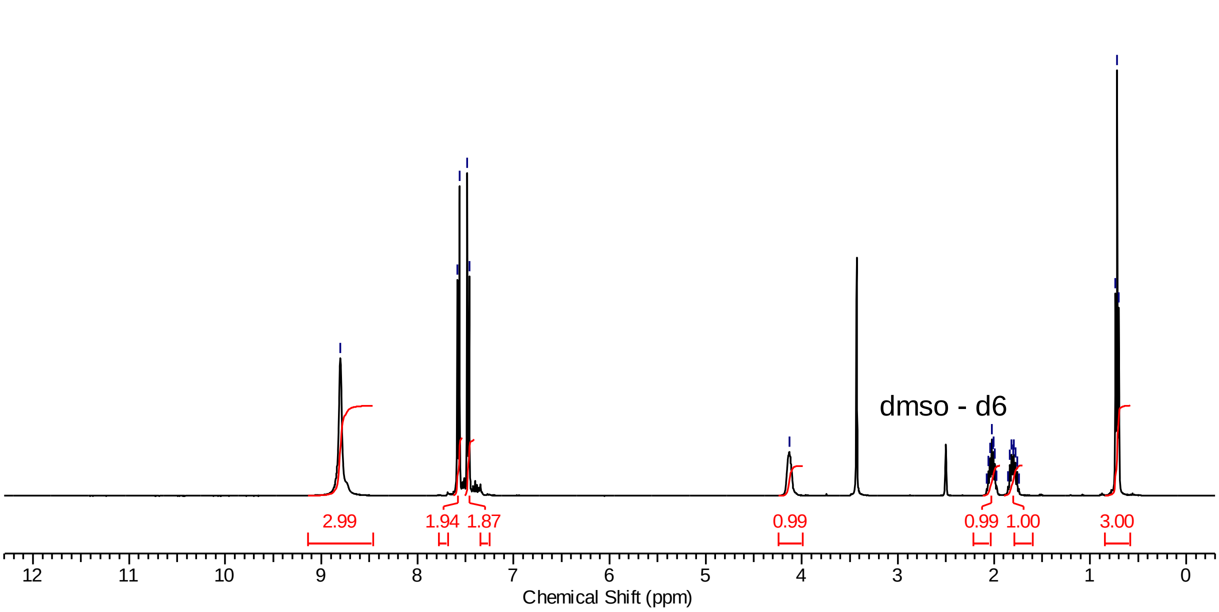

8.80
7.58
7.56
7.48
7.46
4.13
2.06
2.04
2.02
2.01
2.00
1.82
1.80
1.79
1.78
0.74
0.72
0.70
dmso - d6
2.99
1.94
1.87
0.99
0.99
1.00
3.00
Chemical Shift (ppm)

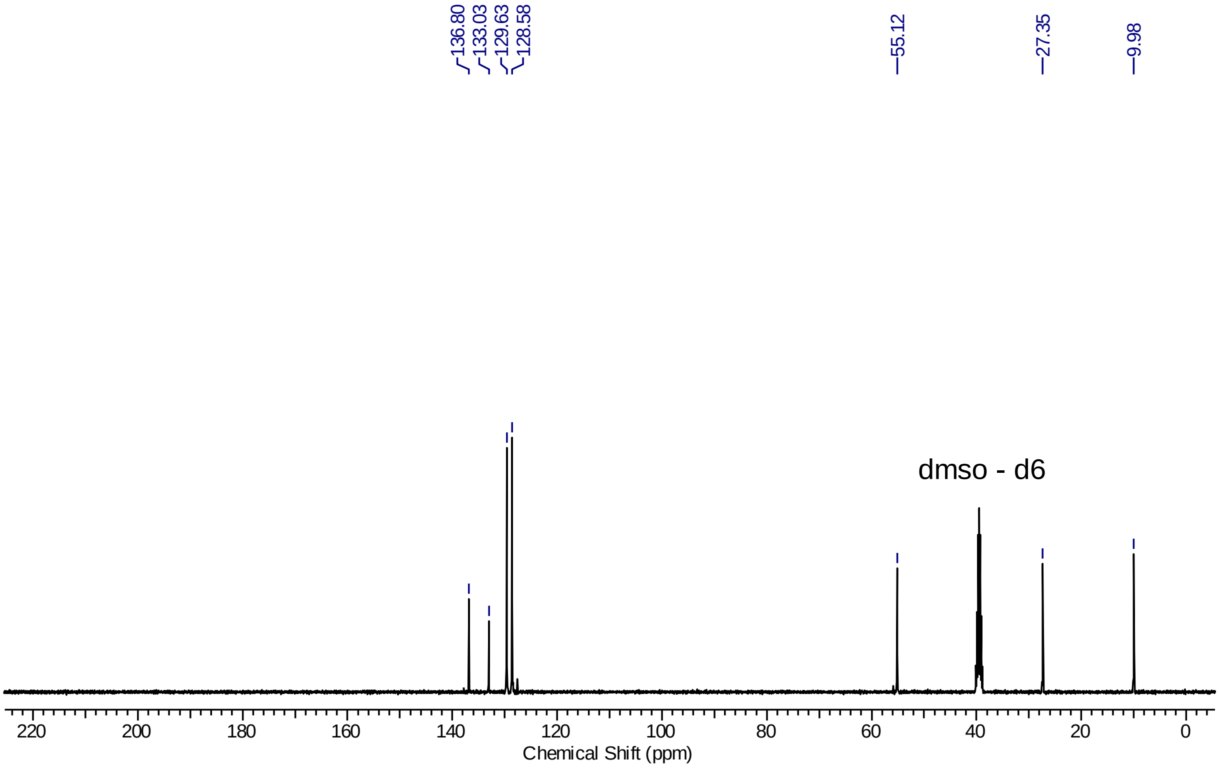

136.80
133.03
129.63
128.58
55.12
27.35
9.98
dmso - d6
Chemical Shift (ppm)

15:

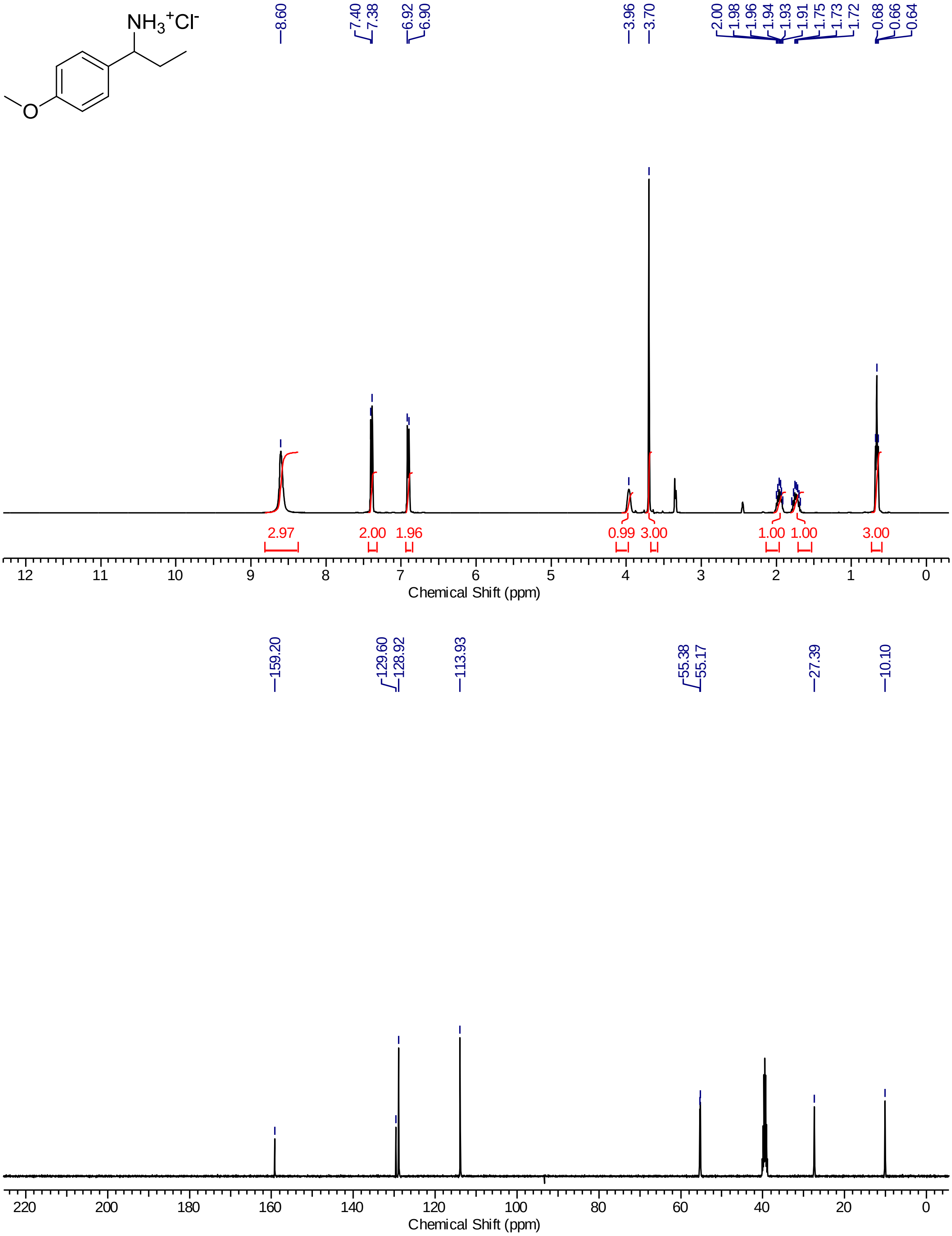

NH3+Cl-
O
8.60
7.40
7.38
6.92
6.90
3.96
3.70
2.00
1.98
1.96
1.94
1.93
1.91
1.75
1.73
1.72
0.68
0.66
0.64
2.97
2.00
1.96
0.99
3.00
1.00
1.00
3.00
12 11 10 9 8 7 6 5 4 3 2 1 0
Chemical Shift (ppm)
159.20
129.60
128.92
113.93
55.38
55.17
27.39
10.10
220 200 180 160 140 120 100 80 60 40 20 0
Chemical Shift (ppm)

16:

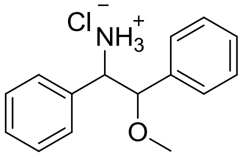
Cl
NH3
O

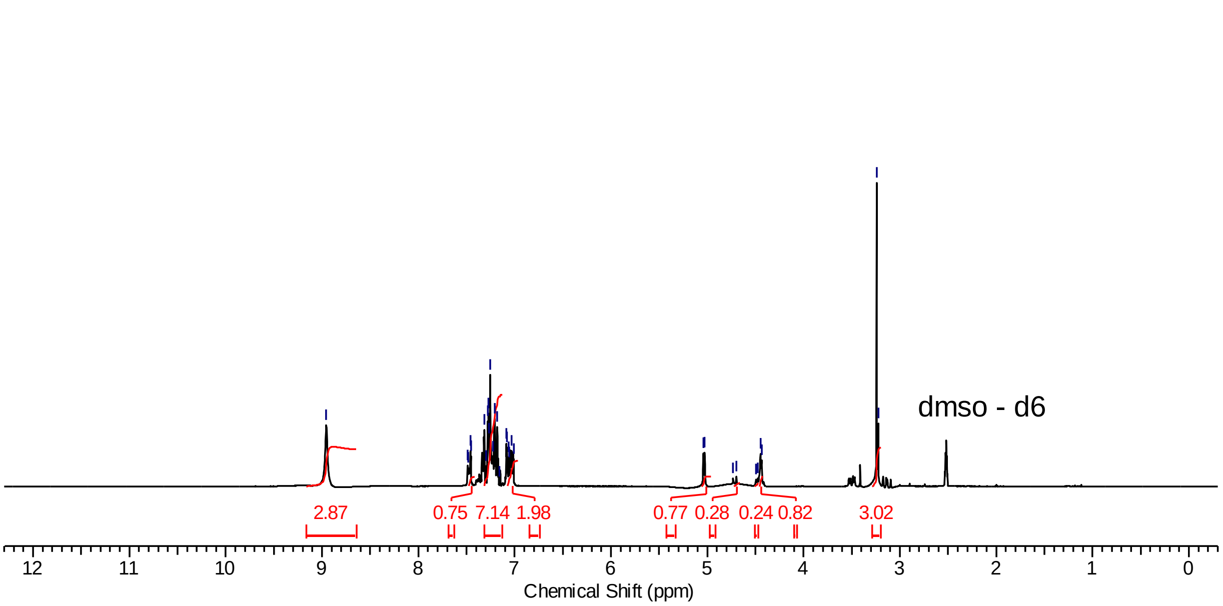
8.96
7.46 7.31 7.28 7.27 7.26 7.25 7.21 7.18 7.09
5.04 5.03 4.73 4.70 4.49 4.48 4.45 4.43
3.24 3.22
dmso - d6
2.87 0.75 7.14 1.98 0.77 0.28 0.24 0.82 3.02
12 11 10 9 8 7 6 5 4 3 2 1 0
Chemical Shift (ppm)

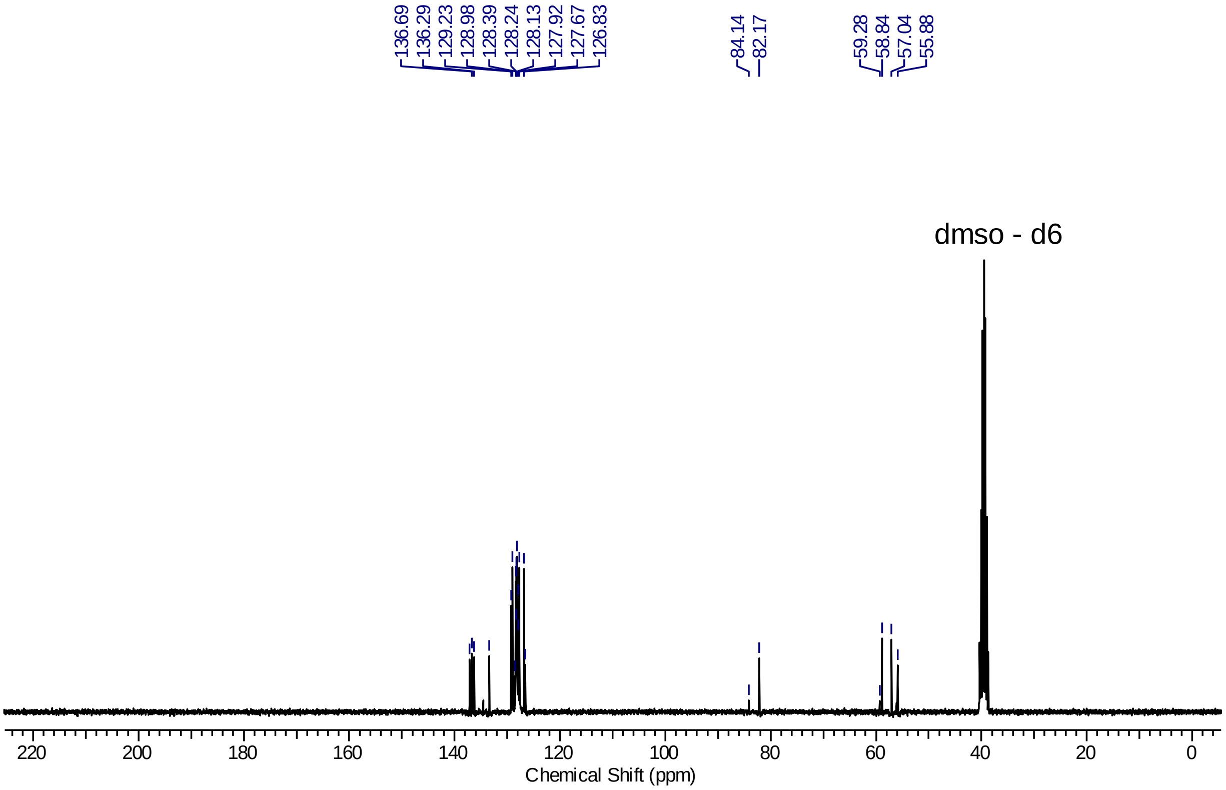
136.69 136.29 129.23 128.98 128.39 128.24 128.13 127.92 127.67 126.83
84.14 82.17
59.28 58.84 57.04 55.88
dmso - d6
220 200 180 160 140 120 100 80 60 40 20 0
Chemical Shift (ppm)

17:

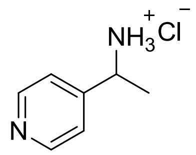
NH3 Cl
N

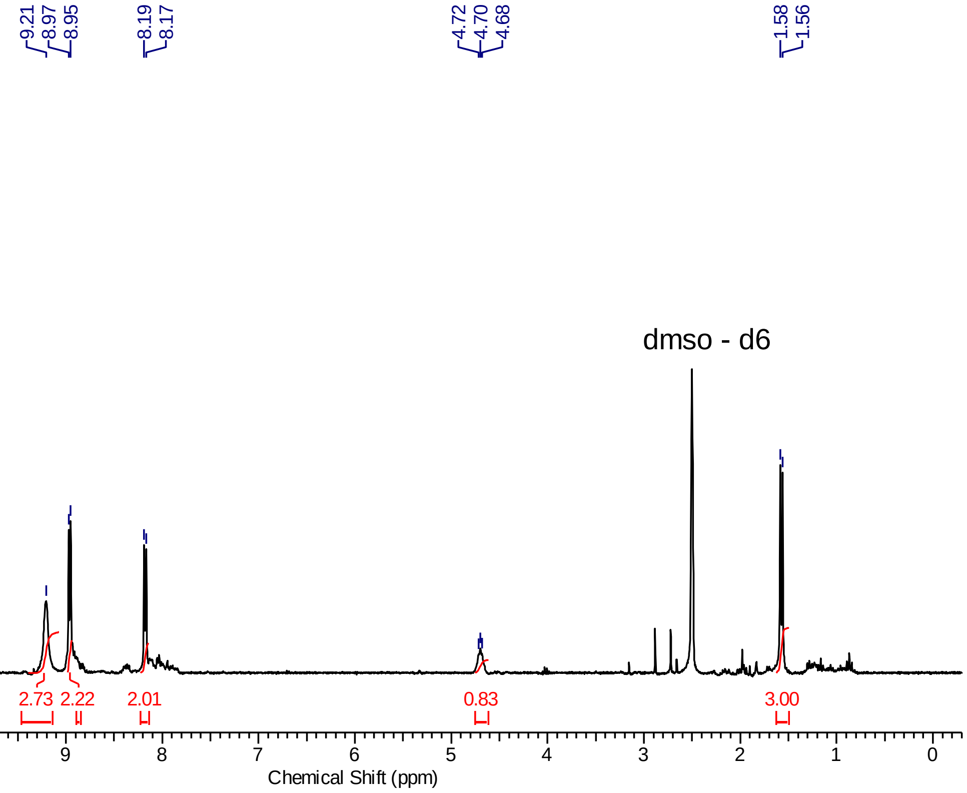
9.21
8.97
8.95
8.19
8.17
4.72
4.70
4.68
1.58
1.56
dmso - d6
2.73
2.22
2.01
0.83
3.00
12
11
10
9
8
7
6
5
4
3
2
1
0
Chemical Shift (ppm)

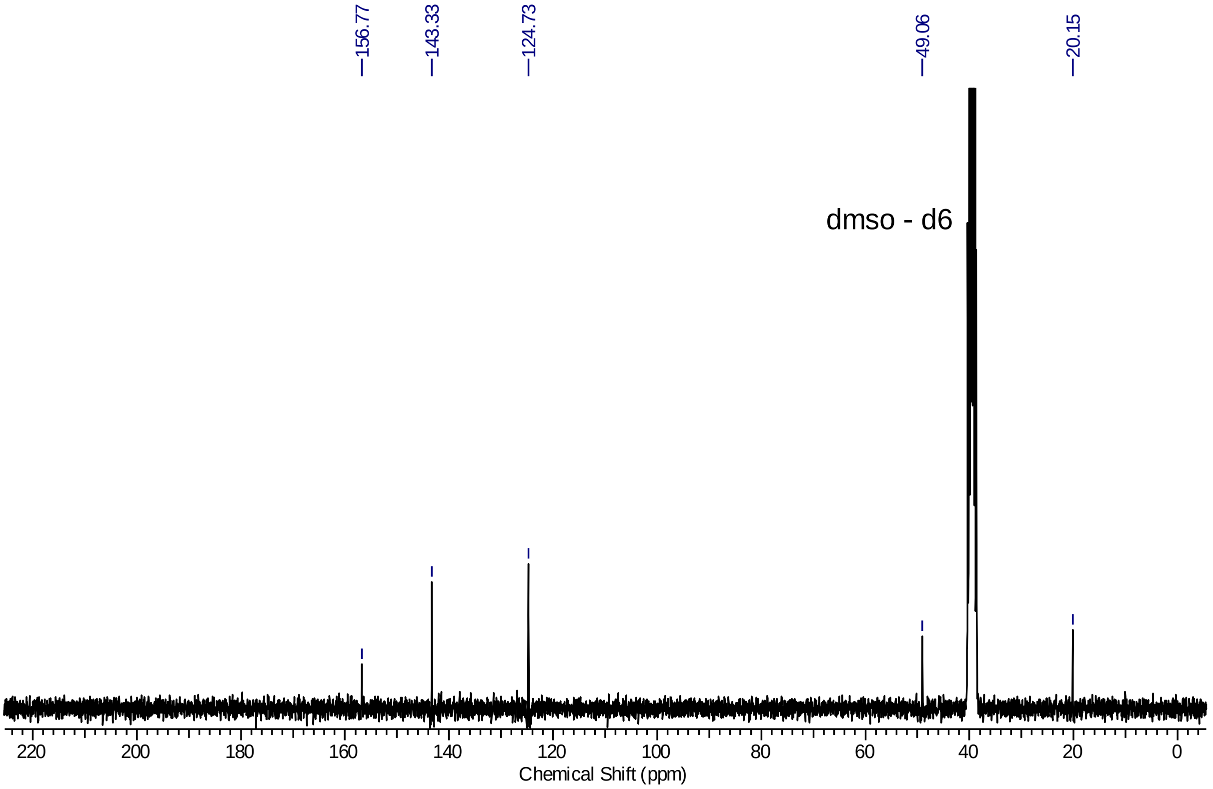
156.77
143.33
124.73
49.06
20.15
dmso - d6
220
200
180
160
140
120
100
80
60
40
20
0
Chemical Shift (ppm)

18:

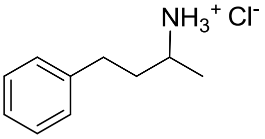

NH3+ Cl-

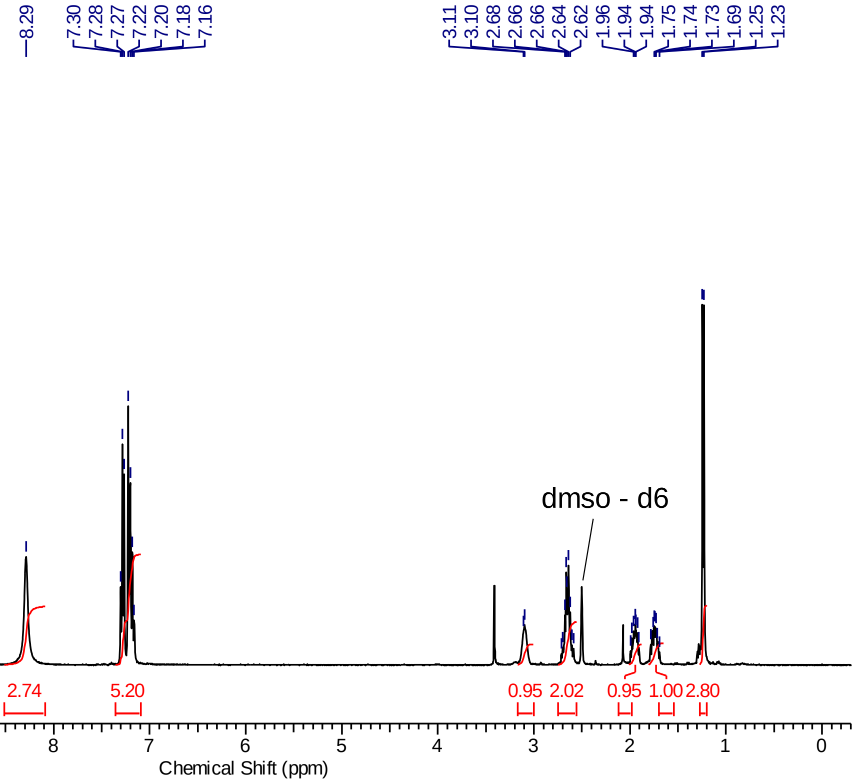

8.29
7.30
7.28
7.27
7.22
7.20
7.18
7.16
3.11
3.10
2.68
2.66
2.66
2.64
2.62
1.96
1.94
1.94
1.75
1.74
1.73
1.69
1.25
1.23
dmso - d6
2.74
5.20
0.95
2.02
0.95
1.00
2.80
12
11
10
9
8
7
6
5
4
3
2
1
0
Chemical Shift (ppm)

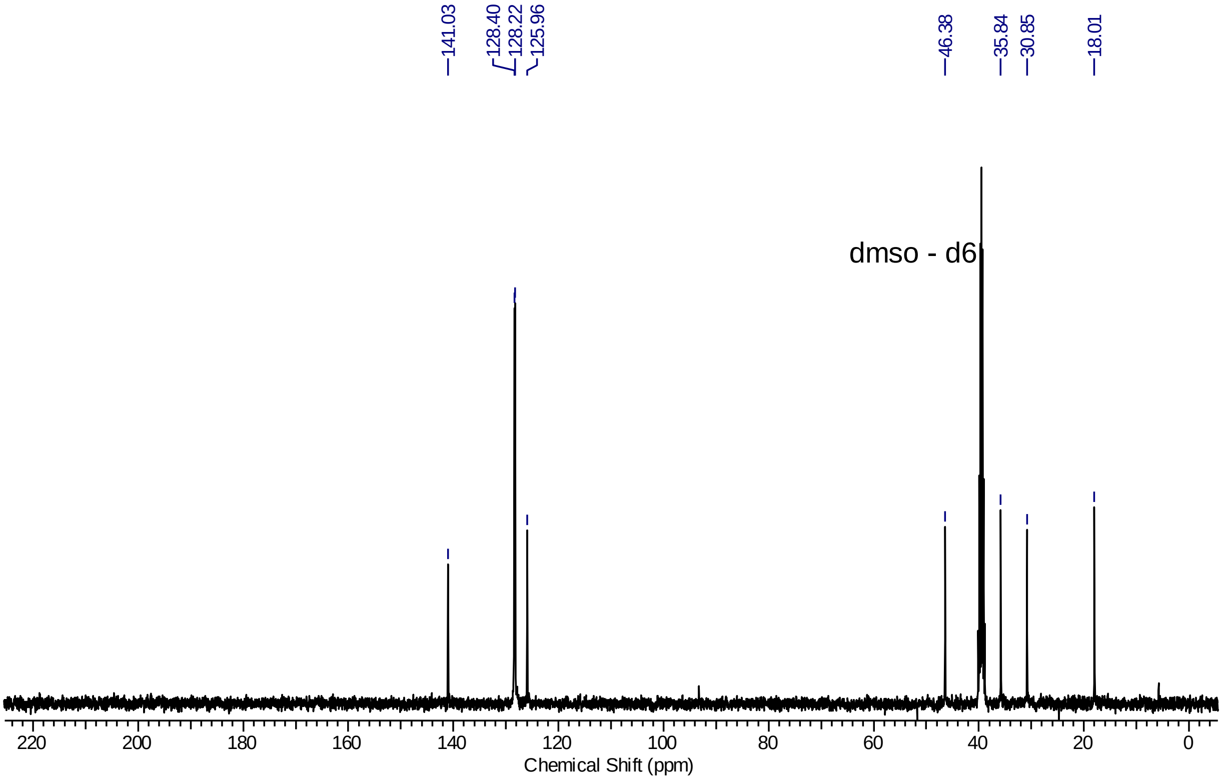

141.03
128.40
128.22
125.96
46.38
35.84
30.85
18.01
dmso - d6
220
200
180
160
140
120
100
80
60
40
20
0
Chemical Shift (ppm)

19:

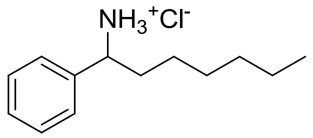
NH3+Cl-

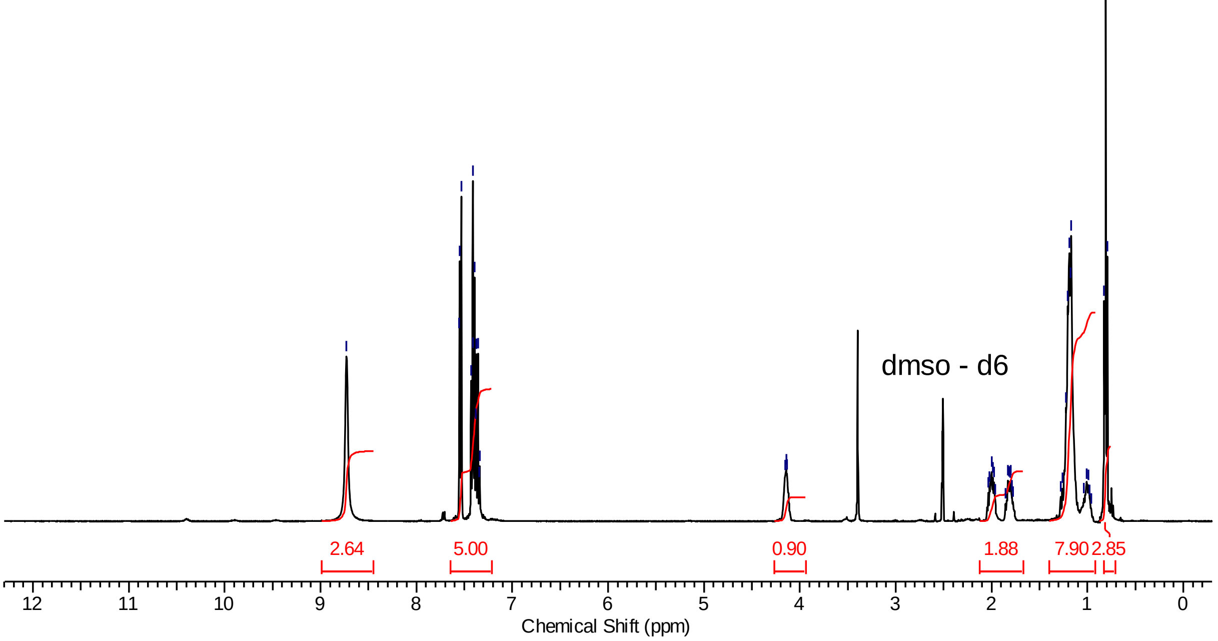
8.73
7.55
7.55
7.53
7.41
7.41
7.39
7.38
7.36
4.15
4.14
4.13
2.00
1.99
1.83
1.81
1.80
1.22
1.21
1.19
1.18
1.17
0.83
0.81
0.79
dmso - d6
2.64
5.00
0.90
1.88
7.90
2.85
12
11
10
9
8
7
6
5
4
3
2
1
0
Chemical Shift (ppm)

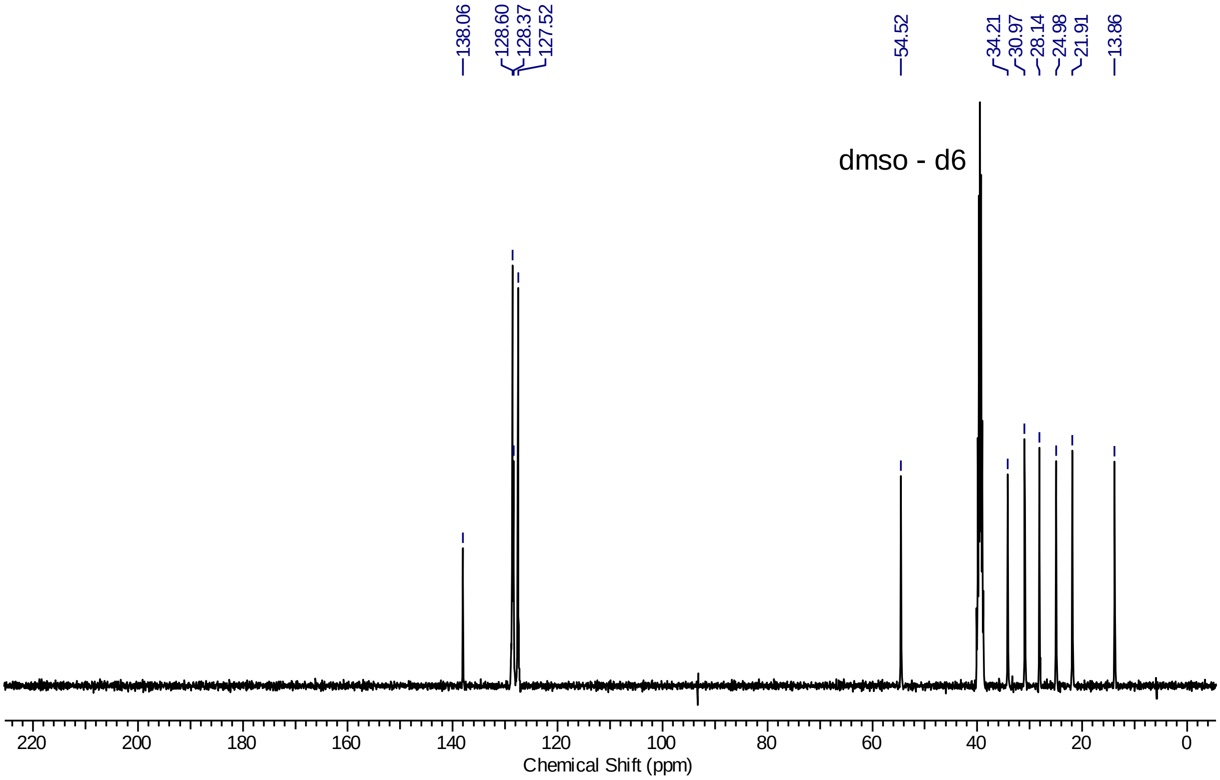
138.06
128.60
128.37
127.52
54.52
34.21
30.97
28.14
24.98
21.91
13.86
dmso - d6
220
200
180
160
140
120
100
80
60
40
20
0
Chemical Shift (ppm)

20:

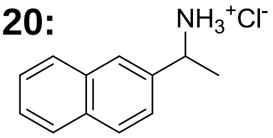
$NH_3^+Cl^-$

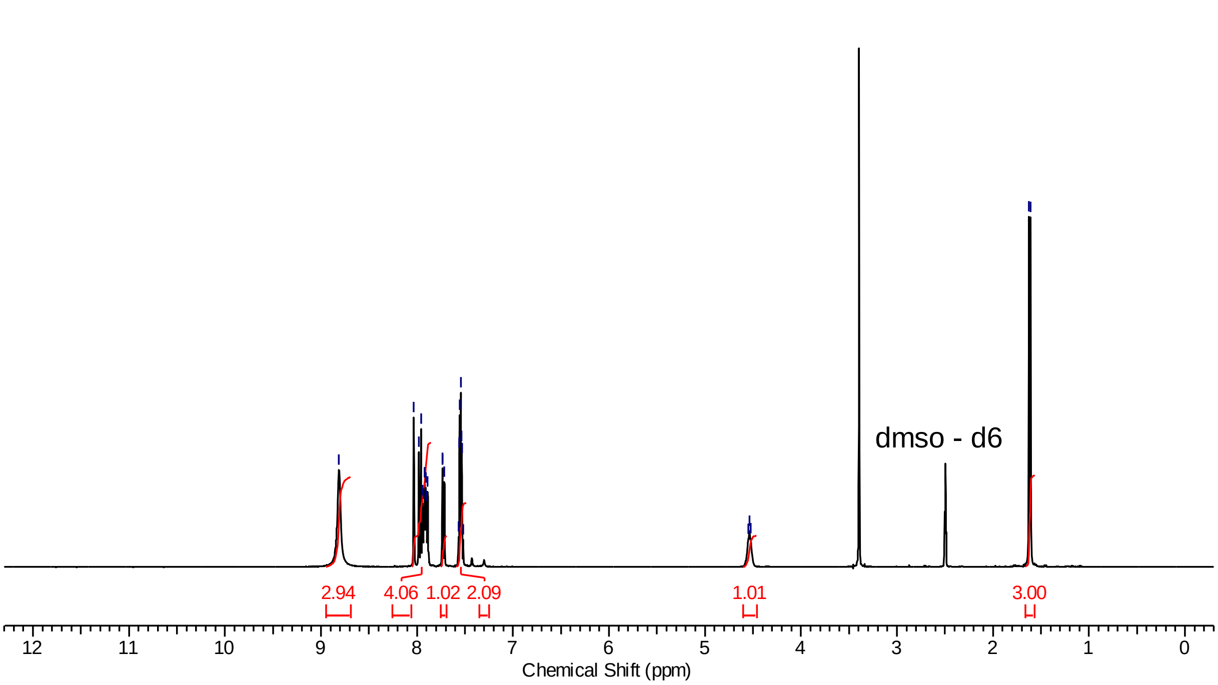
8.81
8.03
7.98
7.96
7.93
7.72
7.56
7.55
7.54
7.53
7.53
4.55
4.54
4.52
1.62
1.61
dmso - d6
2.94
4.06
1.02
2.09
1.01
3.00
12
11
10
9
8
7
6
5
4
3
2
1
0
Chemical Shift (ppm)

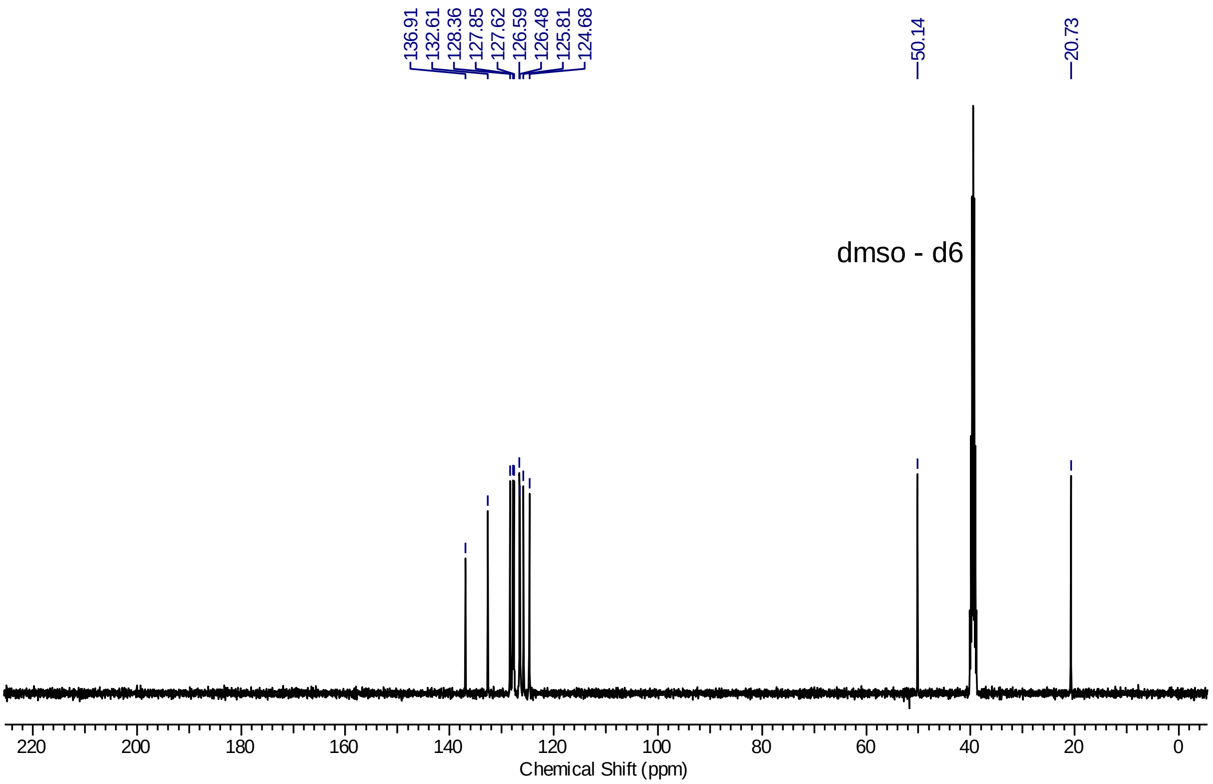
136.91
132.61
128.36
127.85
127.62
126.59
126.48
125.81
124.68
50.14
20.73
dmso - d6
220
200
180
160
140
120
100
80
60
40
20
0
Chemical Shift (ppm)

21:

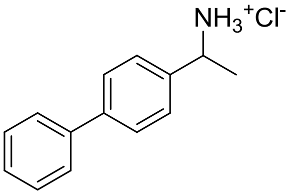
NH3+Cl-

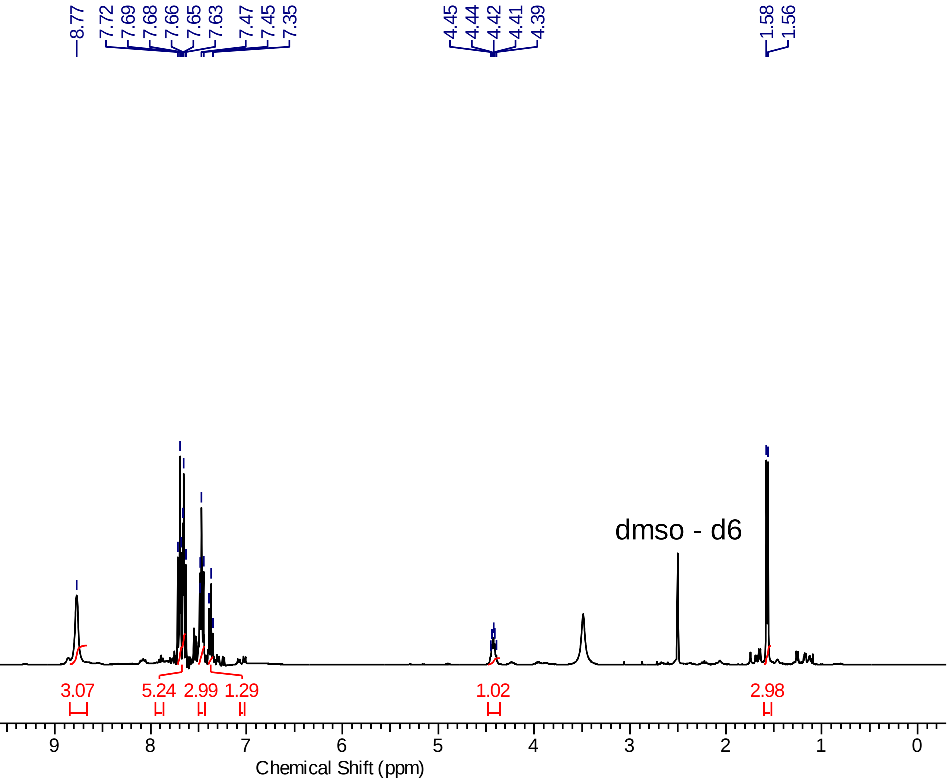
8.77
7.72
7.69
7.68
7.66
7.65
7.63
7.47
7.45
7.35
4.45
4.44
4.42
4.41
4.39
1.58
1.56
dmso - d6
3.07
5.24
2.99
1.29
1.02
2.98
12
11
10
9
8
7
6
5
4
3
2
1
0
Chemical Shift (ppm)

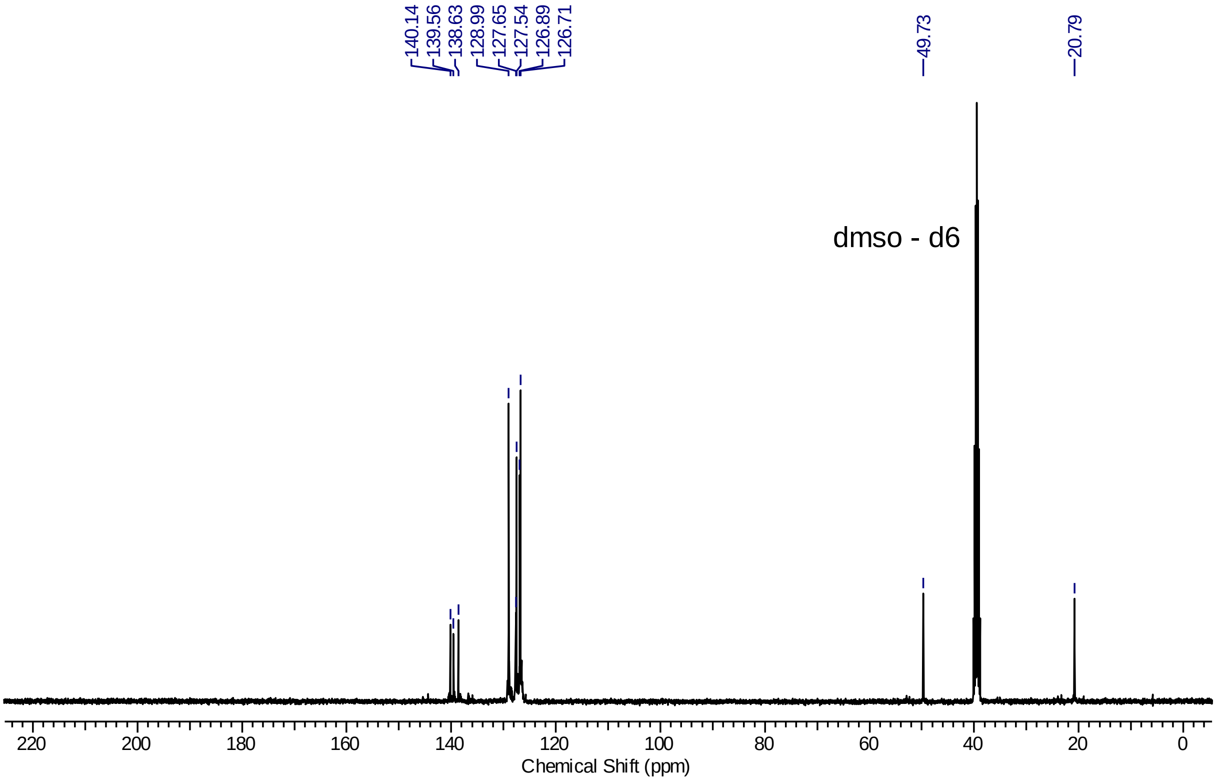
140.14
139.56
138.63
128.99
127.65
127.54
126.89
126.71
49.73
20.79
dmso - d6
220
200
180
160
140
120
100
80
60
40
20
0
Chemical Shift (ppm)

22:

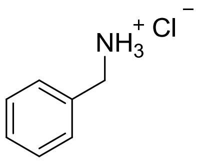
NH3+ Cl−

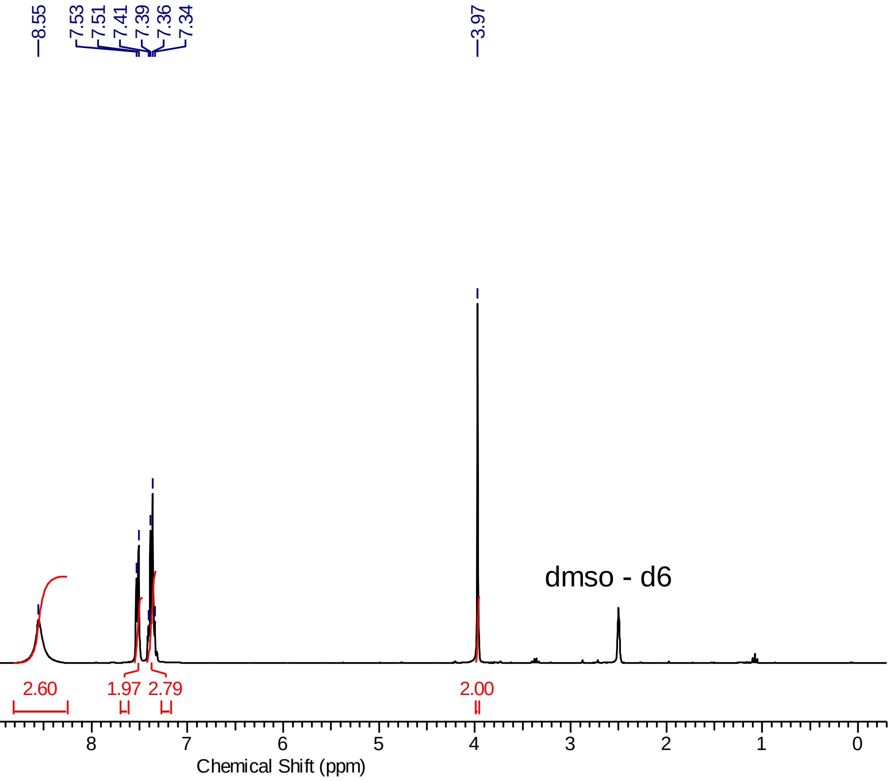
8.55
7.53
7.51
7.41
7.39
7.36
7.34
3.97
dmso - d6
2.60
1.97
2.79
2.00
12
11
10
9
8
7
6
5
4
3
2
1
0
Chemical Shift (ppm)

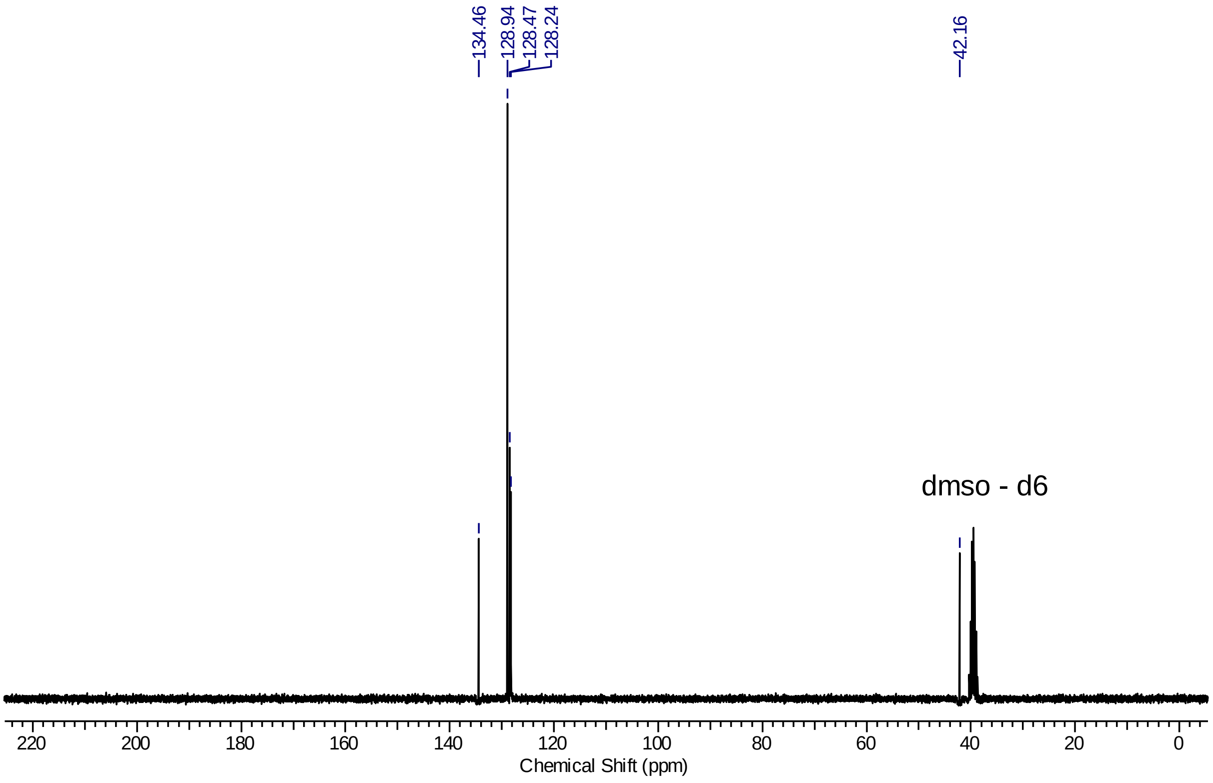
134.46
128.94
128.47
128.24
42.16
dmso - d6
220
200
180
160
140
120
100
80
60
40
20
0
Chemical Shift (ppm)

23:

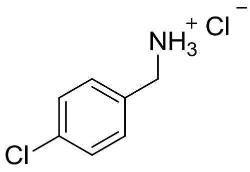

NH_3^+ Cl^-
Cl

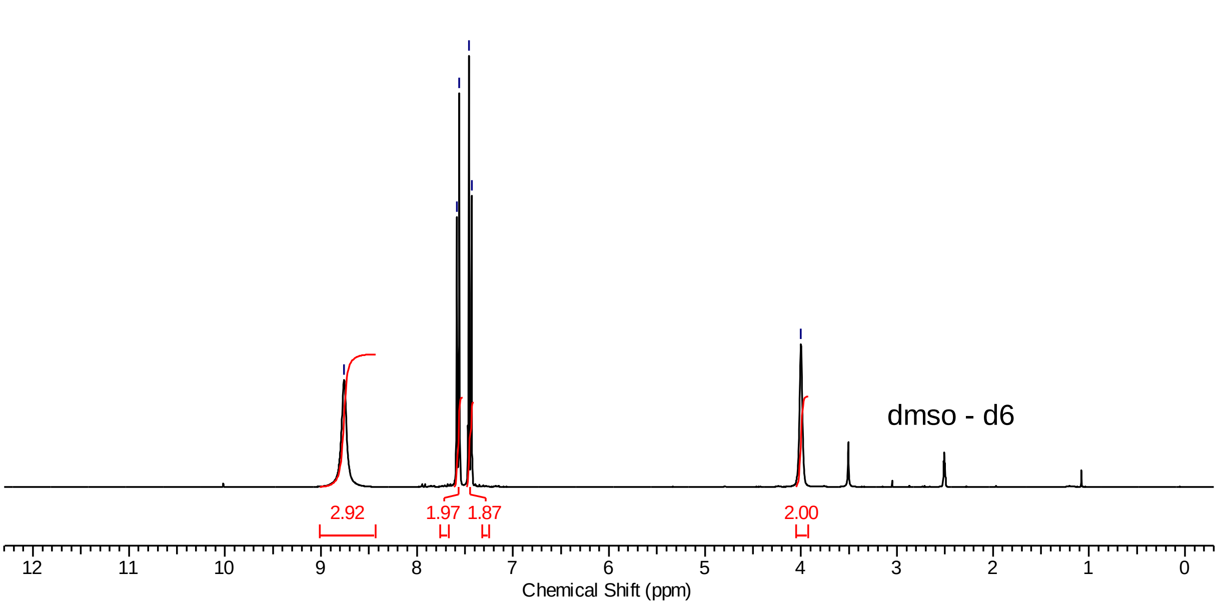

8.76
7.59
7.56
7.46
7.43
4.00
dmso - d6
2.92
1.97
1.87
2.00
12 11 10 9 8 7 6 5 4 3 2 1 0
Chemical Shift (ppm)

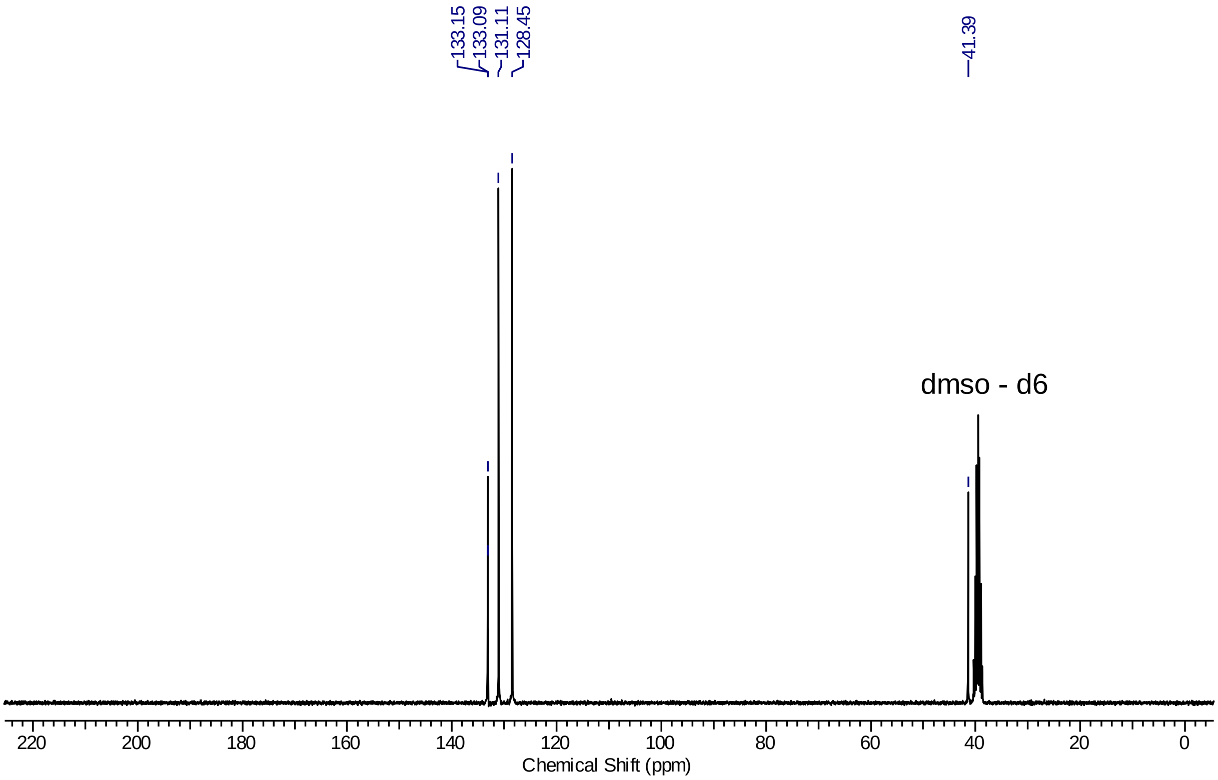

133.15
133.09
131.11
128.45
41.39
dmso - d6
220 200 180 160 140 120 100 80 60 40 20 0
Chemical Shift (ppm)

24:

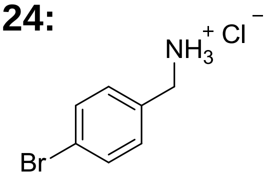

NH3+ Cl−
Br

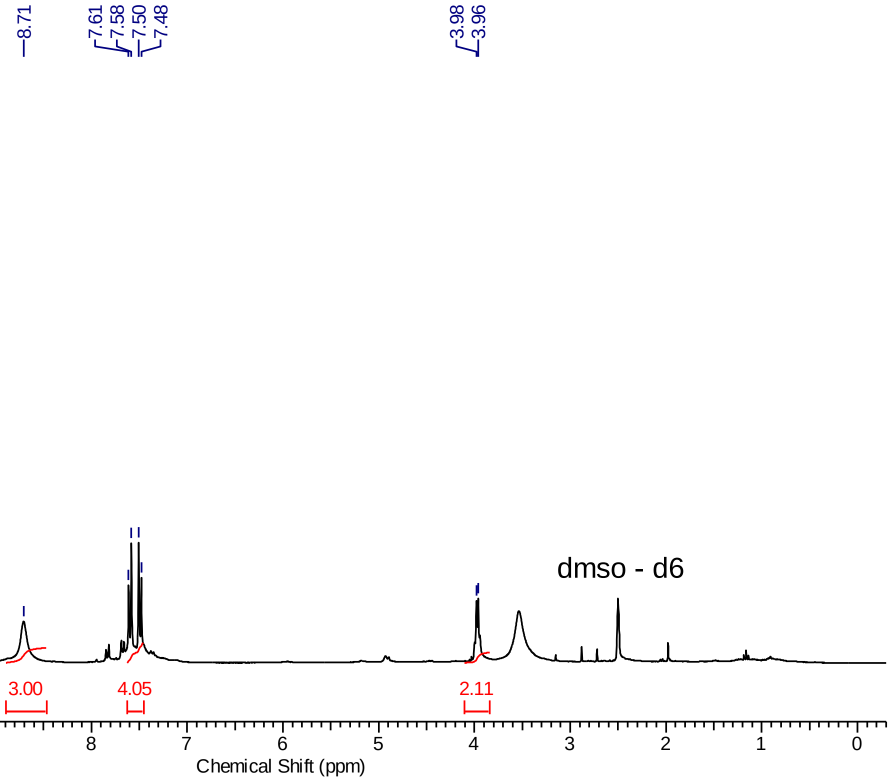

8.71
7.61
7.58
7.50
7.48
3.98
3.96
dmso - d6
3.00
4.05
2.11
12 11 10 9 8 7 6 5 4 3 2 1 0
Chemical Shift (ppm)

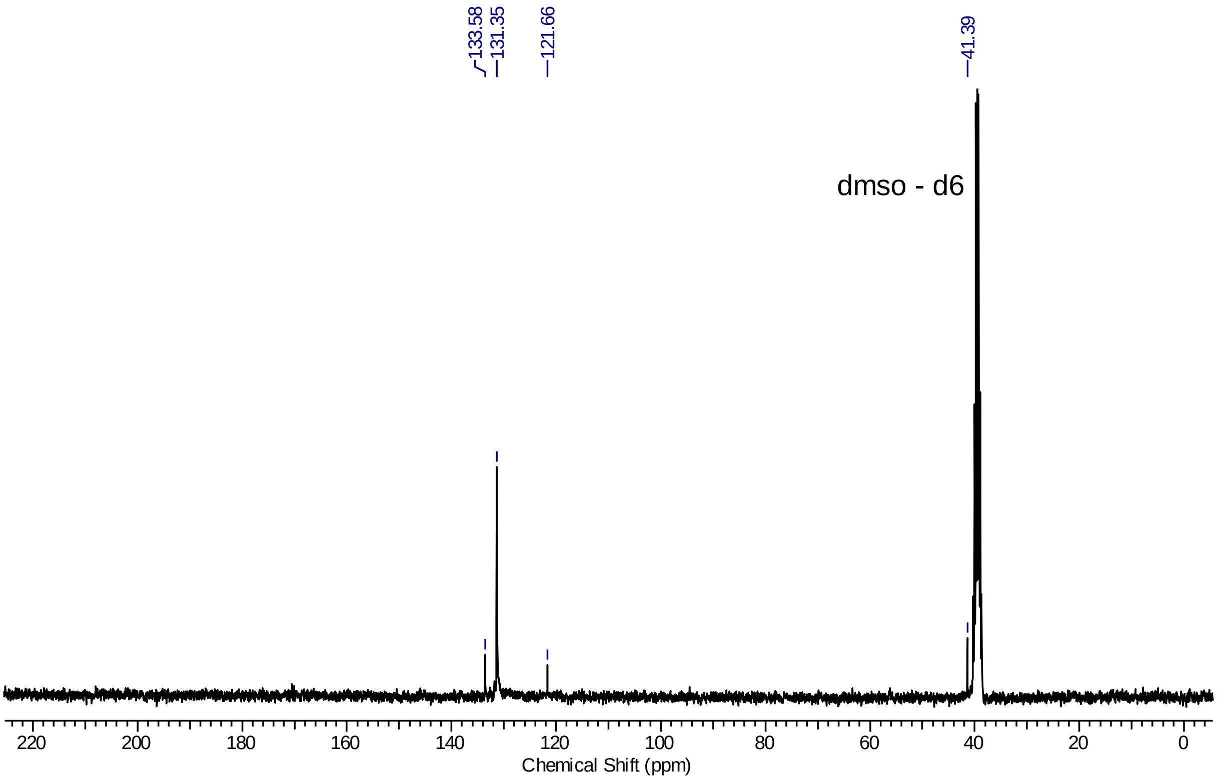

133.58
131.35
121.66
41.39
dmso - d6
220 200 180 160 140 120 100 80 60 40 20 0
Chemical Shift (ppm)

25:

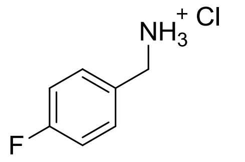
NH3 + Cl −
F

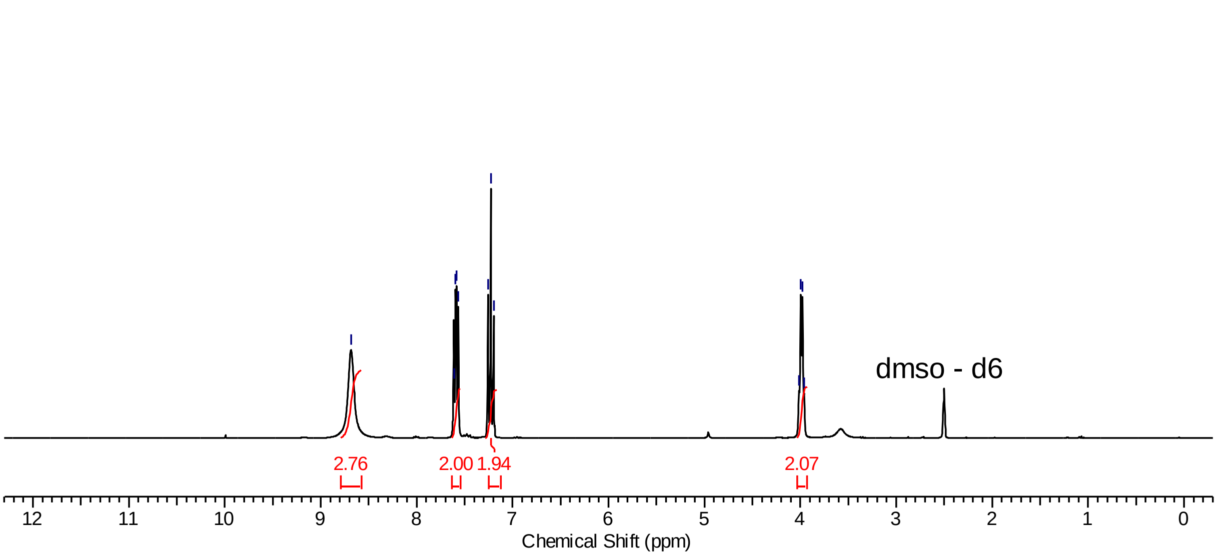
8.68
7.61
7.59
7.58
7.56
7.26
7.23
7.20
4.01
4.00
3.98
3.96
dmso - d6
2.76
2.00
1.94
2.07
12
11
10
9
8
7
6
5
4
3
2
1
0
Chemical Shift (ppm)

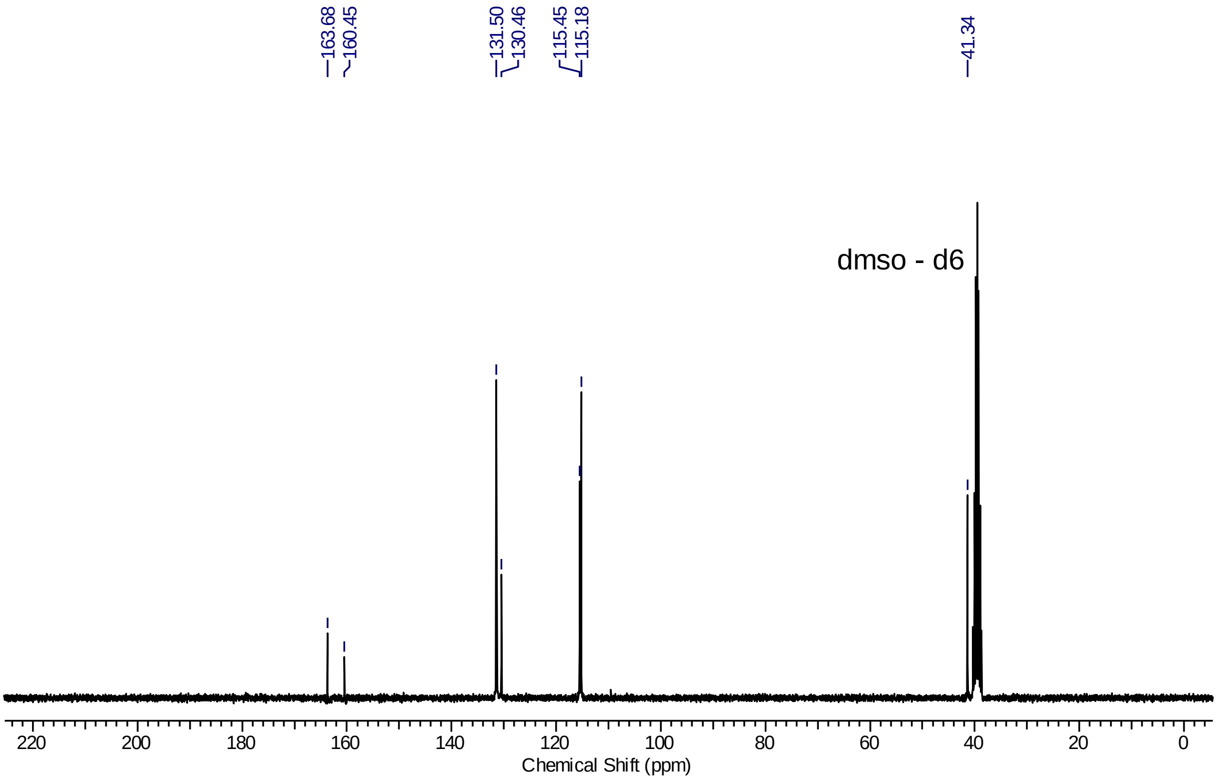
163.68
160.45
131.50
130.46
115.45
115.18
41.34
dmso - d6
220
200
180
160
140
120
100
80
60
40
20
0
Chemical Shift (ppm)

26:

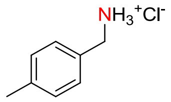
NH3+Cl-

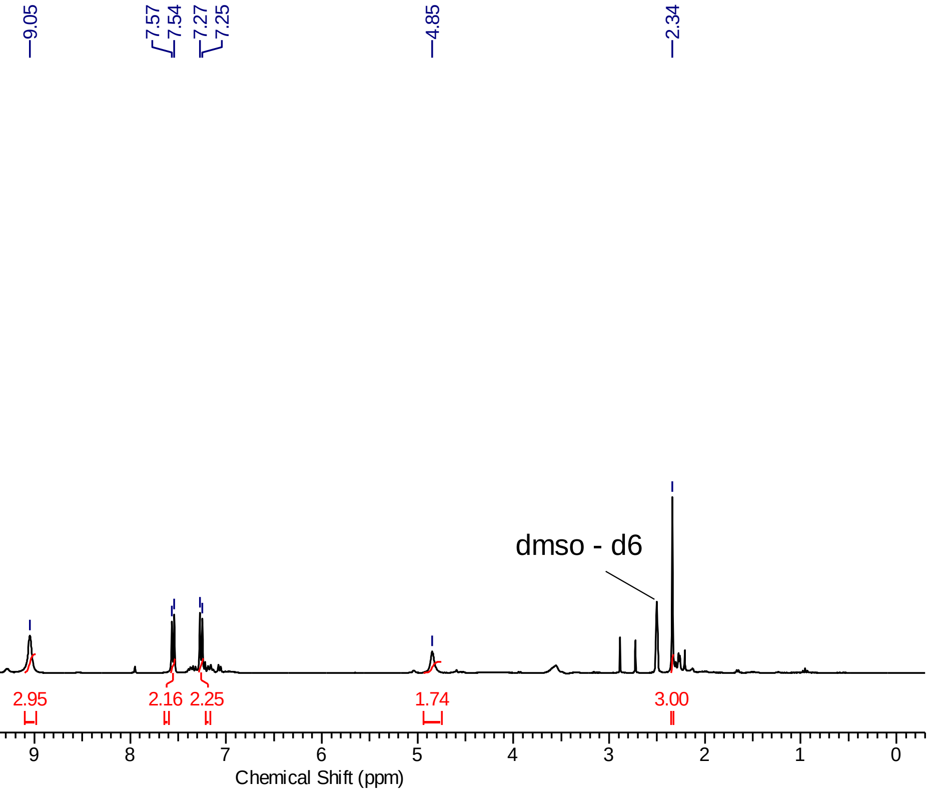
9.05
7.57
7.54
7.27
7.25
4.85
2.34
dmso - d6
2.95
2.16
2.25
1.74
3.00
12
11
10
9
8
7
6
5
4
3
2
1
0
Chemical Shift (ppm)

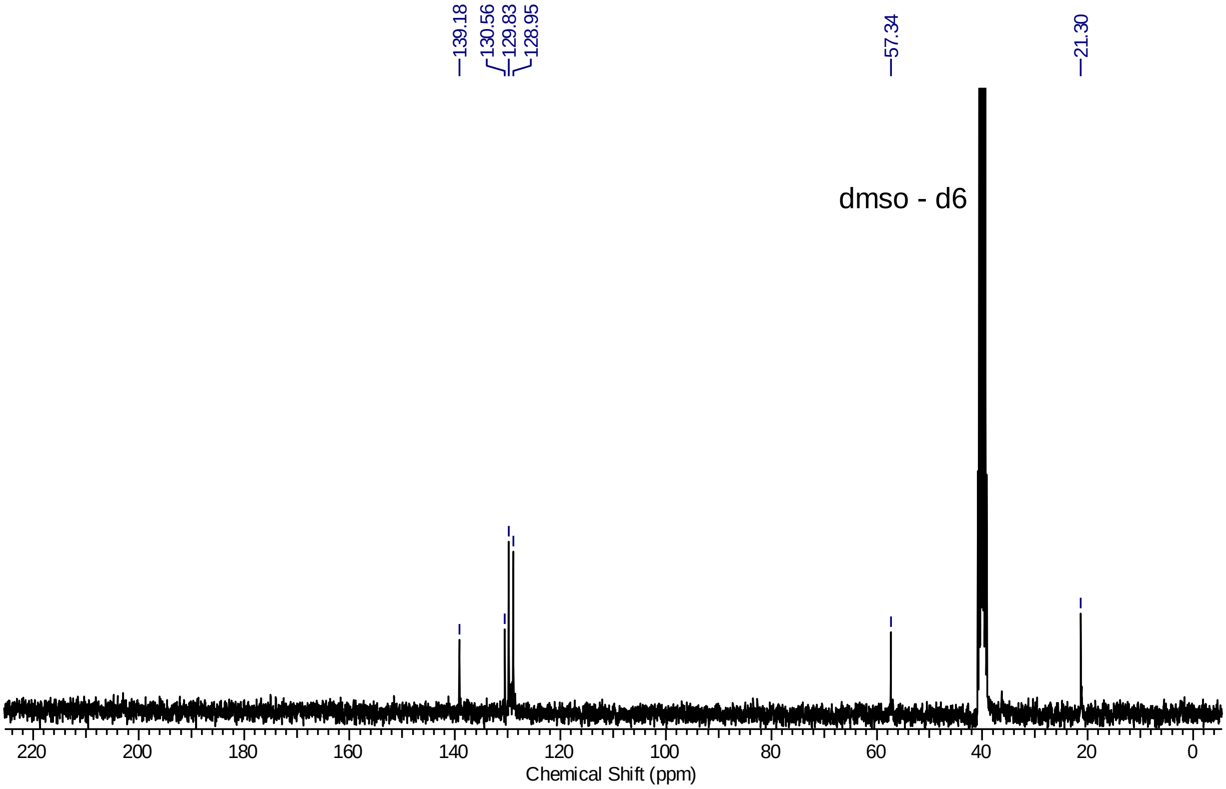
139.18
130.56
129.83
128.95
57.34
21.30
dmso - d6
220
200
180
160
140
120
100
80
60
40
20
0
Chemical Shift (ppm)

27:

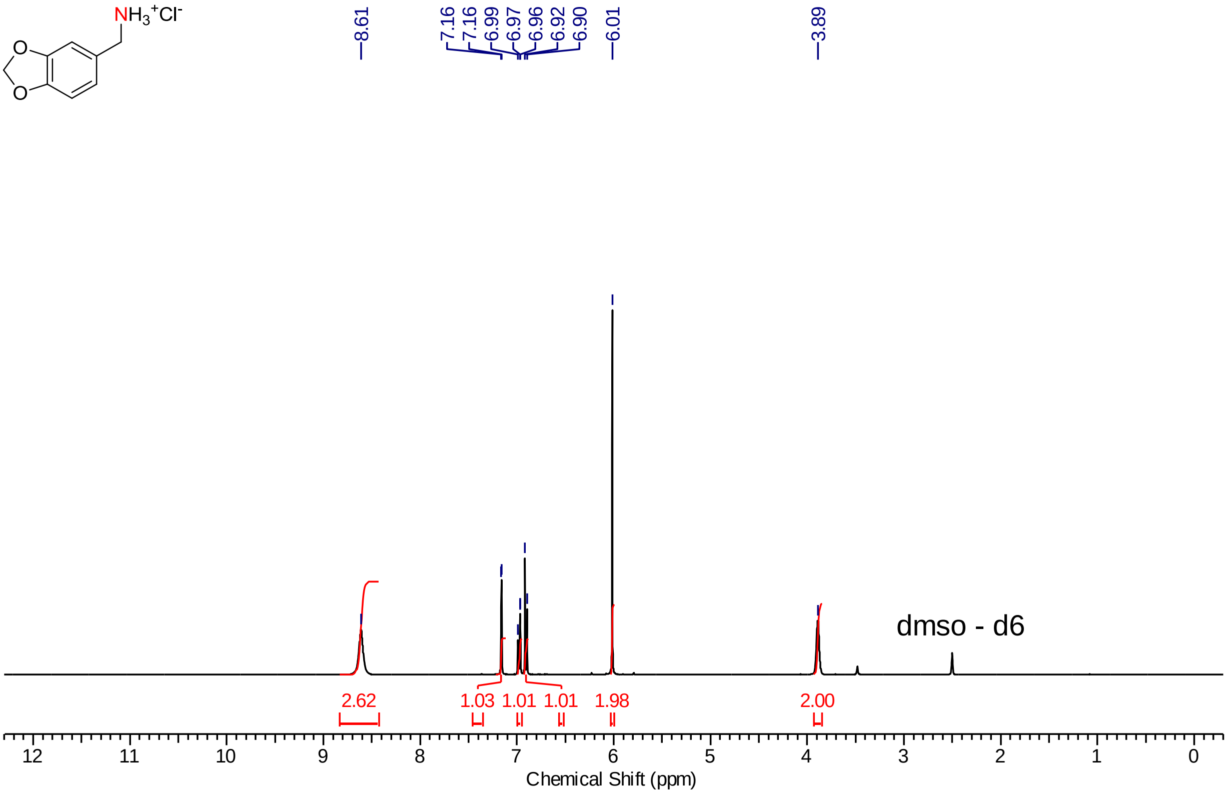

NH3+Cl-
O
O
8.61
7.16
7.16
6.99
6.97
6.96
6.92
6.90
6.01
3.89
dmso - d6
2.62
1.03
1.01
1.01
1.98
2.00
12 11 10 9 8 7 6 5 4 3 2 1 0
Chemical Shift (ppm)

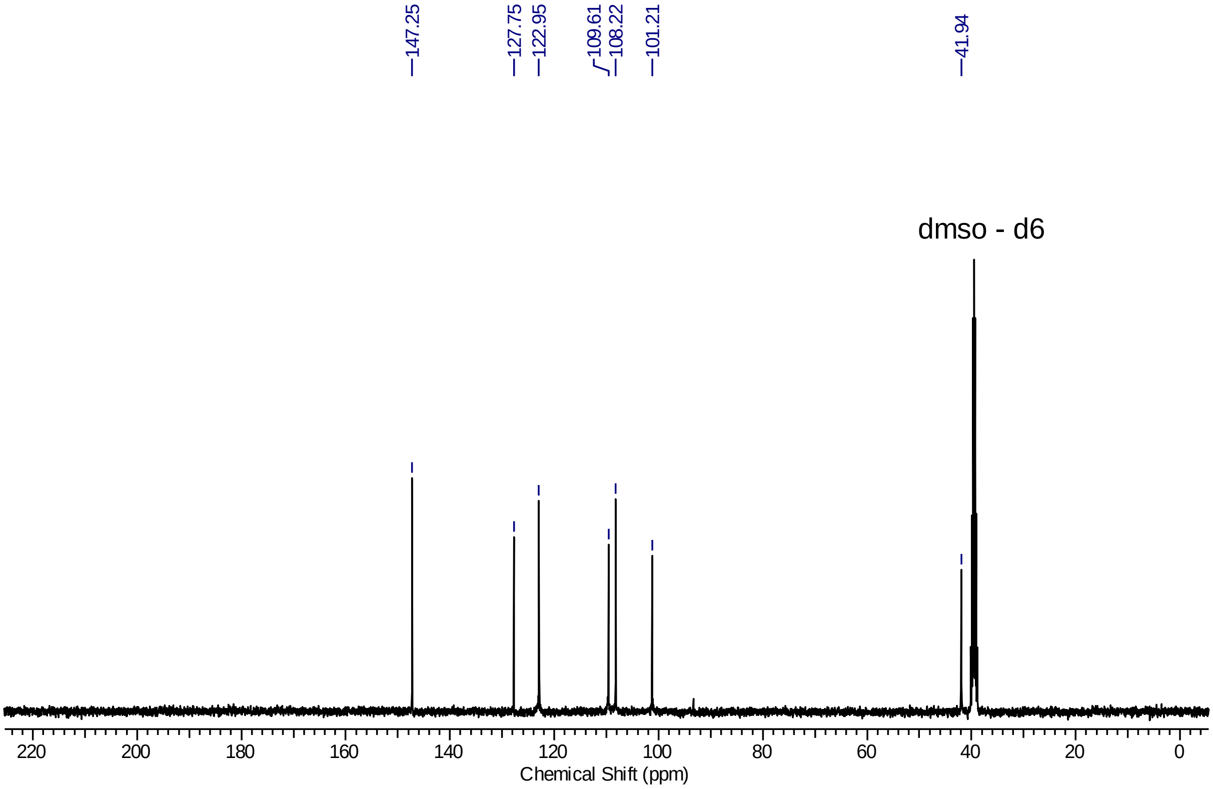

147.25
127.75
122.95
109.61
108.22
101.21
41.94
dmso - d6
220 200 180 160 140 120 100 80 60 40 20 0
Chemical Shift (ppm)

28:

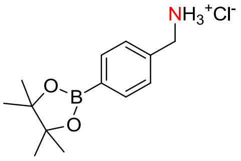
NH3+Cl-
O
B
O

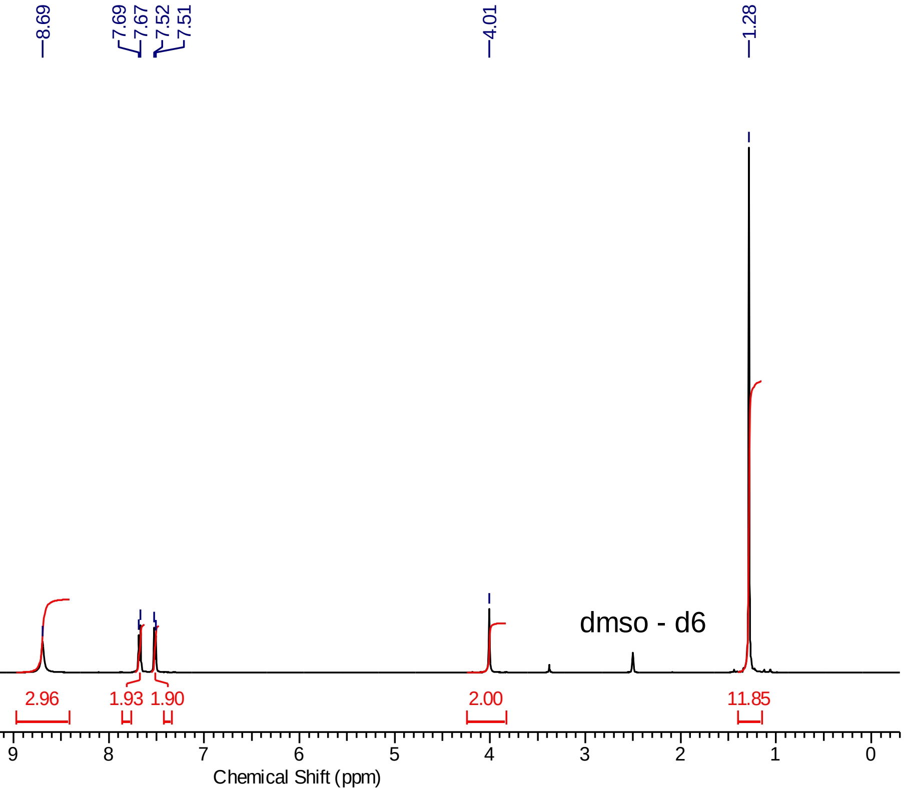
8.69
7.69
7.67
7.52
7.51
4.01
1.28
dmso - d6
2.96
1.93
1.90
2.00
11.85
12 11 10 9 8 7 6 5 4 3 2 1 0
Chemical Shift (ppm)

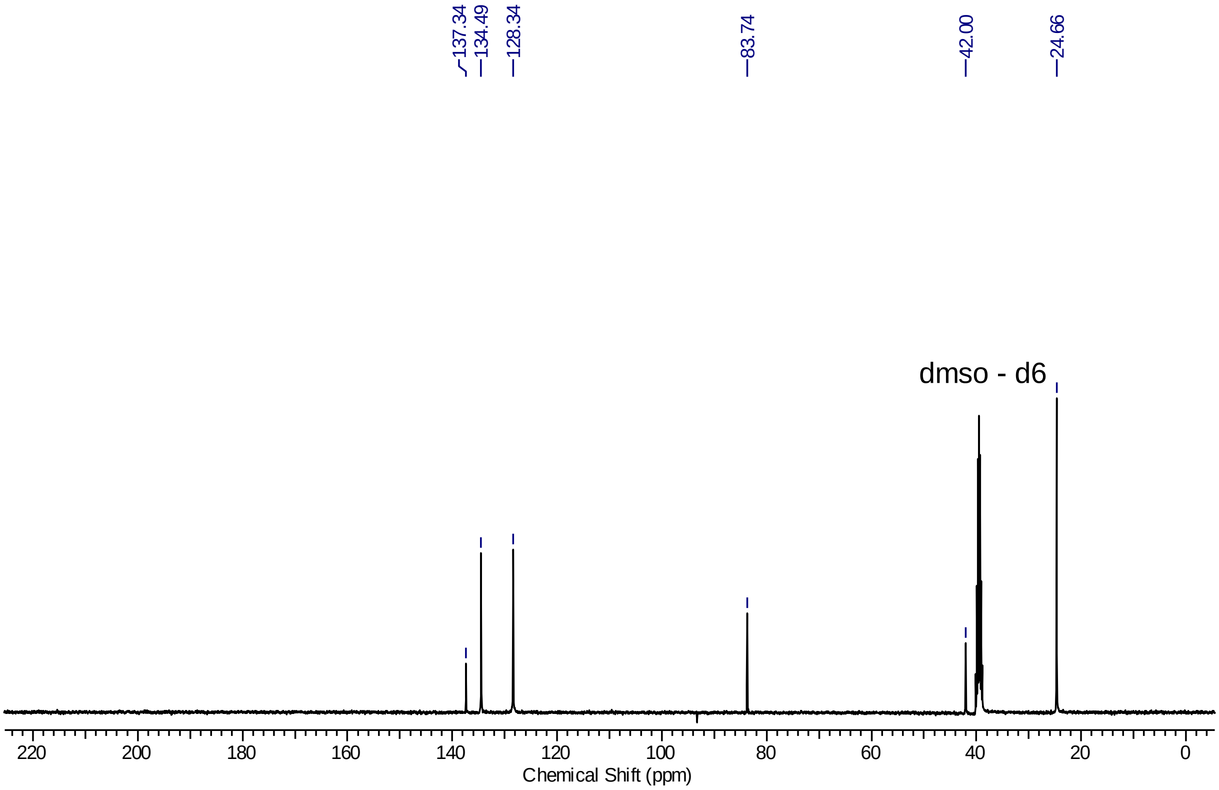
137.34
134.49
128.34
83.74
42.00
24.66
dmso - d6
220 200 180 160 140 120 100 80 60 40 20 0
Chemical Shift (ppm)

29:

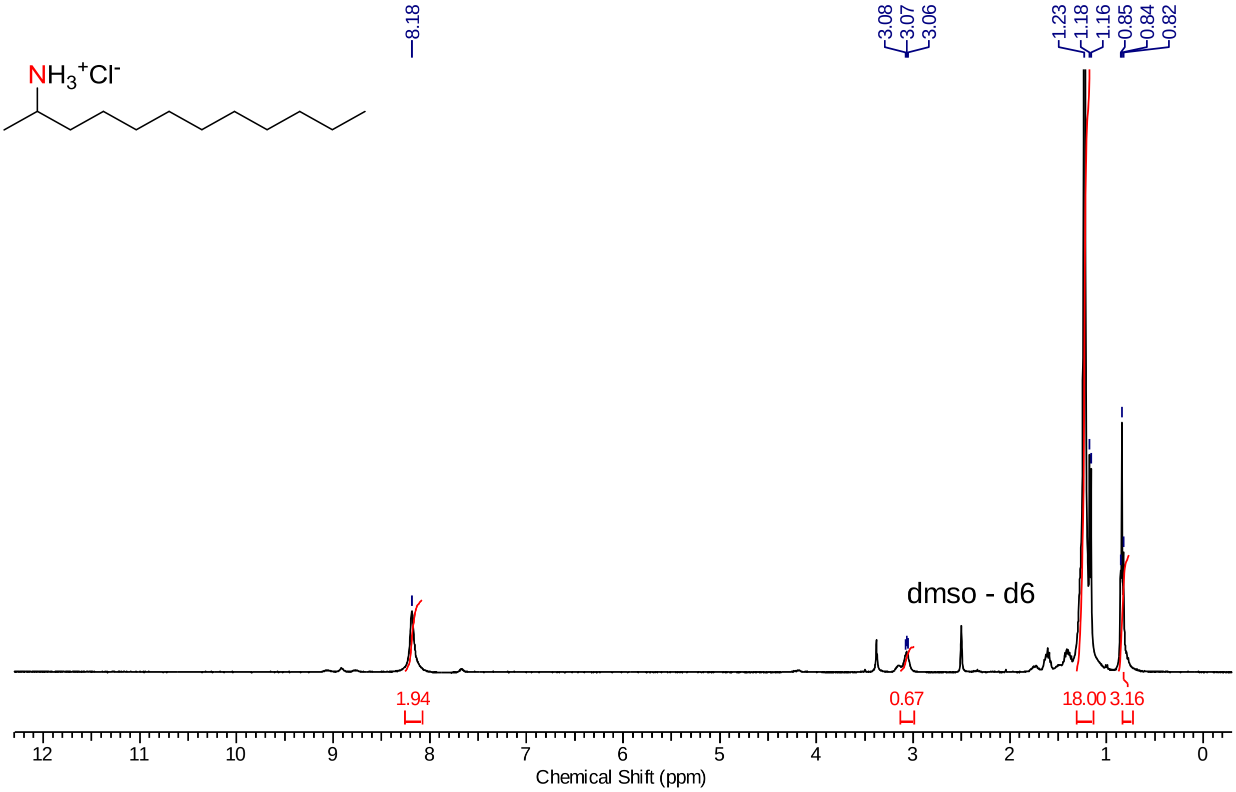

8.18
3.08
3.07
3.06
1.23
1.18
1.16
0.85
0.84
0.82
dmso - d6
1.94
0.67
18.00
3.16
12
11
10
9
8
7
6
5
4
3
2
1
0
Chemical Shift (ppm)

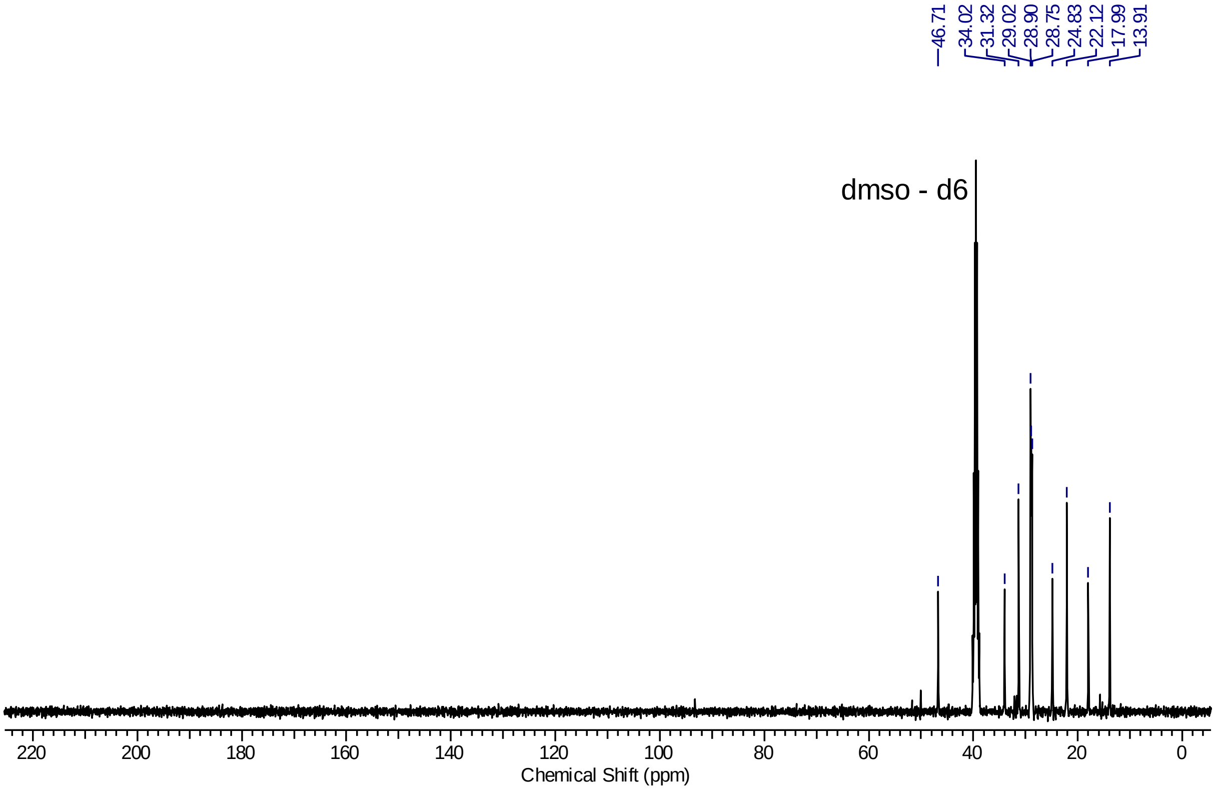

46.71
34.02
31.32
29.02
28.90
28.75
24.83
22.12
17.99
13.91
dmso - d6
220
200
180
160
140
120
100
80
60
40
20
0
Chemical Shift (ppm)

30:

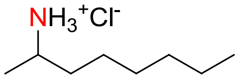
NH3+Cl-

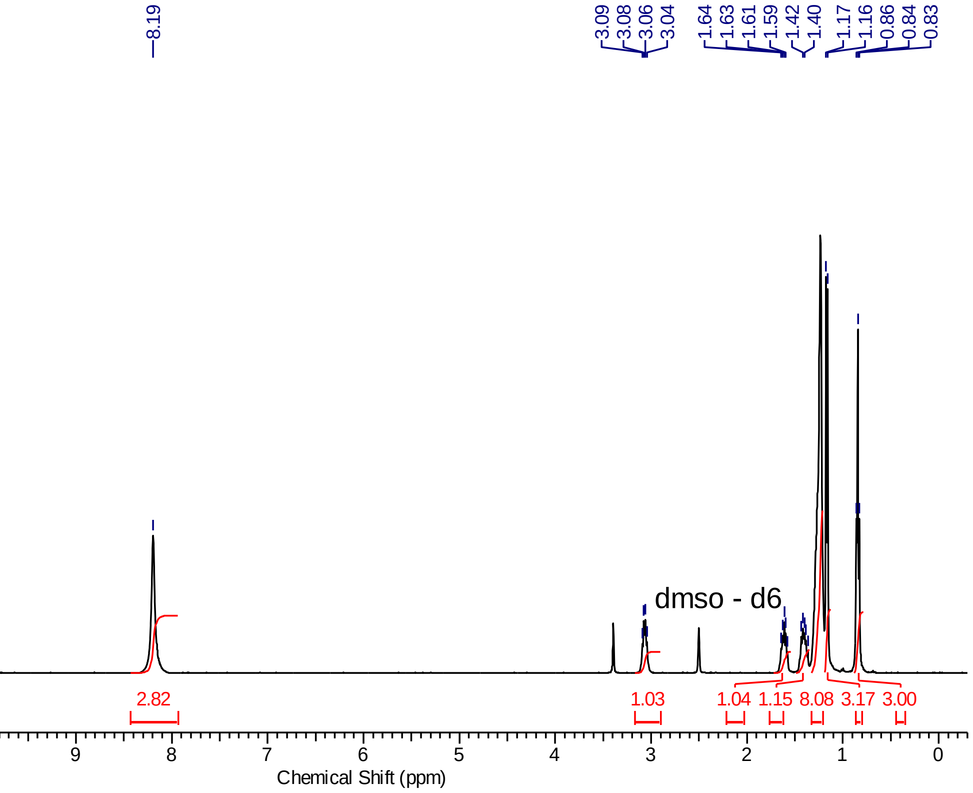
8.19
3.09
3.08
3.06
3.04
1.64
1.63
1.61
1.59
1.42
1.40
1.17
1.16
0.86
0.84
0.83
dmso - d6
2.82
1.03
1.04
1.15
8.08
3.17
3.00
12
11
10
9
8
7
6
5
4
3
2
1
0
Chemical Shift (ppm)

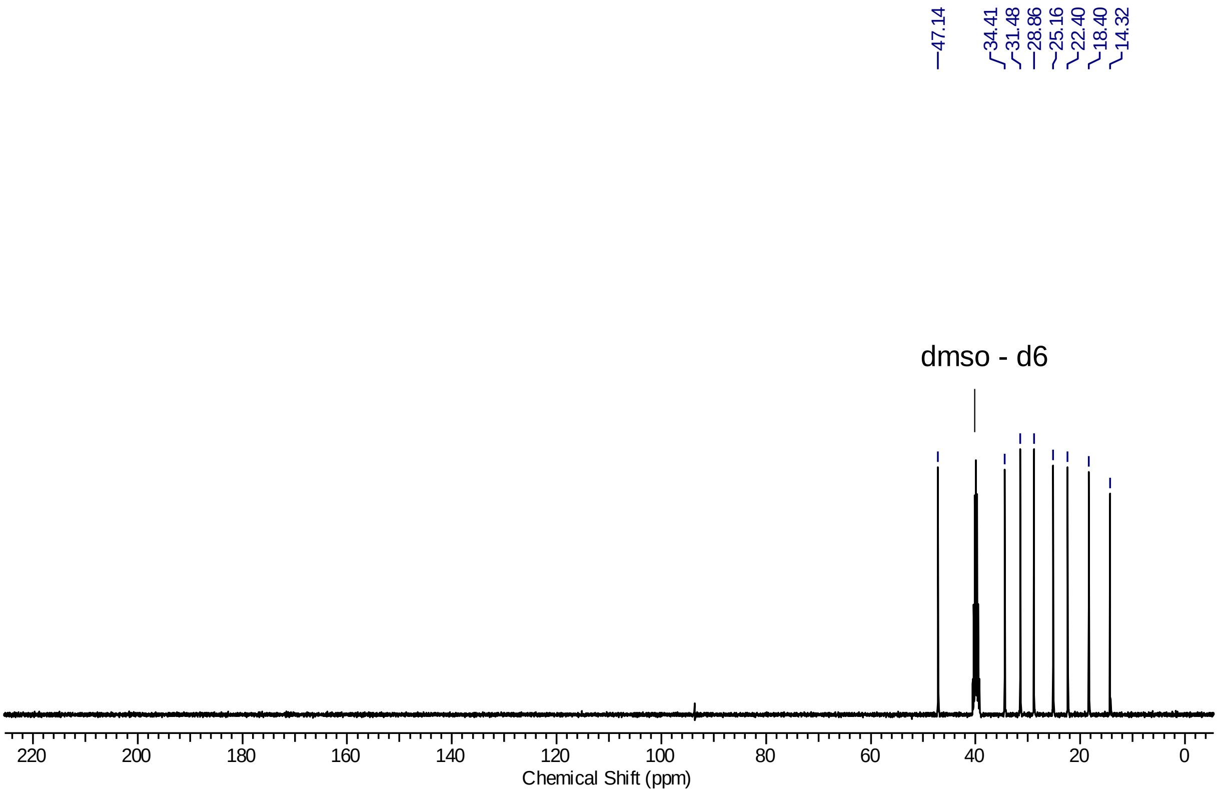
47.14
34.41
31.48
28.86
25.16
22.40
18.40
14.32
dmso - d6
220
200
180
160
140
120
100
80
60
40
20
0
Chemical Shift (ppm)

31:

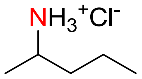
NH3+Cl-

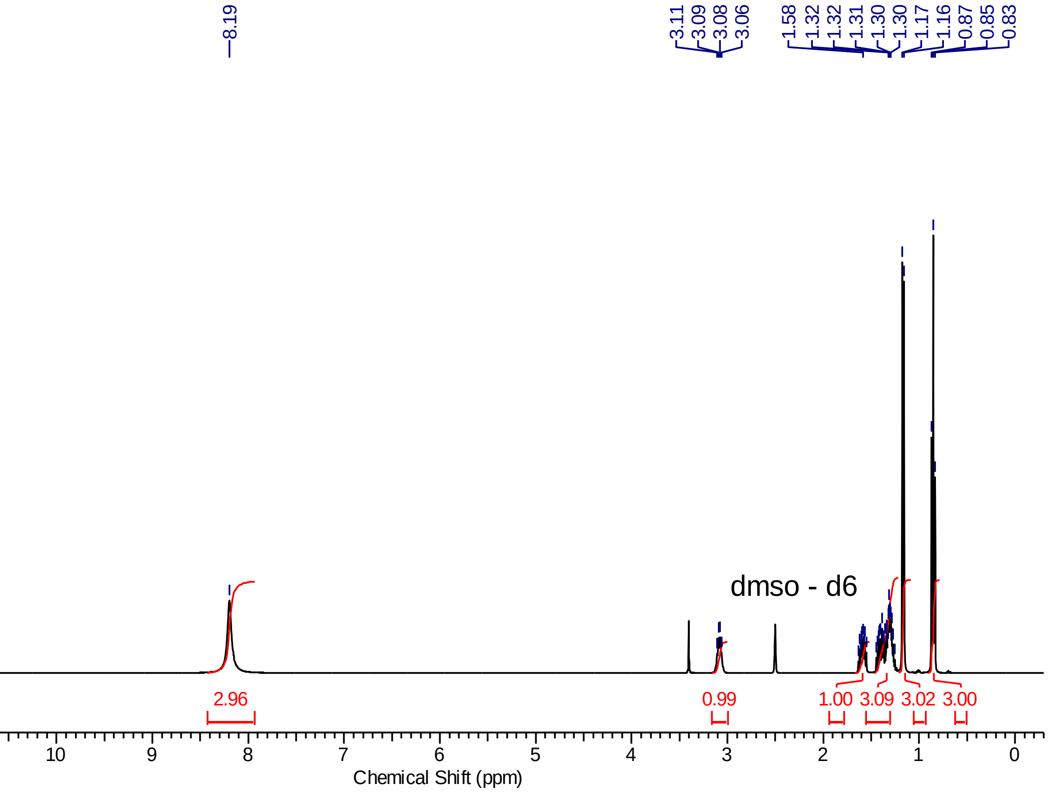
8.19
3.11
3.09
3.08
3.06
1.58
1.32
1.32
1.31
1.30
1.30
1.17
1.16
0.87
0.85
0.83
dmso - d6
2.96
0.99
1.00
3.09
3.02
3.00
12
11
10
9
8
7
6
5
4
3
2
1
0
Chemical Shift (ppm)

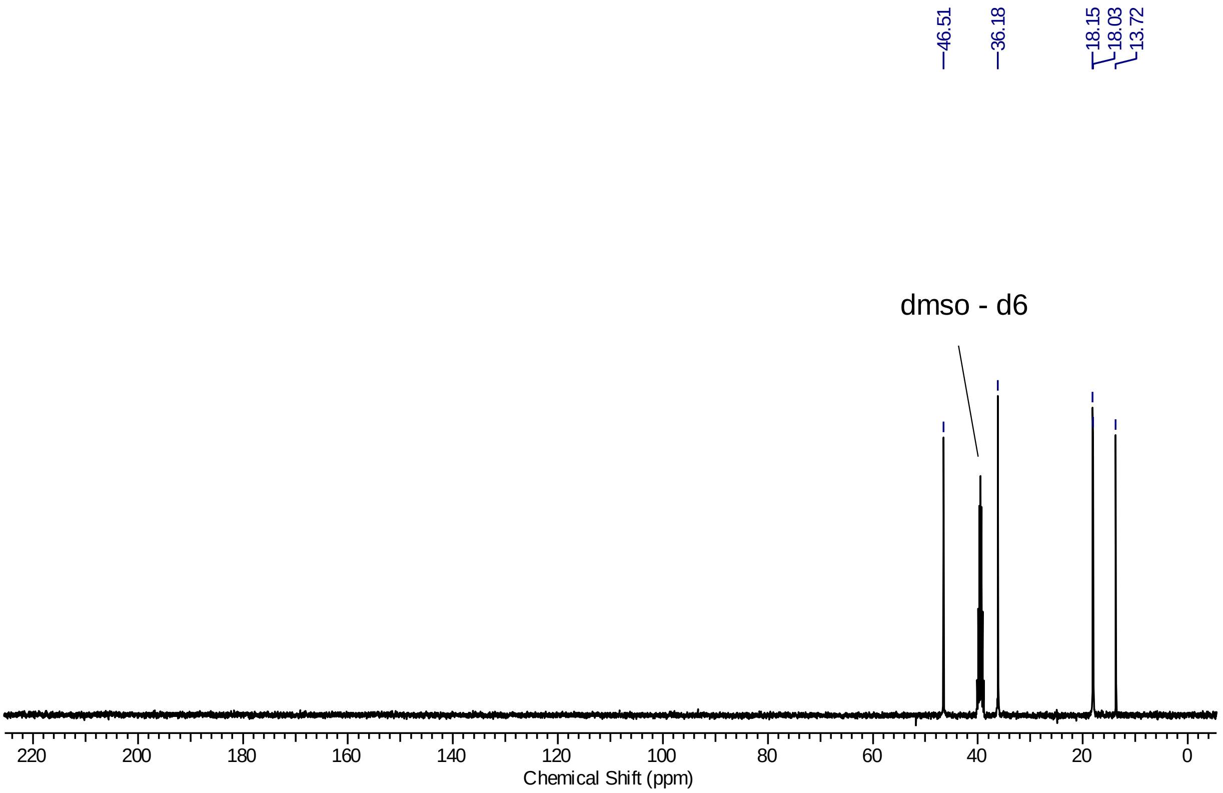
46.51
36.18
18.15
18.03
13.72
dmso - d6
220
200
180
160
140
120
100
80
60
40
20
0
Chemical Shift (ppm)

32:

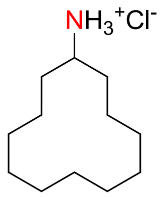
NH$_3^+$Cl$^-$

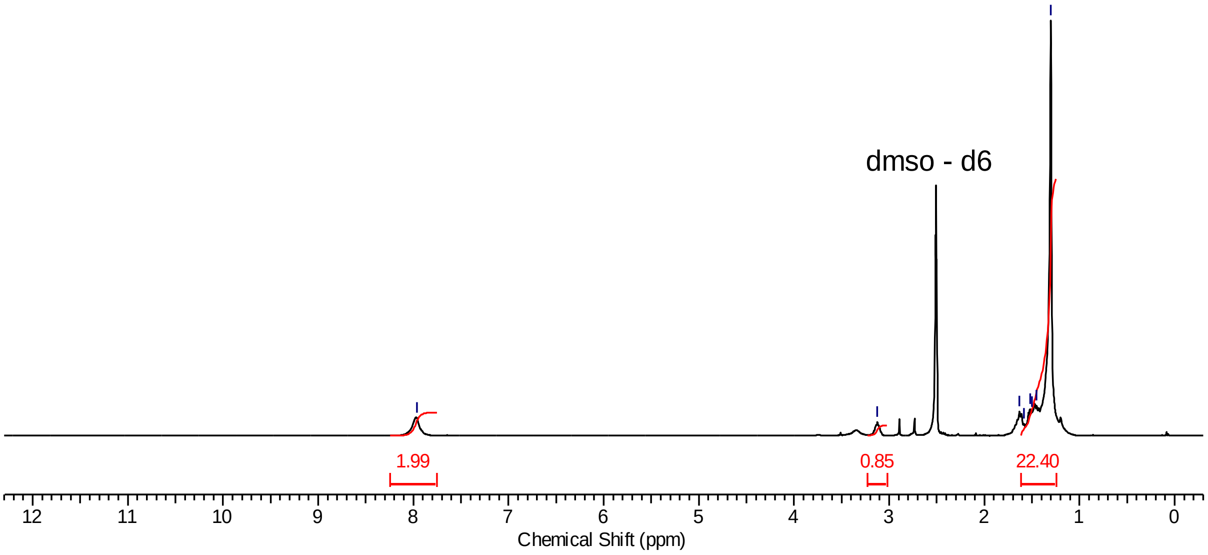
2386.73
936.49
489.01
474.36
455.62
449.18
435.12
390.60
dmso - d6
1.99
0.85
22.40
12 11 10 9 8 7 6 5 4 3 2 1 0
Chemical Shift (ppm)

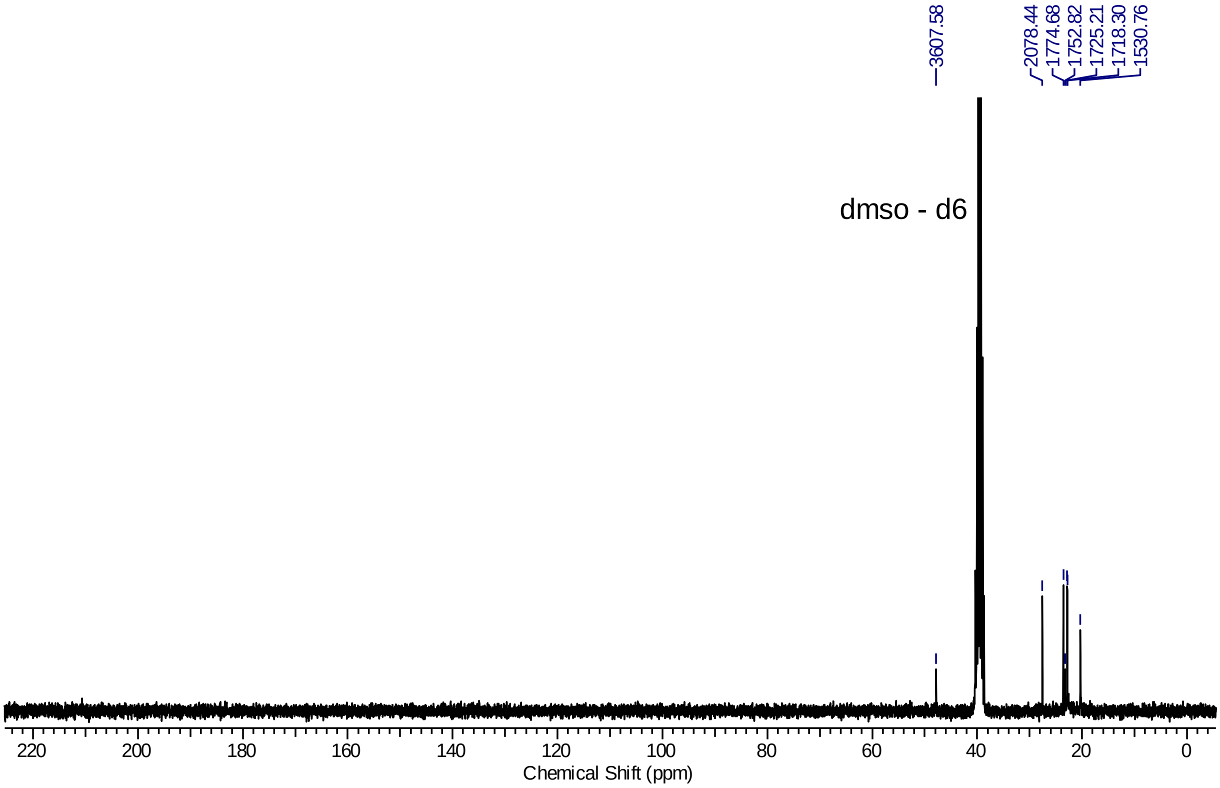
3607.58
2078.44
1774.68
1752.82
1725.21
1718.30
1530.76
dmso - d6
220 200 180 160 140 120 100 80 60 40 20 0
Chemical Shift (ppm)

33:

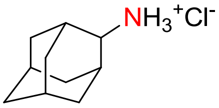
NH3+Cl-

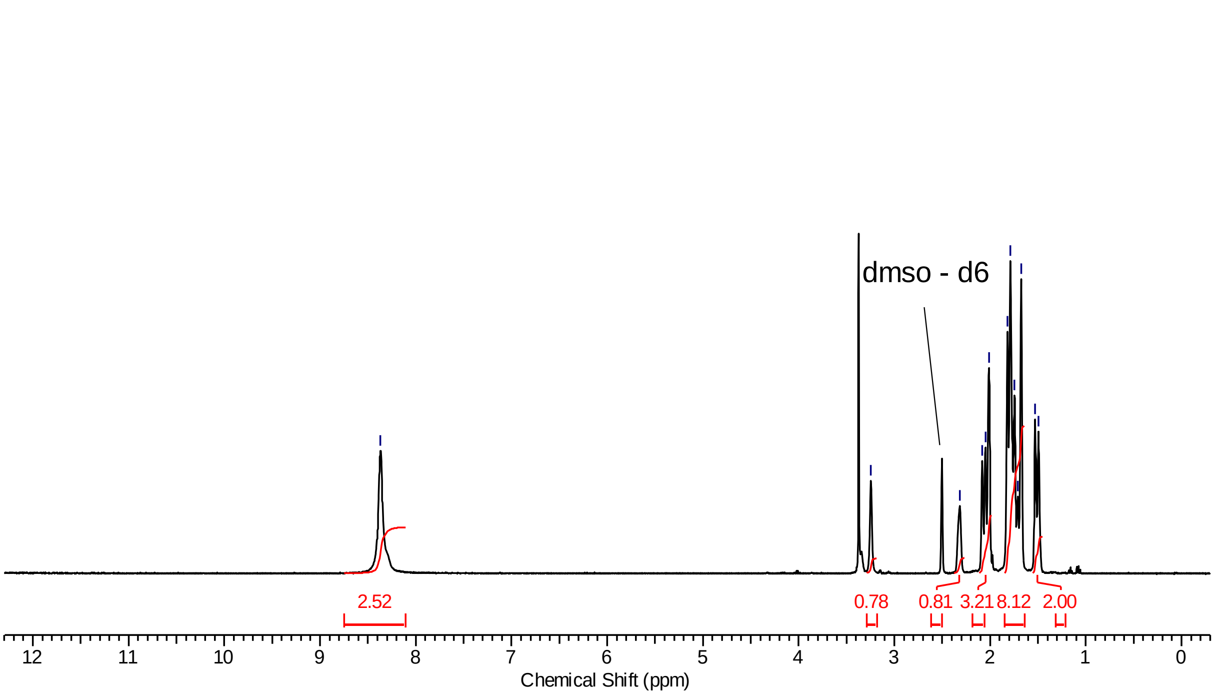
8.37
3.24
2.32
2.08
2.05
2.01
1.82
1.79
1.74
1.71
1.68
1.53
1.50
dmso - d6
2.52
0.78
0.81
3.21
8.12
2.00
12 11 10 9 8 7 6 5 4 3 2 1 0
Chemical Shift (ppm)

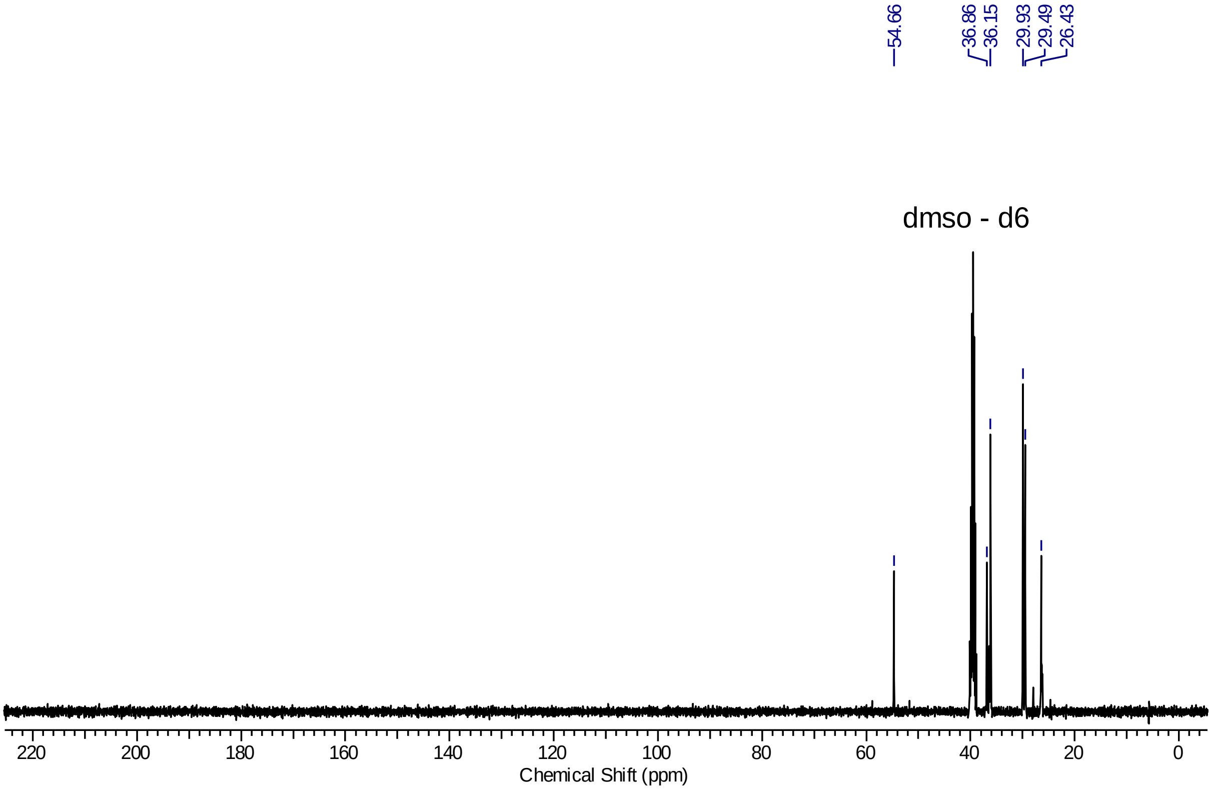
54.66
36.86
36.15
29.93
29.49
26.43
dmso - d6
220 200 180 160 140 120 100 80 60 40 20 0
Chemical Shift (ppm)

34:

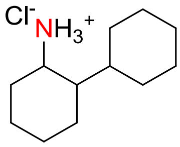
Cl⁻NH₃⁺

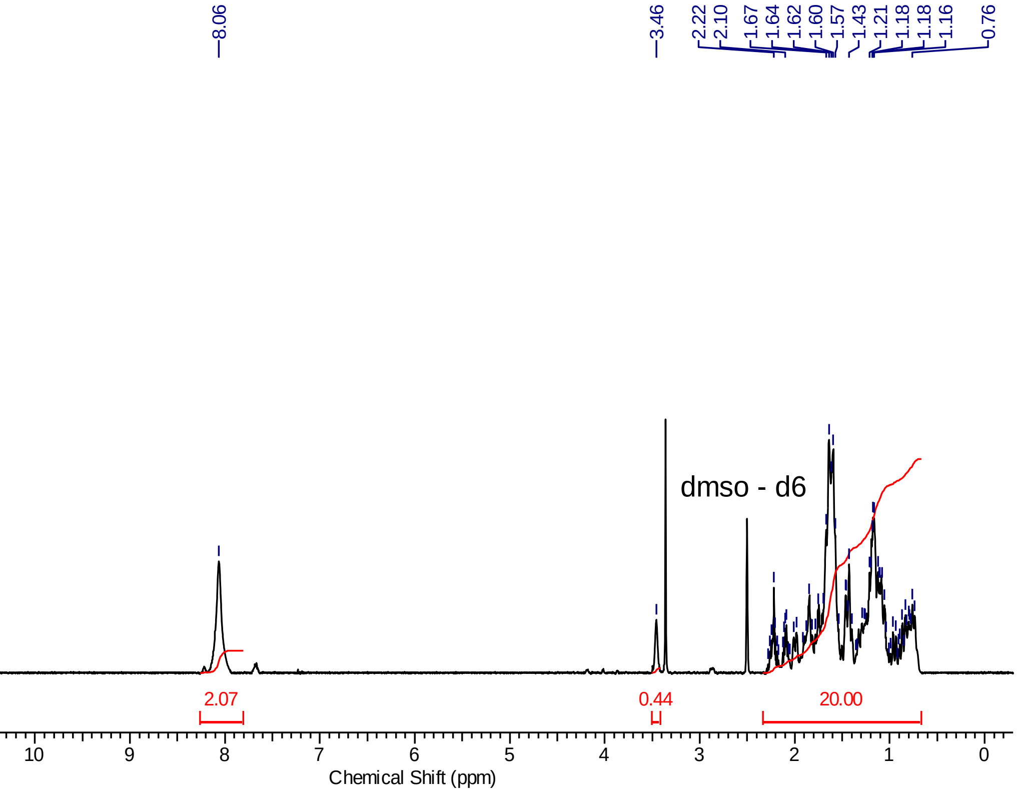
8.06
3.46
2.22 2.10 1.67 1.64 1.62 1.60 1.57 1.43 1.21 1.18 1.18 1.16 0.76
dmso - d6
2.07
0.44
20.00
Chemical Shift (ppm)

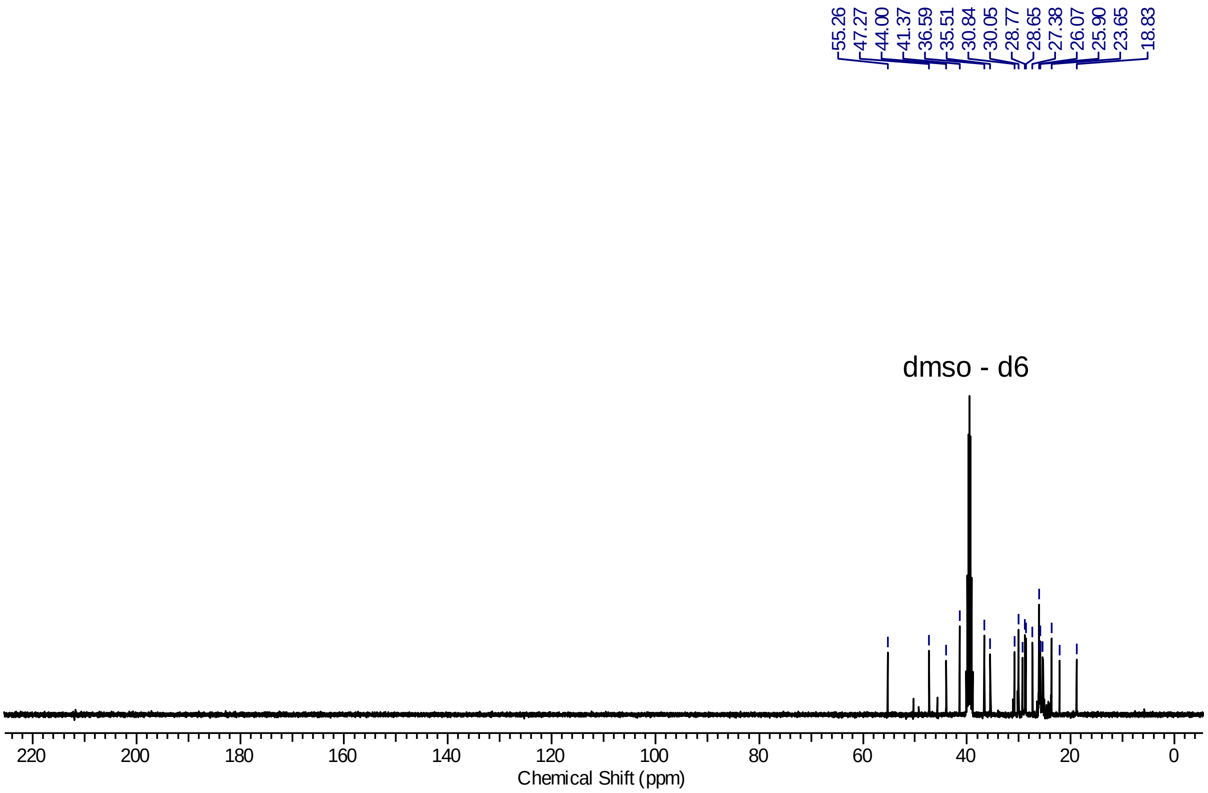
55.26 47.27 44.00 41.37 36.59 35.51 30.84 30.05 28.77 28.65 27.38 26.07 25.90 23.65 18.83
dmso - d6
Chemical Shift (ppm)

35:

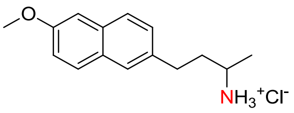
O
NH3+Cl-

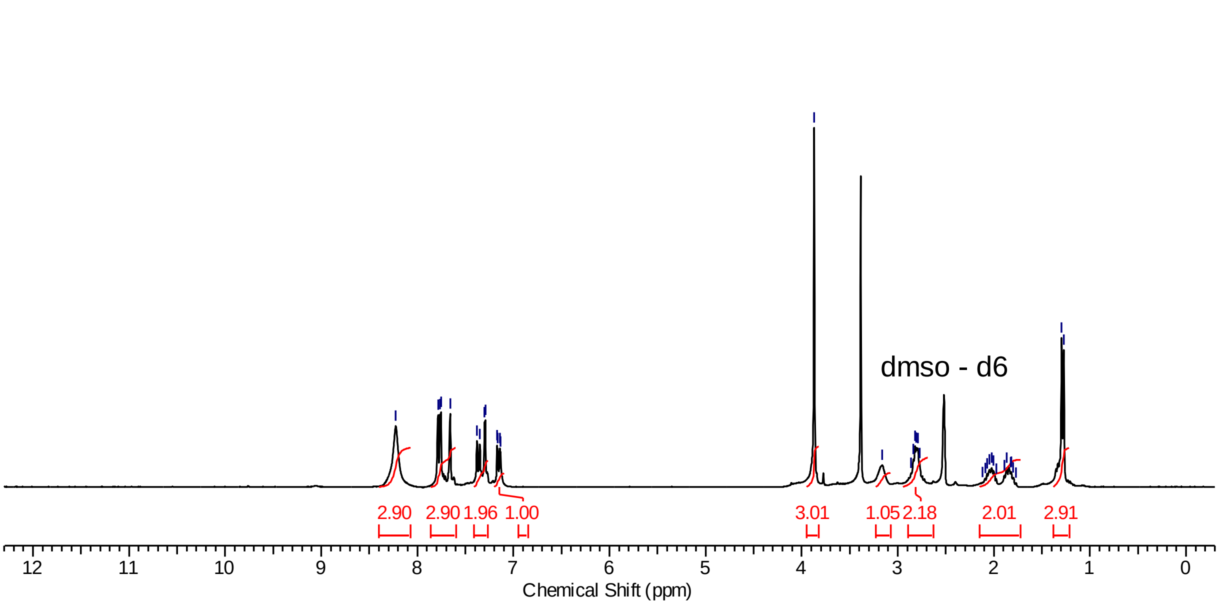
8.22
7.78
7.76
7.75
7.66
7.38
7.35
7.30
7.29
7.17
7.14
3.87
3.16
2.86
2.84
2.82
2.81
2.79
2.77
2.05
2.02
2.00
1.86
1.82
1.82
1.29
1.27
dmso - d6
2.90
2.90
1.96
1.00
3.01
1.05
2.18
2.01
2.91
12
11
10
9
8
7
6
5
4
3
2
1
0
Chemical Shift (ppm)

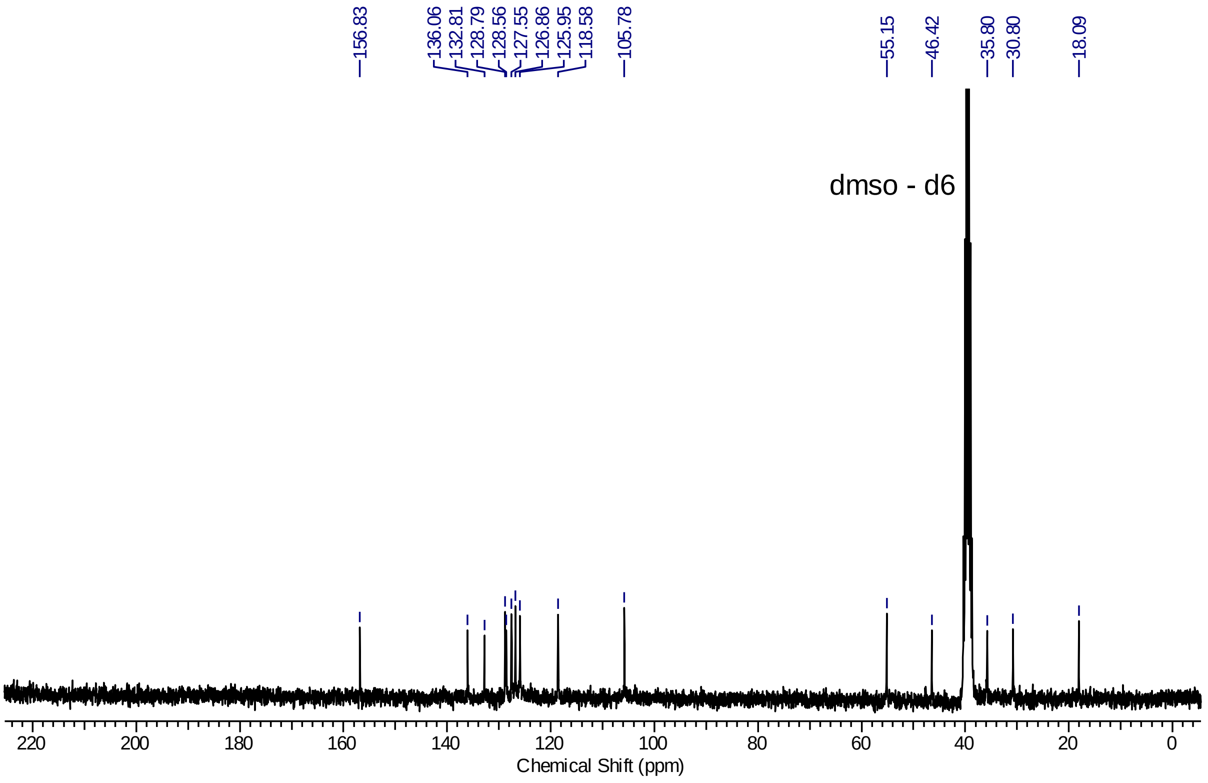
156.83
136.06
132.81
128.79
128.56
127.55
126.86
125.95
118.58
105.78
55.15
46.42
35.80
30.80
18.09
dmso - d6
220
200
180
160
140
120
100
80
60
40
20
0
Chemical Shift (ppm)

36:

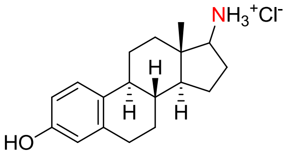
NH3+Cl-
H
H
H
HO

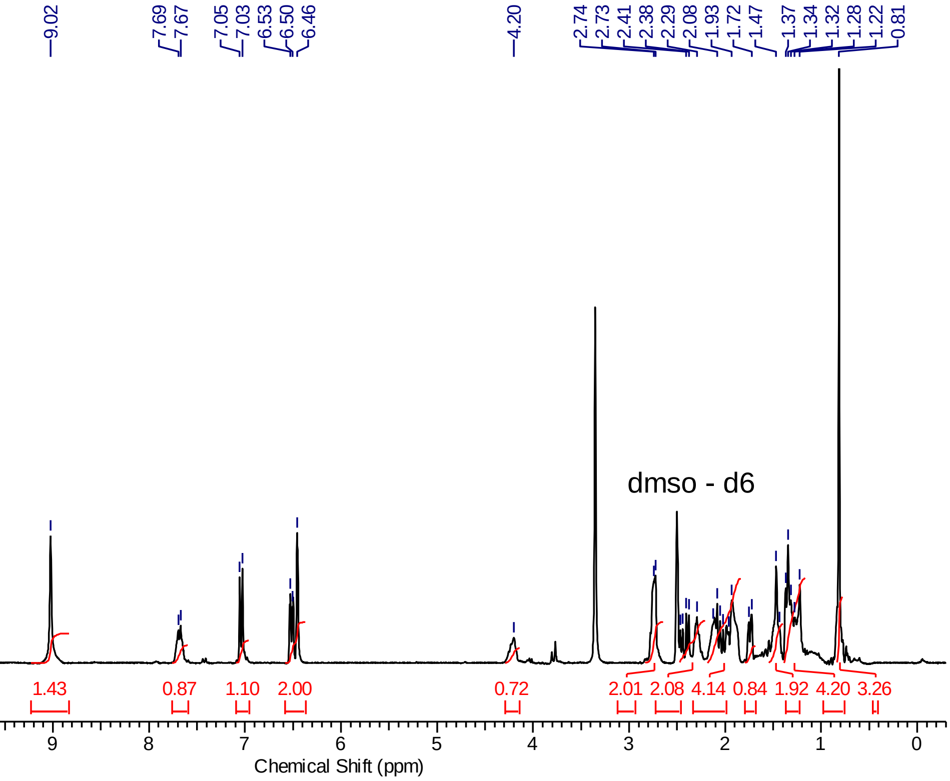
9.02
7.69
7.67
7.05
7.03
6.53
6.50
6.46
4.20
2.74
2.73
2.41
2.38
2.29
2.08
1.93
1.72
1.47
1.37
1.34
1.32
1.28
1.22
0.81
dmso - d6
1.43
0.87
1.10
2.00
0.72
2.01
2.08
4.14
0.84
1.92
4.20
3.26
12
11
10
9
8
7
6
5
4
3
2
1
0
Chemical Shift (ppm)

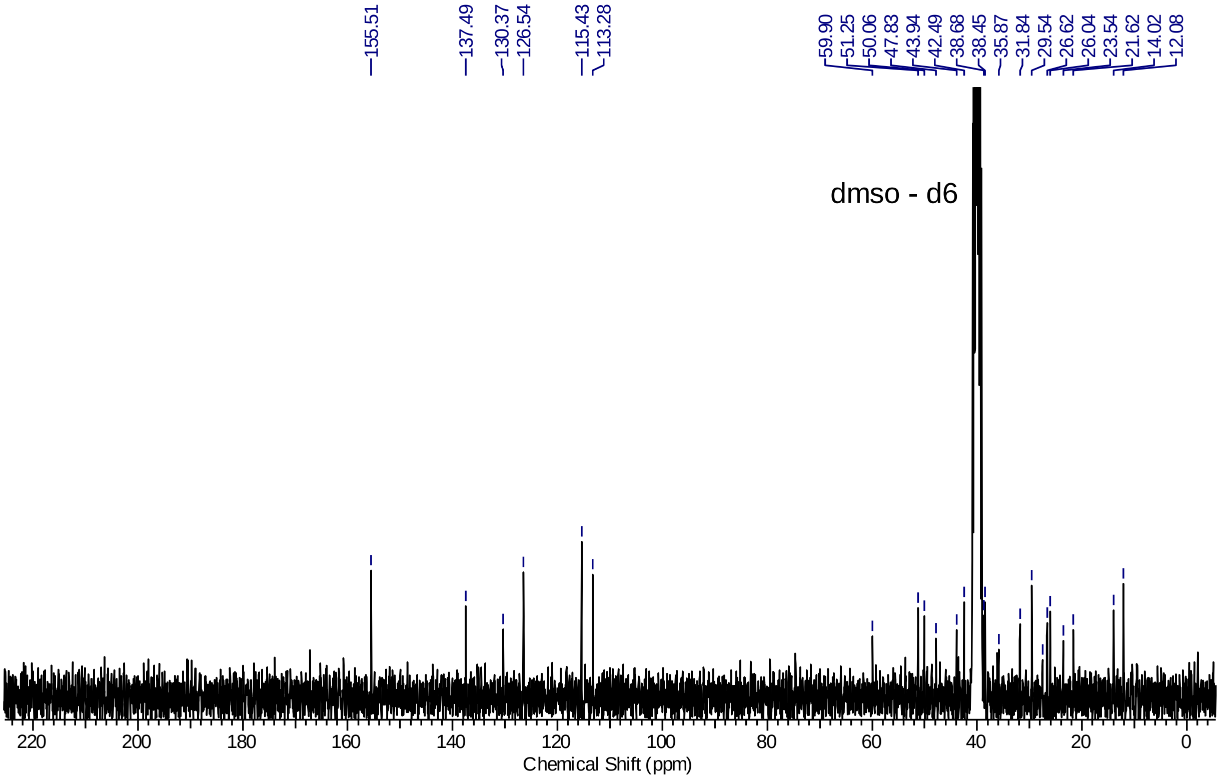
155.51
137.49
130.37
126.54
115.43
113.28
59.90
51.25
50.06
47.83
43.94
42.49
38.68
38.45
35.87
31.84
29.54
26.62
26.04
23.54
21.62
14.02
12.08
dmso - d6
220
200
180
160
140
120
100
80
60
40
20
0
Chemical Shift (ppm)

37:

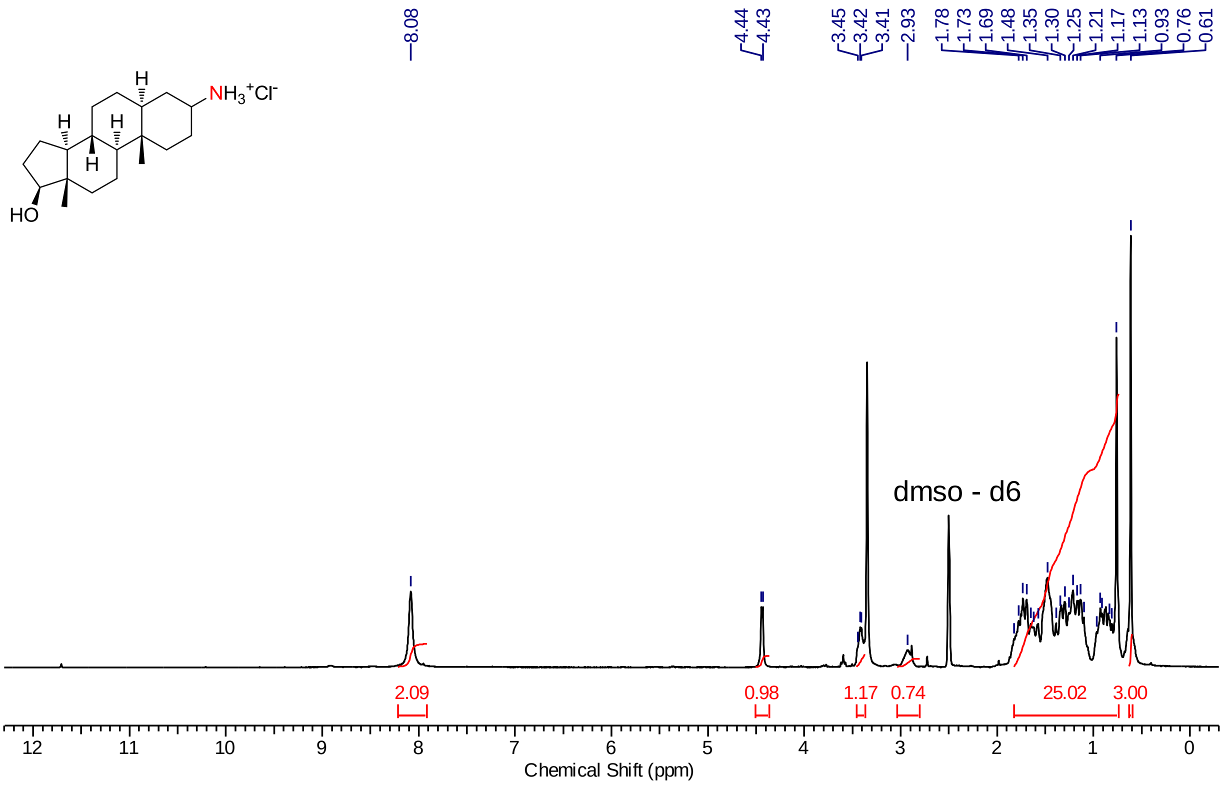

NH3+Cl-
HO
H
8.08
4.44
4.43
3.45
3.42
3.41
2.93
1.78
1.73
1.69
1.48
1.35
1.30
1.25
1.21
1.17
1.13
0.93
0.76
0.61
dmso - d6
2.09
0.98
1.17
0.74
25.02
3.00
Chemical Shift (ppm)

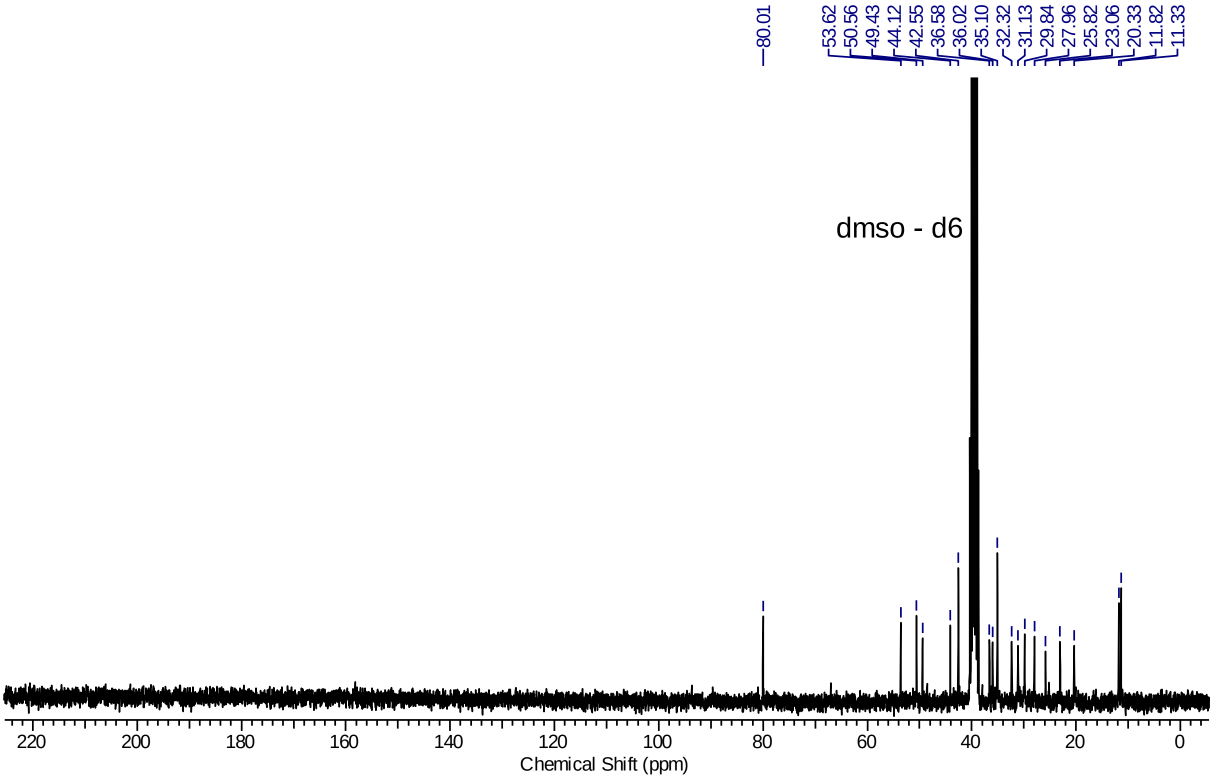

80.01
53.62
50.56
49.43
44.12
42.55
36.58
36.02
35.10
32.32
31.13
29.84
27.96
25.82
23.06
20.33
11.82
11.33
dmso - d6
Chemical Shift (ppm)

38:

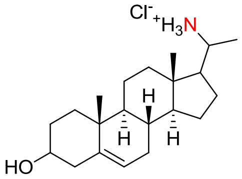
Cl⁻ ⁺H3N
H
H
H
HO

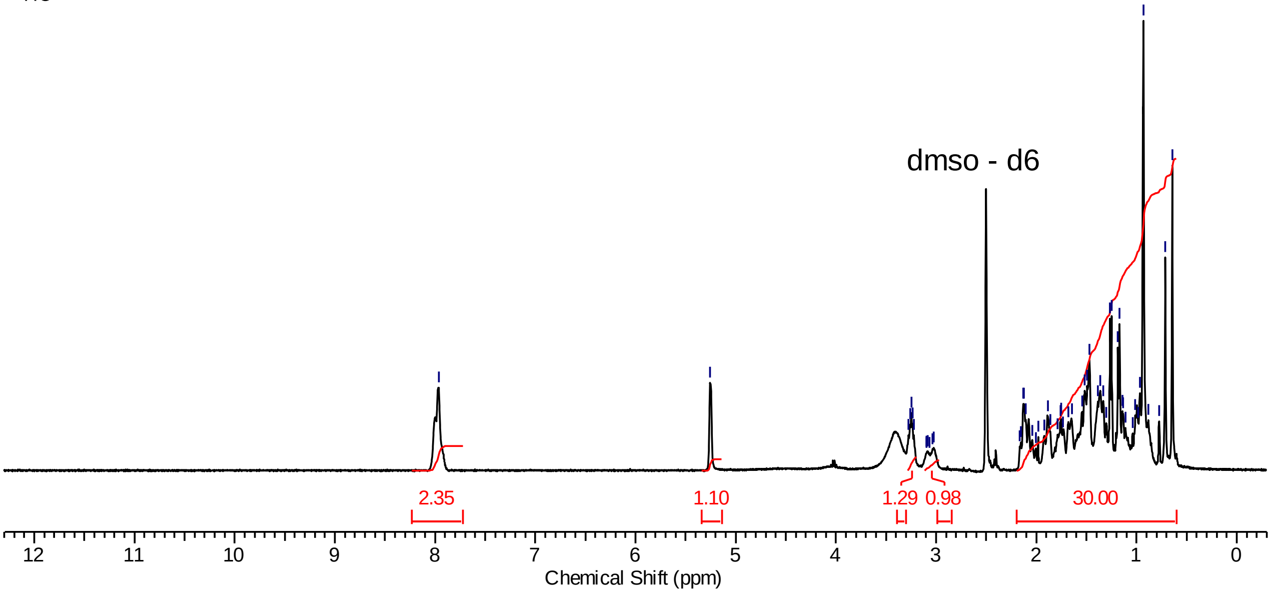
7.96
5.26
3.27
3.26
3.25
3.24
3.04
2.13
2.12
1.88
1.52
1.49
1.47
1.36
1.27
1.25
1.19
1.17
0.93
0.71
0.64
dmso - d6
2.35
1.10
1.29
0.98
30.00
12
11
10
9
8
7
6
5
4
3
2
1
0
Chemical Shift (ppm)

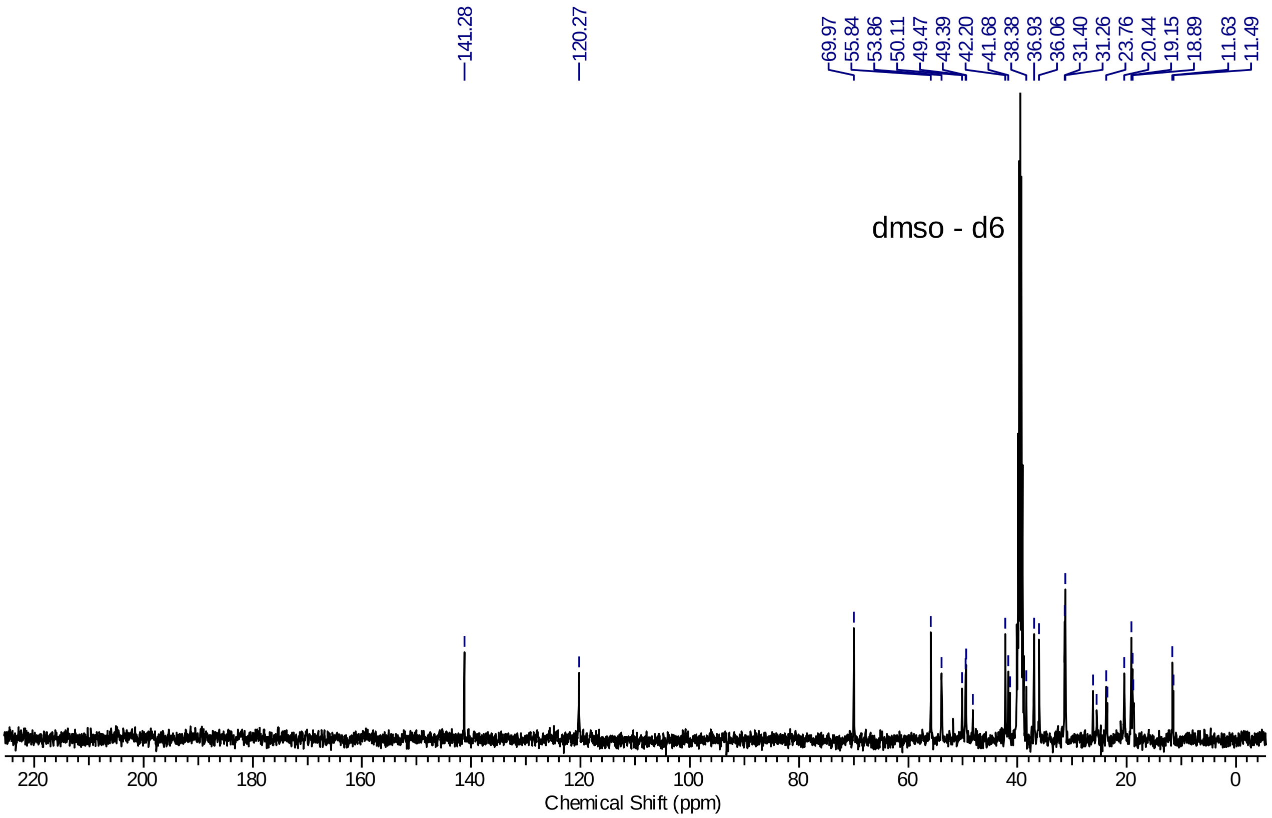
141.28
120.27
69.97
55.84
53.86
50.11
49.47
49.39
42.20
41.68
38.38
36.93
36.06
31.40
31.26
23.76
20.44
19.15
18.89
11.63
11.49
dmso - d6
220
200
180
160
140
120
100
80
60
40
20
0
Chemical Shift (ppm)

39:

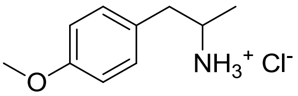
O
NH3+ Cl-

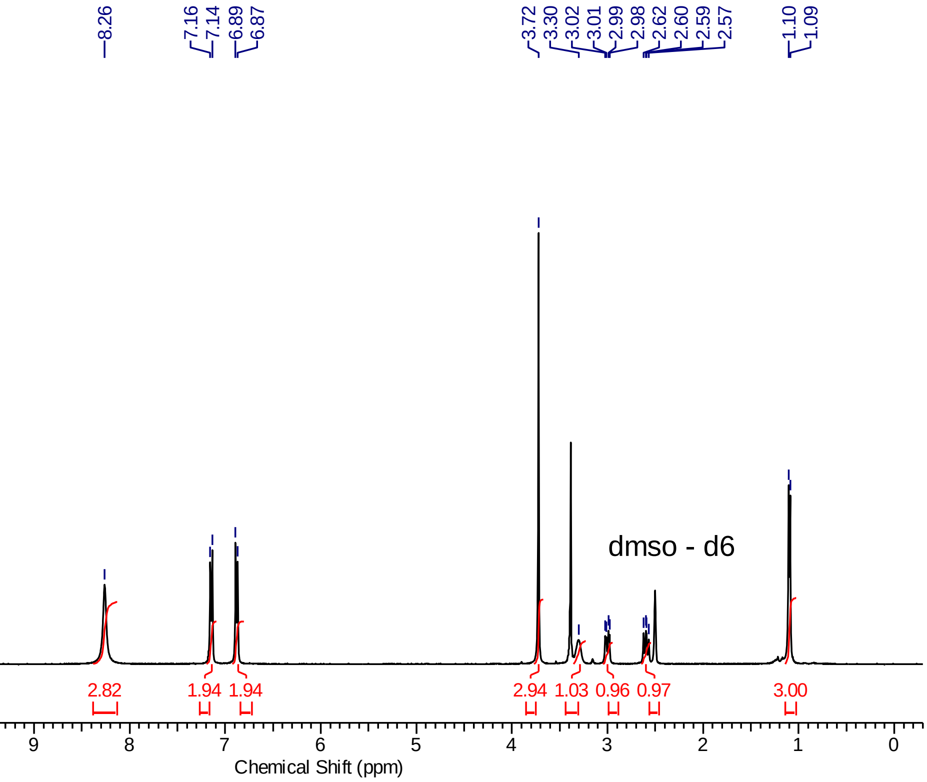
8.26
7.16
7.14
6.89
6.87
3.72
3.30
3.02
3.01
2.99
2.98
2.62
2.60
2.59
2.57
1.10
1.09
dmso - d6
2.82
1.94
1.94
2.94
1.03
0.96
0.97
3.00
12
11
10
9
8
7
6
5
4
3
2
1
0
Chemical Shift (ppm)

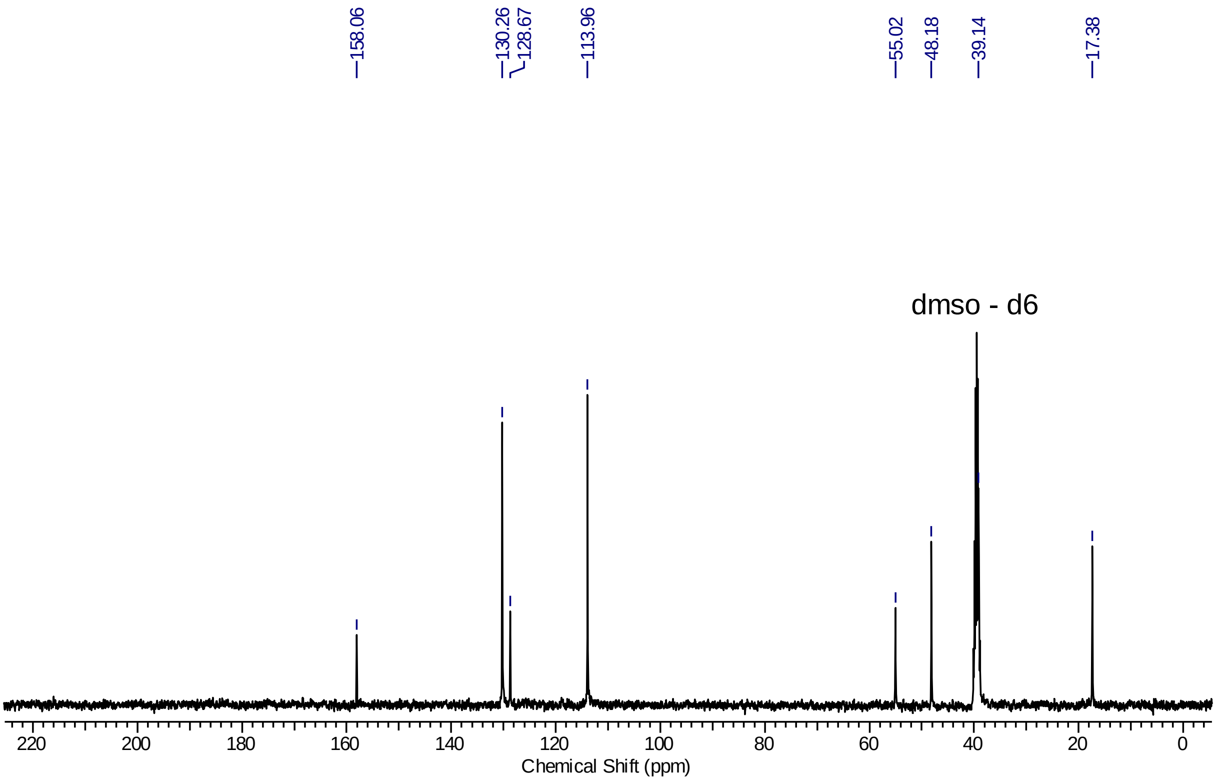
158.06
130.26
128.67
113.96
55.02
48.18
39.14
17.38
dmso - d6
220
200
180
160
140
120
100
80
60
40
20
0
Chemical Shift (ppm)

40:

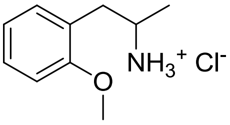

NH3+ Cl-
O

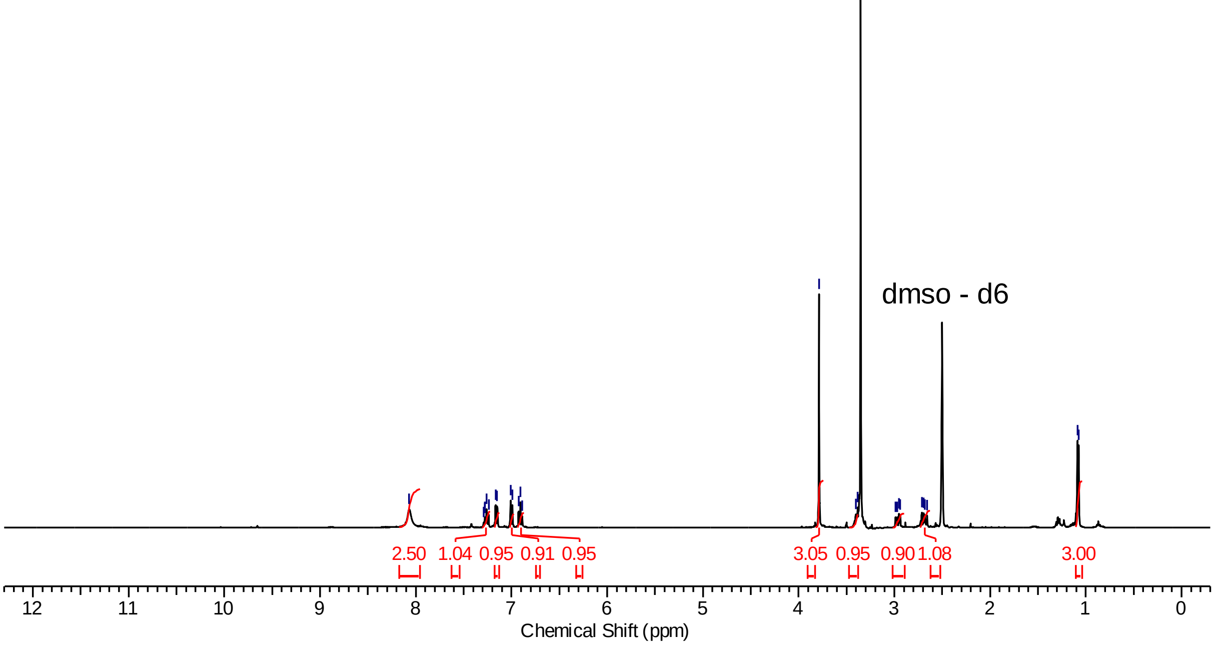

8.07
7.26
7.24
7.16
7.15
7.01
6.99
6.92
6.91
6.89
3.79
3.40
3.39
2.97
2.95
2.94
2.71
2.69
2.68
2.66
1.09
1.07
dmso - d6
2.50
1.04
0.95
0.91
0.95
3.05
0.95
0.90
1.08
3.00
12
11
10
9
8
7
6
5
4
3
2
1
0
Chemical Shift (ppm)

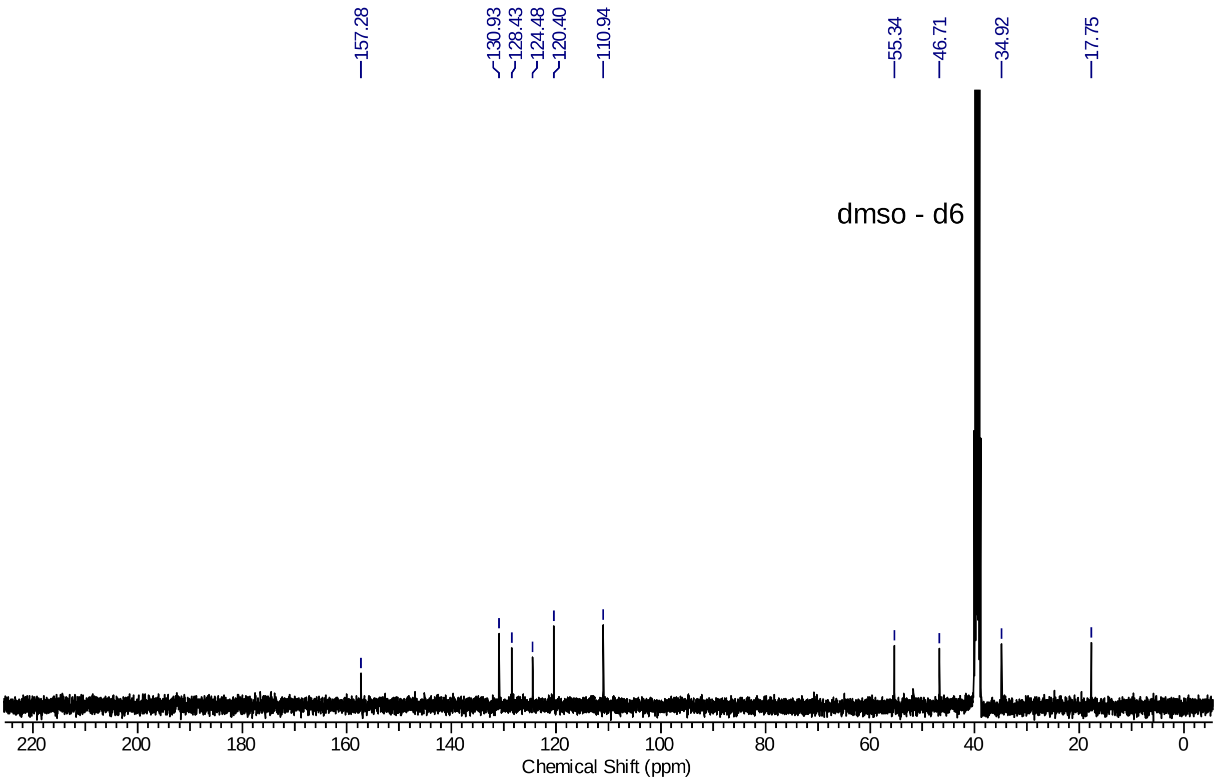

157.28
130.93
128.43
124.48
120.40
110.94
55.34
46.71
34.92
17.75
dmso - d6
220
200
180
160
140
120
100
80
60
40
20
0
Chemical Shift (ppm)

2.9 FTIR spectroscopy

Ligand I:

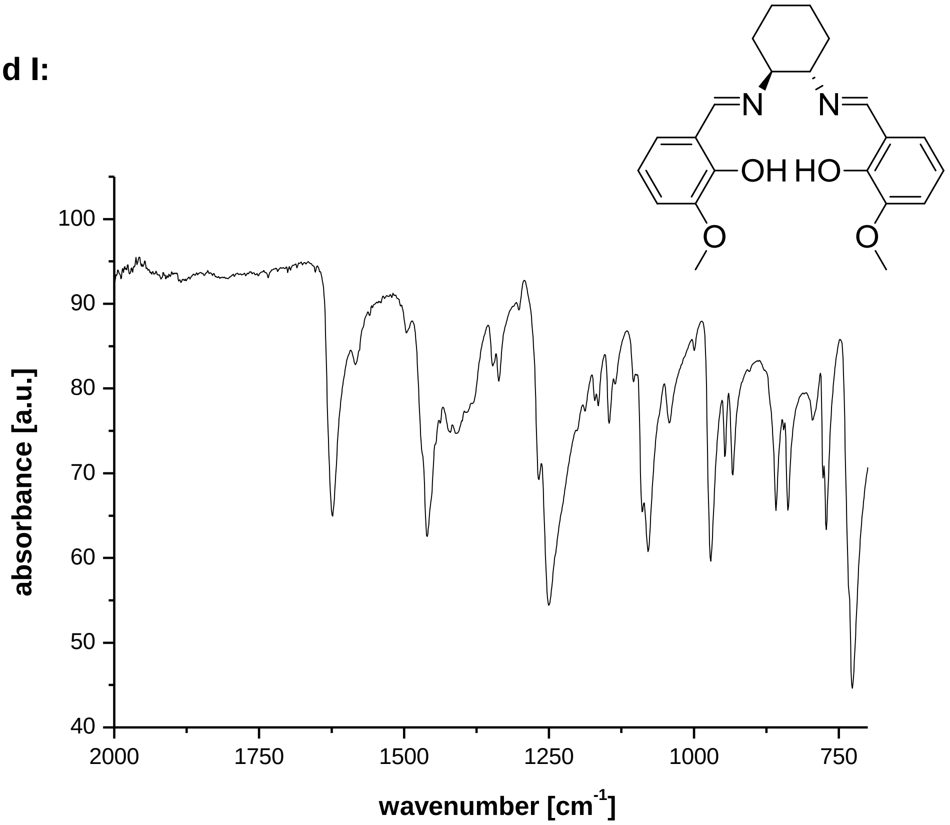

Complex I:

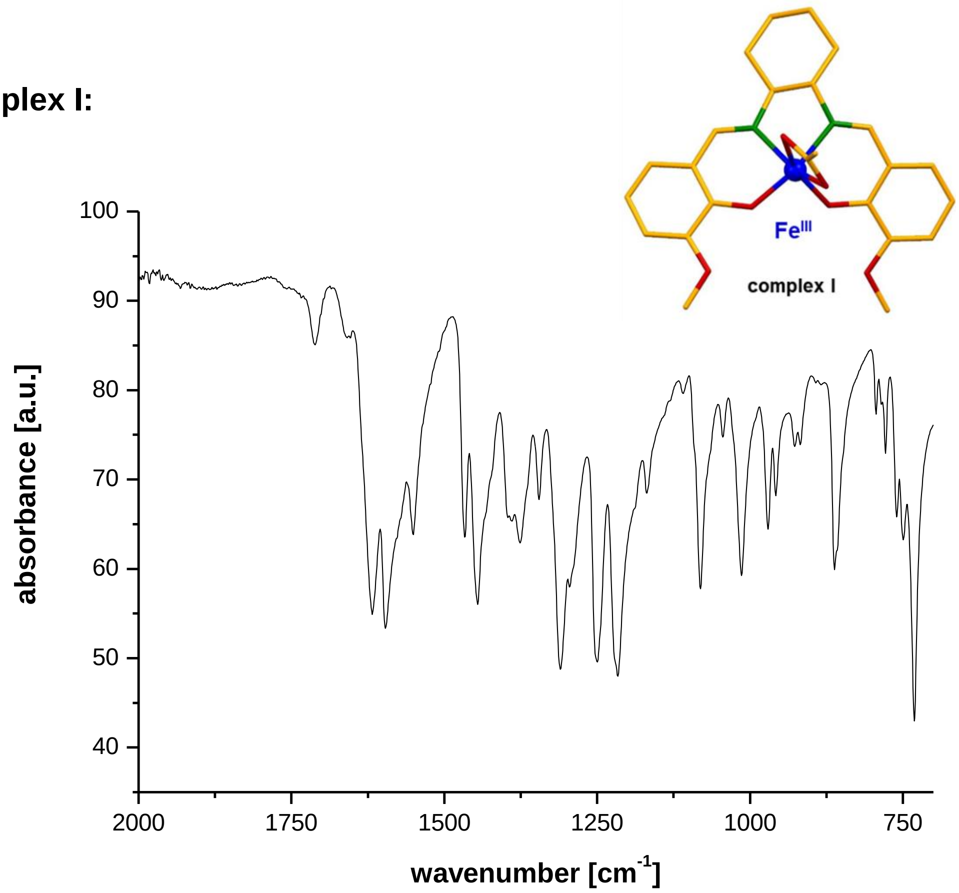

Complex II:

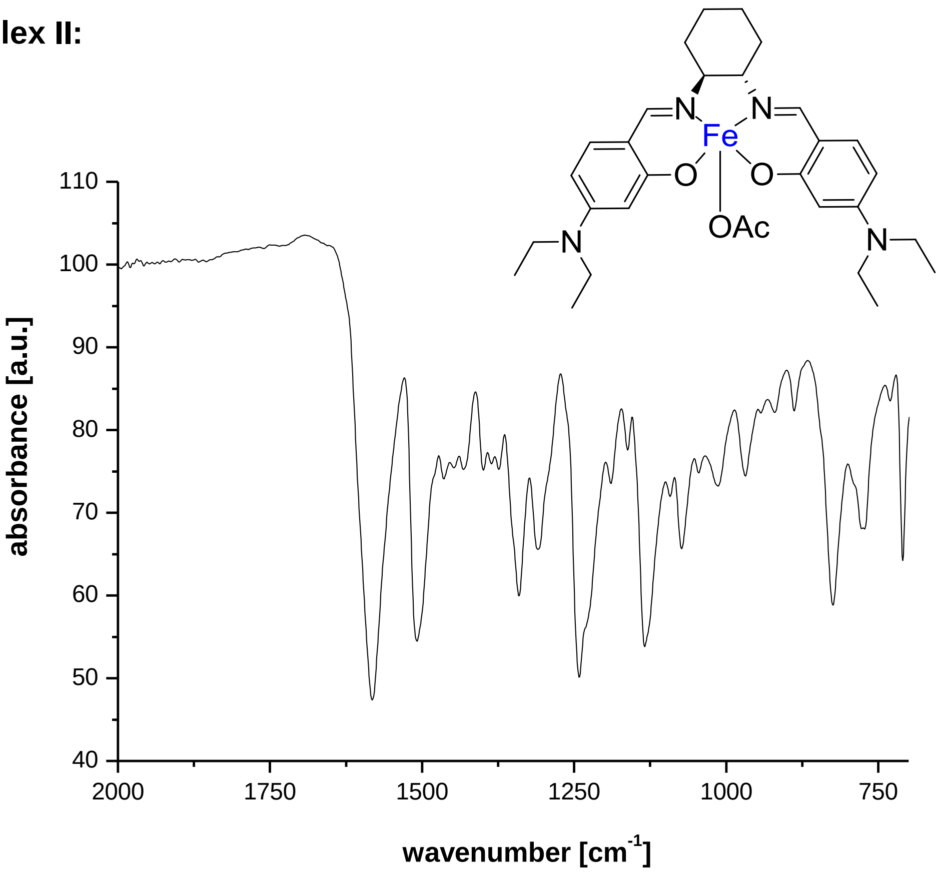

Complex III:

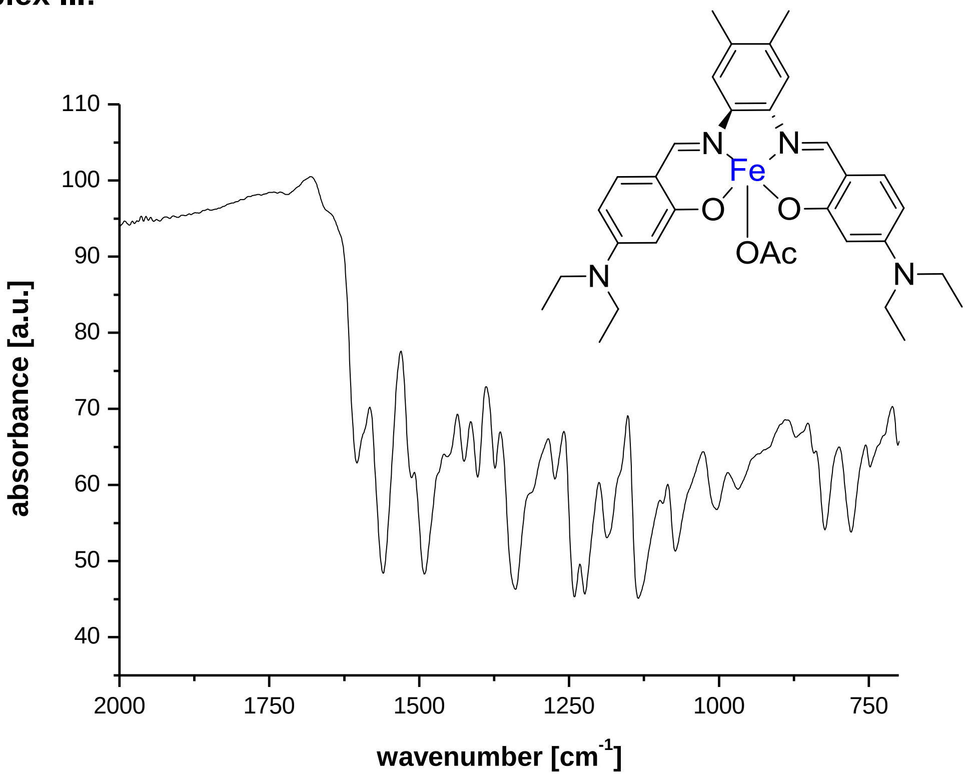

Complex IV:

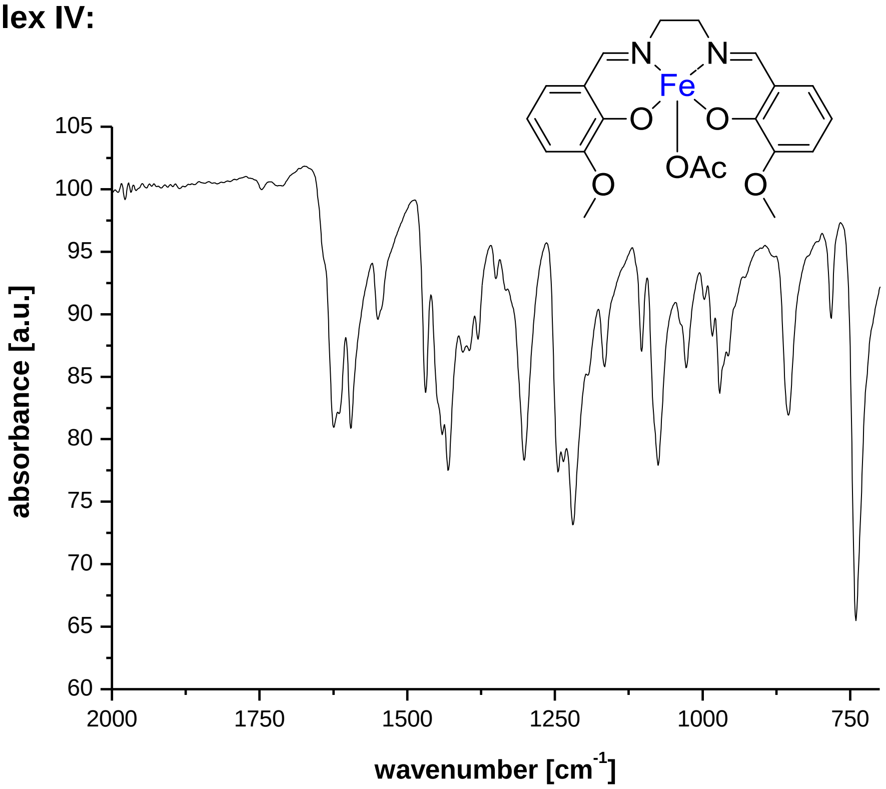

Complex V:

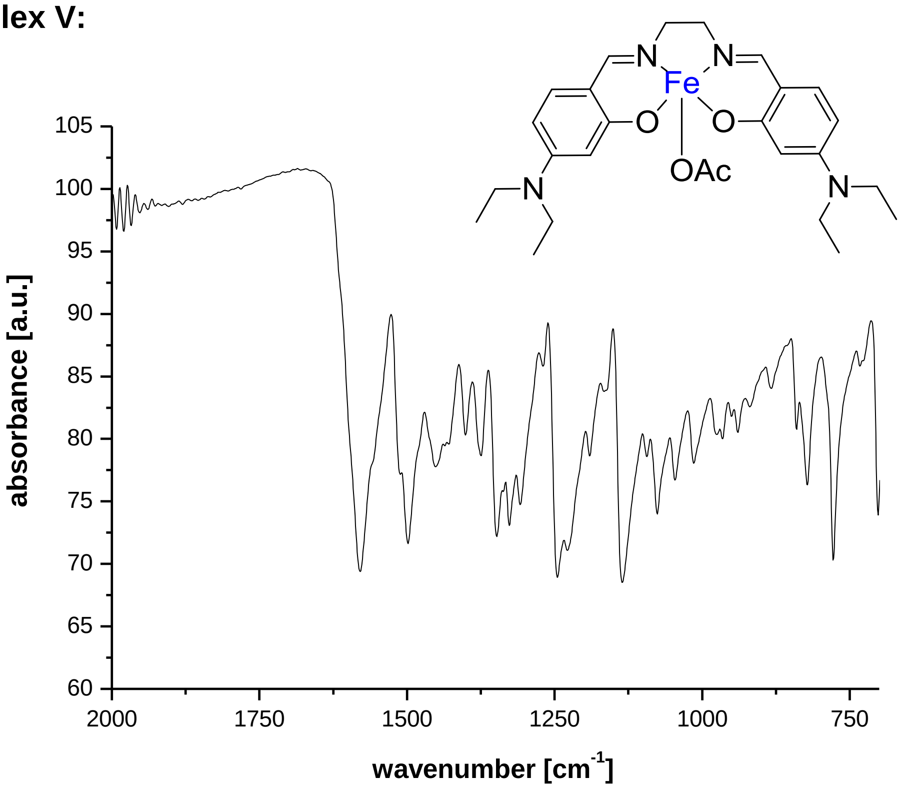

Complex VI:

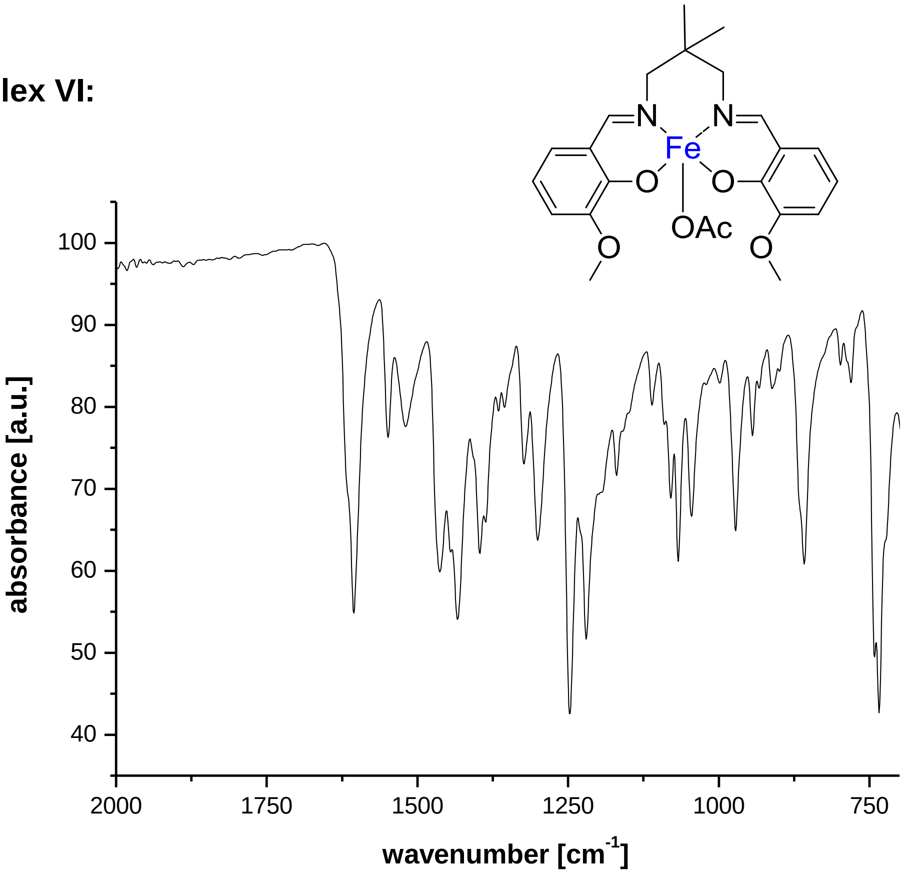
N
N
Fe
O
O
OAc
O
O
100
90
80
70
60
50
40
absorbance [a.u.]
2000
1750
1500
1250
1000
750
wavenumber [cm-1]

2.10 Crystallographic data of complex I

The Fe complex I was dissolved in acetonitrile and crystalized by -32 °C (purple needles).
Deposition Number: 1895788

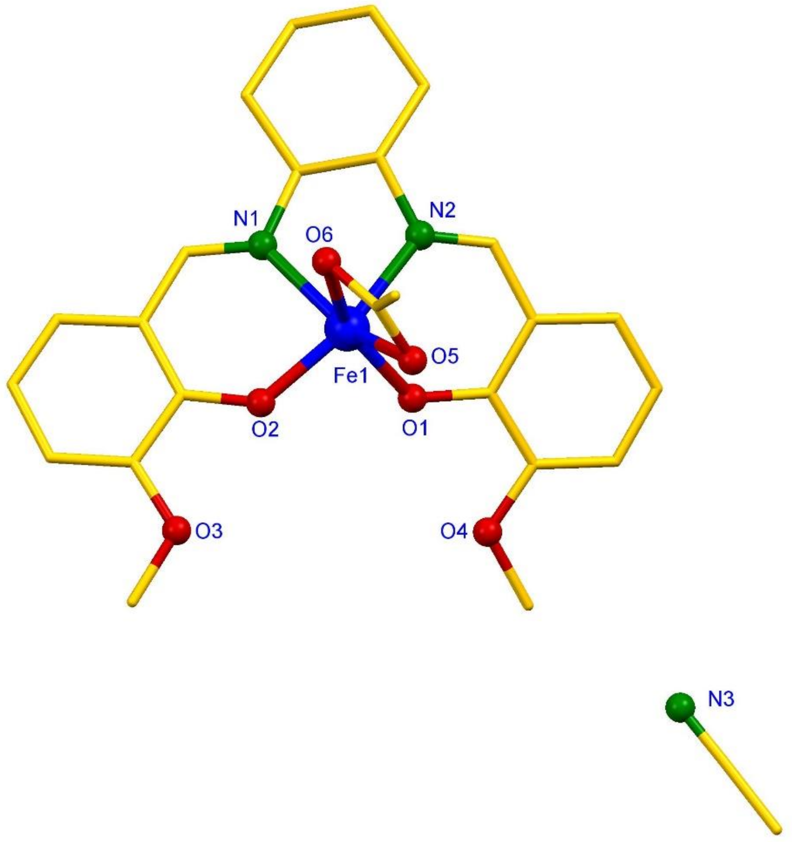

checkCIF/PLATON (basic structural check)

Structure factors have been supplied for datablock(s) sv_452_p21n

THIS REPORT IS FOR GUIDANCE ONLY. IF USED AS PART OF A REVIEW PROCEDURE FOR PUBLICATION, IT SHOULD NOT REPLACE THE EXPERTISE OF AN EXPERIENCED CRYSTALLOGRAPHIC REFEREE.

No syntax errors found. CIF dictionary Interpreting this report

Structure factor report

Datablock: sv_452_p21n

Bond precision:	C-C = 0.0020 A		Wavelength=0.71073
Cell:	a=12.048(2)	b=12.534(3)	c=16.704(3)
	alpha=90	beta=98.75(3)	gamma=90
Temperature:	133 K		

	Calculated	Reported
Volume	2493.1(9)	2493.1(9)
Space group	P 21/n	P 1 21/n 1
Hall group	-P 2yn	-P 2ybc (x-
Moiety formula	C24 H27 Fe N2 O6, C2 H3 N	C24 H27 Fe N2 O6, C2 H3 N
Sum formula	C26 H30 Fe N3 O6	C26 H30 Fe N3 O6
Mr	536.38	536.39
Dx,g cm-3	1.429	1.429
Z	4	4
Mu (mm-1)	0.652	0.652
F000	1124.0	1125.9
F000'	1125.86	
h,k,lmax	16,16,22	16,16,22
Nref	6324	6084
Tmin,Tmax	0.978,0.991	0.981,0.995
Tmin'	0.945	

Correction method= # Reported T Limits: Tmin=0.981 Tmax=0.995

AbsCorr = NUMERICAL

Data completeness= 0.962 Theta(max)= 28.510

R(reflections)= 0.0272(5097) wR2(reflections)= 0.0943(6084)

S = 0.746 Npar= 329

The following ALERTS were generated. Each ALERT has the format

test-name_ALERT_alert-type_alert-level.

Click on the hyperlinks for more details of the test.

Alert level C

GOODF01_ALERT_2_C The least squares goodness of fit parameter lies
outside the range 0.80 <> 2.00
Goodness of fit given = 0.746

PLAT029_ALERT_3_C _diffrn_measured_fraction_theta_full value Low . 0.962 Why?

PLAT126_ALERT_1_C Error in or Uninterpretable Hall Symbol .... -P 2YBC (X-Z,Y

PLAT911_ALERT_3_C Missing FCF Refl Between Thmin & STh/L= 0.600 31 Report

---

Alert level G

PLAT066_ALERT_1_G Predicted and Reported Tmin&Tmax Range Identical ? Check

PLAT073_ALERT_1_G H-atoms ref, but _hydrogen_treatment Reported as constr Check

PLAT232_ALERT_2_G Hirshfeld Test Diff (M-X) Fe1 --O5 . 8.2 s.u.

And 3 other PLAT232 Alerts

PLAT232_ALERT_2_G Hirshfeld Test Diff (M-X) Fe1 --O6 . 6.6 s.u.

PLAT232_ALERT_2_G Hirshfeld Test Diff (M-X) Fe1 --N1 . 6.0 s.u.

PLAT232_ALERT_2_G Hirshfeld Test Diff (M-X) Fe1 --N2 . 5.6 s.u.

PLAT790_ALERT_4_G Centre of Gravity not Within Unit Cell: Resd. # 2 Note

C2 H3 N

PLAT793_ALERT_4_G Model has Chirality at C8 (Centro SPGR) S Verify

PLAT793_ALERT_4_G Model has Chirality at C13 (Centro SPGR) S Verify

PLAT794_ALERT_5_G Tentative Bond Valency for Fe1 (III) . 2.97 Info

PLAT912_ALERT_4_G Missing # of FCF Reflections Above STh/L= 0.600 211 Note

PLAT960_ALERT_3_G Number of Intensities with I < - 2*sig(I) ... 1 Check

PLAT978_ALERT_2_G Number C-C Bonds with Positive Residual Density. 16 Info

PLAT982_ALERT_1_G The Fe-f'= 0.3582 Deviates from IT-value = 0.3463Check

PLAT983_ALERT_1_G The Fe-f"= 0.8493 Deviates from IT-Value = 0.8444Check

---

0 **ALERT level A** = Most likely a serious problem - resolve or explain

0 **ALERT level B** = A potentially serious problem, consider carefully

4 **ALERT level C** = Check. Ensure it is not caused by an omission or oversight

15 **ALERT level G** = General information/check it is not something unexpected

5 ALERT type 1 CIF construction/syntax error, inconsistent or missing data

6 ALERT type 2 Indicator that the structure model may be wrong or deficient

3 ALERT type 3 Indicator that the structure quality may be low

4 ALERT type 4 Improvement, methodology, query or suggestion

1 ALERT type 5 Informative message, check

It is advisable to attempt to resolve as many as possible of the alerts in all categories. Often the minor alerts point to easily fixed oversights, errors and omissions in your CIF or refinement strategy, so attention to these fine details can be worthwhile. In order to resolve some of the more serious problems it may be necessary to carry out additional measurements or structure

refinements. However, the purpose of your study may justify the reported deviations and the more serious of these should normally be commented upon in the discussion or experimental section of a paper or in the "special_details" fields of the CIF. checkCIF was carefully designed to identify outliers and unusual parameters, but every test has its limitations and alerts that are not important in a particular case may appear. Conversely, the absence of alerts does not guarantee there are no aspects of the results needing attention. It is up to the individual to critically assess their own results and, if necessary, seek expert advice.

Publication of your CIF in IUCr journals

A basic structural check has been run on your CIF. These basic checks will be run on all CIFs submitted for publication in IUCr journals (*Acta Crystallographica*, *Journal of Applied Crystallography*, *Journal of Synchrotron Radiation*); however, if you intend to submit to *Acta Crystallographica Section C* or *E* or *IUCrData*, you should make sure that full publication checks are run on the final version of your CIF prior to submission.

Publication of your CIF in other journals

Please refer to the *Notes for Authors* of the relevant journal for any special instructions relating to CIF submission.

PLATON version of 16/04/2020; check.def file version of 09/03/2020

Datablock sv_452_p21n - ellipsoid plot

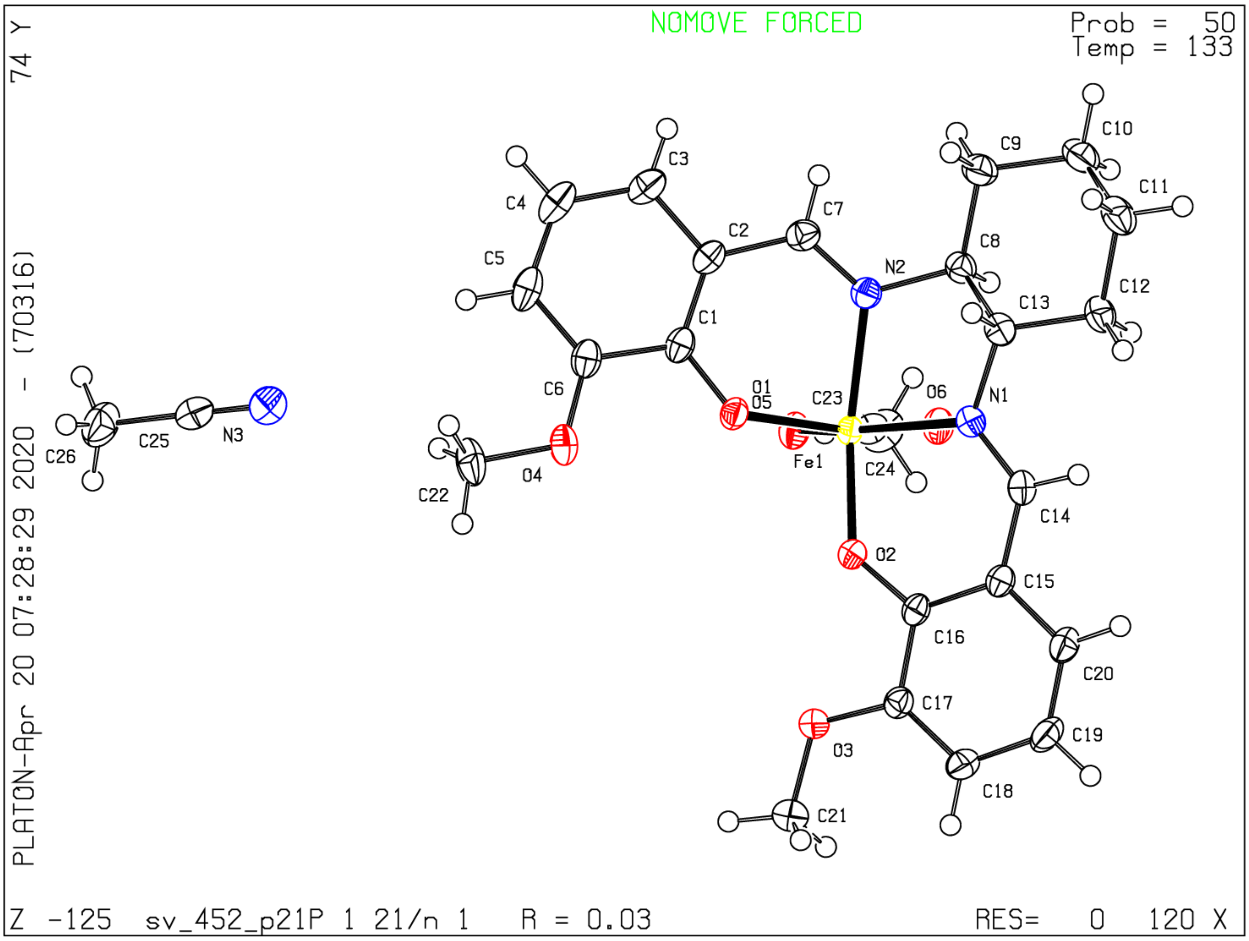

3 References

[1] a) H.-R. Wen, S.-J. Liu, X.-R. Xie, J. Bao, C.-M. Liu and J.-L. Chen, *Inorg. Chim. Acta* **2015,** *435*, 274–282; b) J. S. Elia, M. Risch, L. Giordano, A. N. Mansour and Y. Shao-Horn,. *J. Am. Chem. Soc.* **2014***, 136***,** 17193–17200.

[2] L. F. B. Ribeiro, O. Flores, P. Furtat, C. Gervais, R. Kempe, R. A. F. Machado and G. Motz, *J. Mater. Chem. A.* **2017**, *5*, 720-729.
